# Insight on novel sulfamoylphenyl pyrazole derivatives as anticancer carbonic anhydrase inhibitors

**DOI:** 10.1007/s11030-024-11023-3

**Published:** 2024-11-11

**Authors:** Rehab F. Ahmed, Walaa R. Mahmoud, Nagwa M. Abdelgawad, Amany Belal, Reem I. Alsantali, Mona F. Said

**Affiliations:** 1 Pharmaceutical Chemistry Department, Faculty of Pharmacy, Cairo University, Kasr El-Aini St., Cairo, 11562 Egypt; 2 Department of Pharmaceutical Chemistry, College of Pharmacy, Taif University, P.O.Box 11099, 21944 Taif, Saudi Arabia

**Keywords:** Sulfamoylphenyl, Acetazolamide, Carbonic anhydrase inhibition, Molecular modelling

## Abstract

As another part continue for our previous study, variable substituted pyrazoles bearing sulfamoylphenyl moiety were synthesized and screened against two cancer related human carbonic anhydrase (hCA) isoforms and acetazolamide (AAZ) used as a reference standard. Some compounds as **4e** and **6c** manifested a promising inhibitory activity against both isoforms (K_I_ = 0.072, 0.081 and 0.073, 0.095 µM), respectively. While others as **4a** and **5e** showed inhibitory activity against hCA IX only (K_I_ = 0.062, 0.04 µM) or against hCA XII only as compound **5b** (K_I_ = 0.106 µM) compared to AAZ (K_I_ = 0.065, 0.046 µM), respectively. Also, the anticancer efficacy against 60 cancer cell lines for the target compounds was assessed, and the most promising ones were **4d** and **5a-d**. Further investigation of the anticancer activity of **5b** on MCF-7 cell line explored (IC_50_ = 5.21 µM) compared to doxorubicin (IC_50_ = 11.58 µM). Moreover, compound **5b** was exposed to cell cycle analysis and apoptotic assay on MCF-7 breast cancer cell line under both normal and hypoxic conditions at its IC_50_ concentration with elevation of total apoptotic cells % in MCF-7 relative to the control cells; respectively. Finally, molecular modelling simulations rationalized the in vitro testing results.

**Supplementary Information:**

The online version contains supplementary material available at 10.1007/s11030-024-11023-3.

## Introduction

Carbonic anhydrases (CAs, EC 4.2.1.1), the prevalent zinc-containing metalloprotein that catalyze effectively the reversible transformation of carbon dioxide into bicarbonate with the release of a proton, are vital enzymes that perform a number of crucial physiological and pathological roles in the biological system [1–4]. All human CAs (hCAs) are members of the *α*-class and exist in 16 diverse isoforms of which only 12 are catalytically active [4, 5]. The majority of powerful CAIs have been linked to the presence of an appropriate zinc binding group (ZBG) to create the necessary interactions inside the hCAs active sites [6]. Membrane-bound CA IX is an enzyme whose expression is highly stimulated by hypoxia, a state linked to low oxygen levels in various solid tumor forms, including colon, glioma, and breast cancer [7, 8], while CA XII was identified as tumor related isoform in 1998 [9]. It’s expression is several times higher in cancer and tumor tissues, it is highly expressed in a number of tissues, including the kidney, intestine, reproductive epithelia, and ocular tumors [2, 10]. Regulation of CA XII expression is dependent on estrogen receptors and hypoxia. Low-grade breast cancer is indicated by higher expression of CA XII. CA XII is essential for a number of physiological processes [10].

**Fig. 1:**
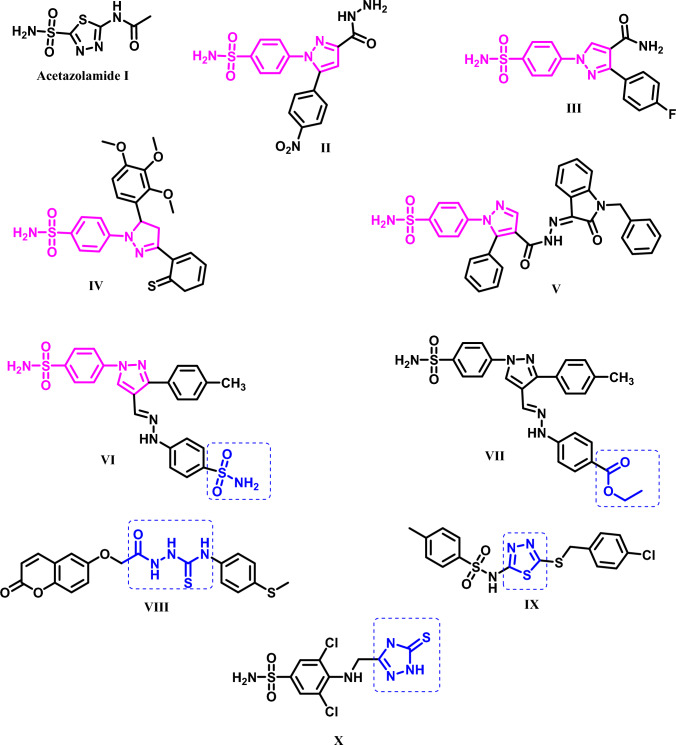
Structures of some reported CAIs

Sulfamoyl moiety is regarded as a cardinal carbonic anhydrase inhibitor pharmacophore as in acetazolamide (**AAZ**)** I**; it is accountable for its coordination with the enzyme metal ion (Zn^2+^) in all isoforms with K_I_ = 25 nM and 5.7 nM against hCA IX and hCA XII; respectively [11, 12]. Accordingly, sulfamoylphenyl scaffold is the most common core in most CAIs [7, 13, 14]. In addition, different pyrazoles bearing sulfamoylphenyl scaffold displayed noteworthy CA inhibition against, hCA IX only as compound **II** with K_I_ value = 2.3 nM relative to the reference drug AAZ [15], against hCA XII only such as compound **III** which is a powerful CA XII inhibitor with K_I_ value of 1.9 nM compared to AAZ (K_I_ value 5.7 nM) on hCA XII, [16] or against both isoforms as compounds **IV** [17] and compound **V** [18] with K_I_ values: (0.044 µM and 13.6 nM) and (0.009 µM and 6.5 nM); respectively, compared to AAZ [19] (Fig. 1).

It is important to specify that the existence of certain functional groups had favourable effect on CA inhibition such as AAZ **I**; the well-known CAI [11, 12], sulfamoyl group or its ester congener [20], acylthiosemicarbazide [21], 1,3,4-thiadiazole [22] or 1,2,4-triazoles [23, 24] 5-memebered rings as shown in compounds **I**, **VI-X**, individually (Fig. 1).

In the current research, we are keeping our former attempts [20] to explore novel anticancer sulfamoylphenyl pyrazoles adopted the required features to inhibit the cancer related CA isoforms; hCA IX and hCA XII.

**Fig. 2:**
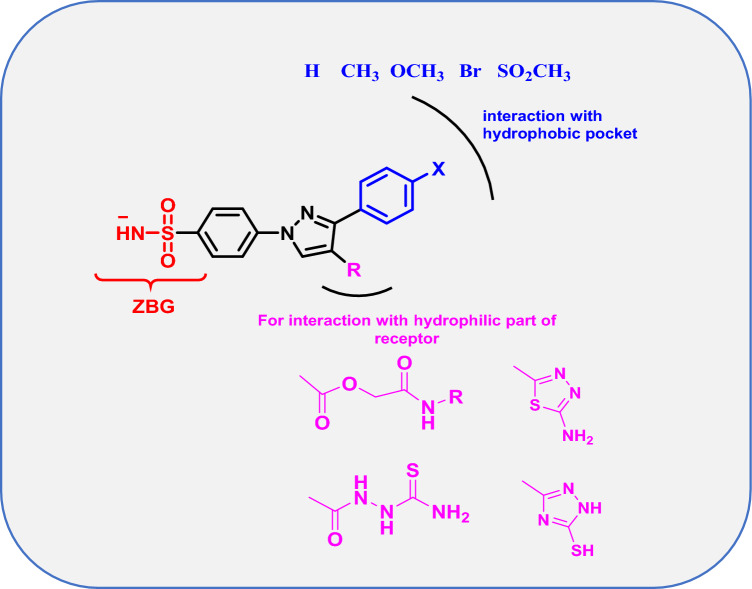
General structure of the target compounds

We focused on attaching different moieties at position 4 of pyrazole of sulfamoylphenyl pyrazole scaffold to perform the required interactions with the hydrophilic half of the receptor using either 5 or 6- membered aromatic ring directly attached or through a five-atom spacer. On the other hand, the receptor is accommodated with *p*-substituted phenyl moiety at position 3 of the pyrazole ring to accommodate the hydrophobic half of the receptor (Fig. 2).

Moreover, molecular modelling was performed to explore types of interaction of the most active compounds in the vicinity of CA IX and CA XII active sites for better understanding of their biological results.

## Results and discussion

### Chemistry

**Scheme 1:**
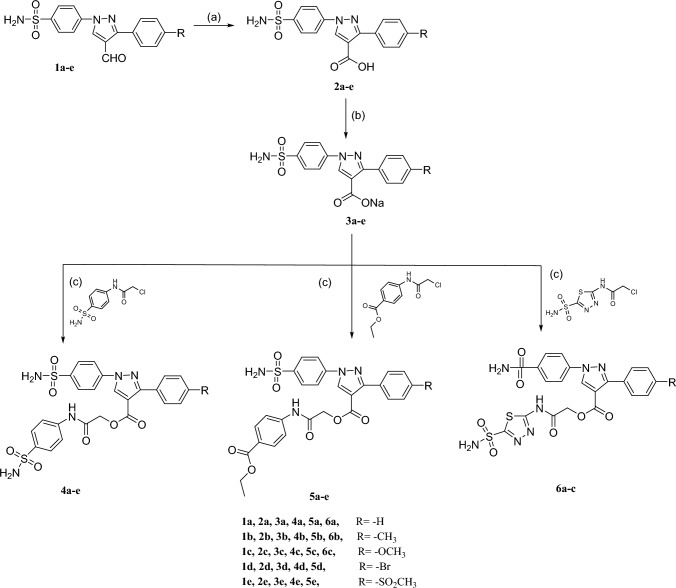
Reagents and reaction conditions: **a** KMnO_4_, pyridine, 0 °C, **b** Sod. metal, absolute methanol, reflux 25–30 h, **c** Different acetamido derivatives, DMF, reflux

**Scheme 2:**
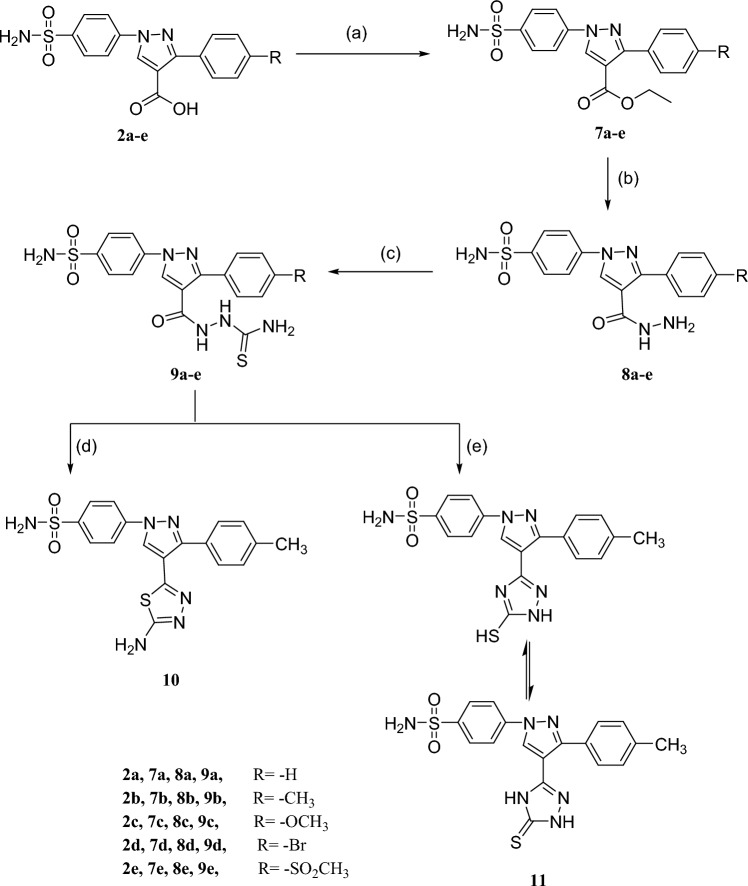
Reagents and reaction conditions: **a** Absolute ethanol, conc. H_2_SO_4_, reflux **b** NH_2_NH_2_, reflux, **c** Ammonium thiocyanate, 10% HCl, absolute ethanol, reflux,** d 9b**, H_2_SO_4_, 90 °C, 2h, **e 9b**, 1% NaOH, ethanol, reflux

The synthetic routes for the final compounds are depicted in schemes 1 and 2. Initially, the 4-substituted pyrazole carboxylic acid derivatives **2a-e** were prepared via the oxidation of the corresponding 4-formyl pyrazole derivatives **1a-e** [25, 26] with potassium permanganate to give **2a-e** in good yields [16, 20, 27, 28]. Final target compounds were achieved through the condensation of the sodium carboxylate salts **3a-e** with different chloroacetamido derivatives namely 2-chloro-*N*-(4-sulfamoylphenyl) acetamide, ethyl 4-(2-chloroacetamido) benzoate and 2-chloro-*N*-(5-sulfamoyl-1,3,4-thiadiazol-2-yl) acetamide to furnish **4a-e**, **5a-e** and **6a-c**; respectively (Scheme 1).

In scheme 2, the hydrazine carbothioamides **9a-e** were achieved via the reaction of the corresponding acid hydrazide derivatives **8a-e** with ammonium thiocyanate in absolute ethanol containing 10% HCl. Then, the acylthiosemicarbazide derivatives **9a-e** were cyclized under acidic or basic conditions to give the cyclized derivatives **10** and **11**; respectively.

All the newly prepared derivatives were confirmed through spectral and elemental analyses as illustrated in details in the experimental part.

### Biological assays

#### CA inhibitory activity

The CA inhibitory test conducted in vitro was used to assess the activity of the synthesized target compounds **4a-e**, **5a-e**, **6a-c**, **9a-e**, **10** and **11** as well as the reference standard AAZ against the cancer-related isoforms CA IX and CA XII in order to explore their potentiality as anticancer derivatives. The IC_50_ (µM) values of the examined compounds and AAZ against hCA IX and hCA XII are listed in Table 1.

**Table 1:** IC_50_ (µM) of the final compounds and AAZ against hCA IX and hCA XII

Compound	IC_50_ (µM) ± SD		Compound	IC_50_ (µM) ± SD	
	hCA IX	hCA XII		hCA IX	hCA XII
**4a**	0.062 ± 0.003*	0.226 ± 0.009	**6a**	0.591 ± 0.028	0.358 ± 0.013
**4b**	0.331 ± 0.015	0.17 ± 0.006	**6b**	0.339 ± 0.016	1.7 ± 0.064
**4c**	0.175 ± 0.008	0.415 ± 0.016	**6c**	0.073 ± 0.003*	0.095 ± 0.004*
**4d**	0.509 ± 0.024	0.814 ± 0.031	**9a**	0.215 ± 0.01	0.605 ± 0.023
**4e**	0.072 ± 0.003*	0.081 ± 0.003*	**9b**	0.339 ± 0.019	0.335 ± 0.013
**5a**	0.945 ± 0.044	0.395 ± 0.015	**9c**	0.365 ± 0.017	0.168 ± 0.006
**5b**	0.148 ± 0.007	0.106 ± 0.004*	**9d**	1.108 ± 0.052	1.417 ± 0.053
**5c**	0.22 ± 0.01	0.272 ± 0.01	**9e**	1.77 ± 0.083	0.929 ± 0.035
**5d**	0.631 ± 0.029	0.99 ± 0.037	**10**	0.663 ± 0.031	1.148 ± 0.043
**5e**	0.04 ± 0.002*	0.167 ± 0.006	**11**	0.24 ± 0.011	0.332 ± 0.013
AAZ	0.065 ± 0.003	0.046 ± 0.002			

*IC_50_ results that are considered not significantly different from that of AAZ at (*P* < 0.05)

Five of the tested compounds were non-significantly different from AAZ in inhibiting either hCA IX only as compounds **4a** and **5e** (0.06 and 0.04 µM) or hCA XII only as compound **5b** (0.10 µM) or both isoforms like compounds **4e** and **6c** (0.07 µM), (0.08 and 0.09 µM); respectively, compared to AAZ (0.065 and 0.046 µM), respectively.

The proposed compounds showed inhibitory action against hCA IX, with an IC_50_ ranging from 0.04 to 1.77 µM. The ester derivatives **4a**, **4e**, **5b**, **5e** and **6c** showed high potency on hCA IX with IC_50_ (0.04 to 0.07 µM) and compound **5e** elicited the most effective inhibitory activity with IC_50_ = 0.04 µM relative to AAZ (IC_50_ = 0.06 µM). The unsubstituted derivatives **4a** and the derivatives with electron withdrawing substitutions, e.g., SO_2_CH_3_ or Br **5e** and **6c**; respectively, showed the best inhibitory activity with (IC_50_ = 0.06, 0.04 and 0.07 µM; respectively).

Compounds **4a-e** with IC_50_ (0.06–0.50 μM) showed good inhibitory activity, where the unsubstituted derivative **4a** become the most active one in this series with IC_50_ (0.06 μM) and the activity of *p*-methyl derivative **4b** is slightly decreased with IC_50_ (0.33 μM), by replacing the methyl group with methoxy one in **4c**, the activity is enhanced with IC_50_ (0.17 μM). While the activity in the derivatives with electron withdrawing group **4d** and **4e** is enhanced with IC_50_ (0.50 and 0.07 μM, respectively). Moreover, in compounds **5a-e**, the tested compounds exhibited inhibitory activity with IC_50_ ranging from 0.04 to 3.55 µM. Compound **5e** elicited the most potent inhibitory activity with IC_50_ of 0.04 µM comparable to that of AAZ (IC_50_ = 0.05 µM). In compounds **6a-c** showed good inhibitory activity with IC_50_ (0.07–0.59 μM), where the *p*-methoxy derivative **6c** was the most active compound. Furthermore, the acylthiosemicarbazide derivatives **9a-e** with IC_50_ (0.21–1.77 µM), the unsubstituted derivative **9a** showed good inhibitory activity with IC_50_ (0.21 µM) and in compounds with electron donating groups **9b** and **9c** with IC_50_ (0.33 and 0.36 µM, respectively) the activity is slightly decreased, while the activity is decreased in derivatives with electron withdrawing groups **9d** and **9e** with IC_50_ (1.18 and 1.77 µM, respectively). Cyclization of the acylthiosemicarbazide derivative **9b** IC_50_ (0.33 µM) into thiadiazol one **10** led to decrease in activity with IC_50_ (0.66 µM), while cyclization to triazol congener **11** led to increase in the activity with IC_50_ (0.24 µM).

On the other hand, concerning inhibition of hCA XII, the tested compounds showed suppressive effect with IC_50_ values range from 0.08 to 1.70 µM. The ester derivative **4e** revealed the best activity with IC_50_ of 0.08 µM compared to that of AAZ (IC_50_ = 0.04 µM). Compounds **4e**, **5b** and **6c** demonstrated the best inhibitory activity compared to most of the series.

In compounds **4a-e** IC_50_ (0.08–0.81 µM), showed good inhibitory activity, where the CH_3_SO_2_ substituted derivative **4e** become the most active derivative in this series with IC_50_ (0.08 µM) and the activity of the *p*-methyl derivative **4b** is slightly decreased with IC_50_ (0.17 µM), the activity increased in the unsubstituted derivative **4a**, the *p-*methoxy derivative **4c** and the bromo derivative **4d** with IC_50_ (0.22, 0.41 and 0.81, respectively µM).

Furthermore, in the ester derivatives **5a-e** IC_50_ (0.10–0.99 µM), the activity of the *p*-methoxy derivative **5c** and of the CH_3_SO_2_ derivative **5e** is decreased with IC_50_ (0.27 and 0.16 µM, respectively). The *p*-methyl derivative **5b** was the most active among this series with IC_50_ (0.10 µM) and by removing this methyl group as in compound **5a** or replacing it with a bromo group in **5d**, the activity is decreased with IC_50_ (0.39 and 0.99 µM, respectively).

In **6a-c**, the unsubstituted derivative **6a** and the *p*-methyl derivative **6b** elicited inhibitory activity with IC_50_ (0.35 and 1.70 µM, respectively). While the methoxy derivative **6c** was the most potent in this series with IC_50_ (0.09 µM).

In acylthiosemicarbazide derivatives **9a-e** showed good inhibitory activity with IC_50_ (0.16–1.41 µM). Cyclization of the acylthiosemicarbazide derivative **9b** IC_50_ (0.33 µM) into thiadiazole ring **10** led to decrease in activity with IC_50_ (1.14 µM), while cyclization to the triazole one **11** showed the same activity as **9b** with IC_50_ (0.33 µM).

#### Anticancer activity

##### Preliminary anticancer screening at a single dose (10 µM)

The National Cancer Institute (NCI) Developmental Therapeutic Program (www.dtp.nci.nih.gov) selected the 20 novel synthesized compounds **4a-e**, **5a-e**, **6a-c**, **9a-e**, **10** and **11** to test for their anticancer activity at a single dose of 10 µM using SRB assay. Results are demonstrated as percentage growth inhibition (GI %) as well as mean graph of the growth percentage. A closer look on the in vitro preliminary anticancer screening revealed that best GI % against the tested cancer panels were for the ester compounds **4d** and **5a-d** with obvious clustered anti-cancer activity for compounds **5a-d**. Compound **4d** exhibited good activity over the panels under investigation with growth inhibition ranging from 8.46 to 86.55%. Interestingly, compounds **5a-d** revealed significant anticancer activity against all cancer types with GI % ranges (5.67–92.08%), (12.78–83.81%), (5.25–65.90%) and (13.33–93.98%), respectively. Supplementary Data S3; Table S1 presents the detailed screening results of the most promising compounds. The preliminary screening’s detailed results for the remaining compounds are elucidated in the (Supplementary Data S3; Table S2).

#### In vitro cytotoxic activity against MCF-7

In an attempt to find a suitable correlation between the effect on certain tumor type and its mechanism of action, compound **5b** as one of the most promising derivatives in the NCI screening along with its significant inhibitory results on hCA XII was selected for further biological evaluation. MCF-7 breast cancer cell line was chosen being one of the most cancer types where hCA XII is overexpressed from one side and from the other side compound **5b** exhibited excellent inhibitory activity on it. Compound **5b** was tested for its in vitro cytotoxicity against MCF-7 breast cancer cell line by using MTT assay under normal and hypoxic conditions. The results shown in Table 2 and 3 under both normal and hypoxic conditions, respectively, displayed significant inhibitory activity compared to doxorubicin as a reference (IC_50_ = 5.21 and 11.58 µM); respectively under normal conditions and under hypoxic conditions** (**IC_50_ = 3.50 and 2.72 µM); respectively indicating its significant anticancer activity.

**Table 2:** Cytotoxic activity of compound **5b** and doxorubicin against MCF-7 breast cancer cell line under normal conditions

Compound	IC_50_ (µM) ± SD
**5b**	5.21 ± 0.21
Doxorubicin	11.58 ± 0.46

**Table 3:** Cytotoxic activity of compound **5b** and doxorubicin against MCF-7 breast cancer cell line under hypoxia

Compound	IC_50_ (µM) ± SD
**5b**	3.50 ± 0.14
Doxorubicin	2.72 ± 0.11

#### Cell cycle analysis and apoptotic assay

The promising inhibitory results of compound **5b** on hCA XII along with its significant effect on MCF-7 breast cell line was a determined factor for further investigation of its biological activity via cell cycle analysis and apoptotic activity under normal and hypoxic conditions.

##### Cell cycle analysis

**Fig. 3:**
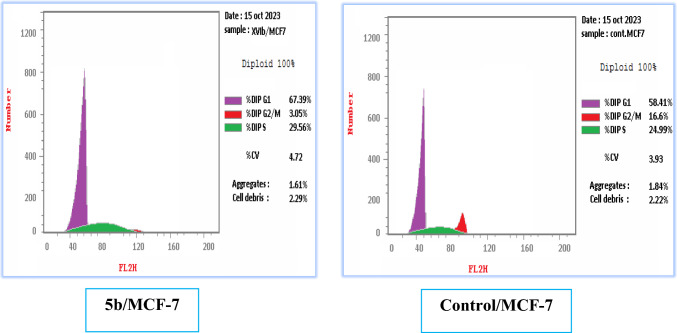
Influence of compound **5b** on the cell cycle of MCF-7 cells after 24 h compared to the control under normal conditions

**Fig. 4:**
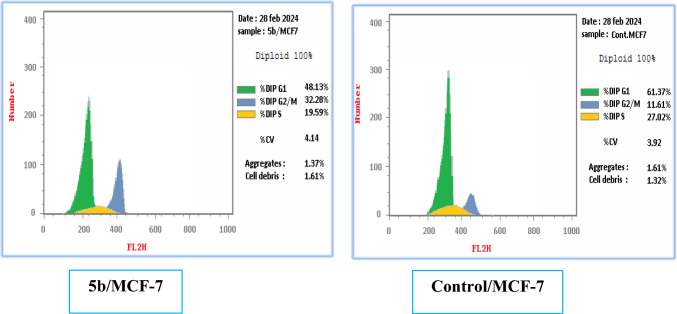
Influence of compound **5b** on the cell cycle of MCF-7 cells after 24 h compared to the control under hypoxic conditions

In this part, a flow cytometric analysis of compound **5b** was performed on MCF-7 under both normal and hypoxic conditions at the specified IC_50_ (5.21 and 3.50 µM); respectively, to investigate its impact on the cell cycle development. The outcomes under normal conditions depicted in Table 4 and Fig. 3, showed that derivative **5b** affected mainly cell growth arrest at G1/S phase by increasing the cellular population from 58.41 to 67.39% (0.87 folds) compared to the control along with significant reduction at the G2/M phase from 16.6 to 3.05% (5.44 folds). While the results under hypoxic conditions depicted in Table 5 and Fig. 4, showed that derivative **5b** affected mainly cell growth arrest at G2/M phase by increasing the cellular population from 11.61 to 32.28% (0.36 folds) compared to the control along with significant reduction at the S phase from 27.02 to 19.59% (1.38 folds).

**Table 4:** Cell cycle analysis results of **5b** against MCF-7 cell line under normal conditions

	DNA content			Cell growth arrest at
	% G0-G1	% S	% G2/M	
**5b**/MCF-7	67.39	29.56	3.05	G1/S
Control/MCF-7	58.41	24.99	16.6	–

**Table 5:** Cell cycle analysis results of **5b** against MCF-7 cell line under hypoxic conditions

	DNA content			Cell growth arrest at
	% G0-G1	% S	% G2/M	
**5b**/MCF-7	48.13	19.59	32.28	G2/M
Control/MCF-	61.37	27.02	11.61	–

##### Apoptotic effect

**Fig. 5:**
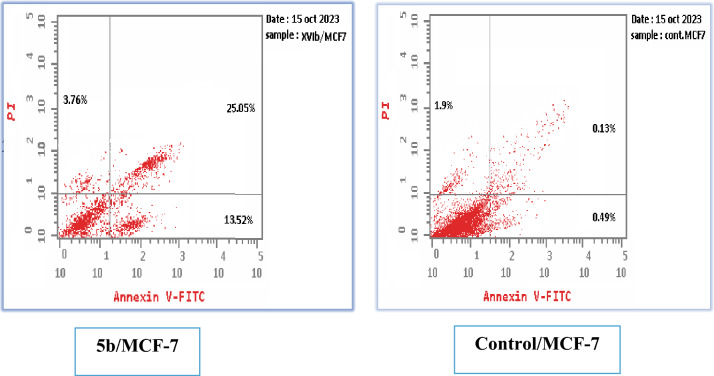
Results of the apoptotic and necrotic effects of derivative **5b** compared to the control cells under normal conditions

**Fig. 6:**
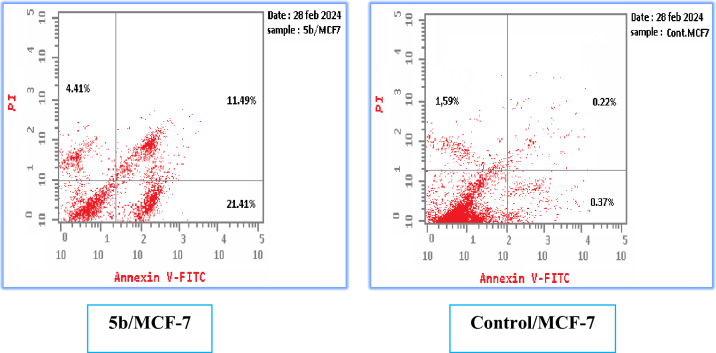
Results of the apoptotic and necrotic effects of derivative **5b** compared to the control cells under hypoxic conditions

The apoptotic assay was conducted utilizing the Annexin V-FITC/propidium iodide technique to explore the cell apoptosis caused under the effect of the promising compound **5b** on MCF-7 breast cancer cells in accordance with the reported method [29, 30]. The effect of compound **5b** on the apoptotic liability in the selected cell line under normal condition is demonstrated in Table 6 and Fig. 5. These results showed an increase in the total of the apoptotic cells after the inclusion of compound **5b** in MCF-7 cell line (42.33%) as well as the necrotic cells (3.76%) relative to the control cells (2.52 and 1.9%, respectively). Added to that, investigating compound **5b** under hypoxic conditions, it showed an elevation in the total % of the apoptotic cells in MCF-7 cell line (37.31%) as well as the necrotic cells (4.41%) relative to the control cells (2.18 and 1.59%; respectively) as shown in Table 7 and Fig. 6. These findings may suggest compound **5b** as a potential inducer of apoptosis.

**Table 6:** Impact of compound **5b** on apoptotic creation on MCF-7 relative to the control cell under normal conditions

Compound	Apoptosis %			Necrosis %
	Total %	Early %	Late %	
**5b**/MCF-7	42.33	13.52	25.05	3.76
Cont.MCF-7	2.52	0.49	0.13	1.9

**Table 7:** Impact of compound **5b** on apoptotic creation on MCF-7 relative to the control cell under hypoxic conditions

Compound	Apoptosis %			Necrosis %
	Total %	Early %	Late %	
**5b**/MCF-7	37.31	21.41	11.49	4.41
Cont.MCF-7	2.18	0.37	0.22	1.59

### In silico studies

#### Molecular modelling

Among the synthesized series, compounds **4a**, **4e**, **5e** and **6c** showed the highest hCA IX inhibitors, based on the in vitro carbonic anhydrase IC50 values. While compounds **4e**, **5b** and **6c** elicited the best hCA XII inhibition compared to AAZ (IC_50_ = 0.04 µM). Consequently, molecular docking study on the specified compounds in the hCA IX and hCA XII active sites was done.

In the present study, the thiophene sulfamoyl inhibitor **(9FK)** [31] and **AAZ** [32] co-crystallized with hCA IX and hCA XII (PDB IDs: 5FL4 and 1JD0, respectively) were employed. Validating the molecular docking approach using self-docking was demonstrated by the minimal RMSD values between the re-docked poses in hCA XII (1.0507 Å) and hCA IX (1.0810 Å) and the native co-crystallized ligands. Additionally, the appropriateness of the suggested protocol was validated by the ability of the naturally co-crystallized ligands **(9FK)** and the experimentally employed reference **AZZ**) to replicate every significant interaction it possessed with the hot areas in CA IX and CA XII active sites; respectively.

The most important docking parameters; energy scores (Kcal/mol), binding interaction, hydrogen bond length compared to their IC_50_ values compared to the co-crystallized ligands in the hot spot of hCA IX and hCA XII isozymes were displayed in (Supplementary data: S.6.2, Table: S3 and S4), respectively.

Regarding hCA IX, the newly designed compounds showed comparable binding modes in the hot spot of hCA IX, which entail the interaction of the sulfamoyl moiety as the ZBG with the Zn^+2^ ion along with the creation of hydrogen bond with the crucial amino acid Thr200. The pyrazole ring and the hydrophobic side chain of the amino acid Gln71 make another hydrophobic interaction in compound **4a** as depicted in the (Supplementary data: S.6.3, Figure: S66).

**Fig. 7:**
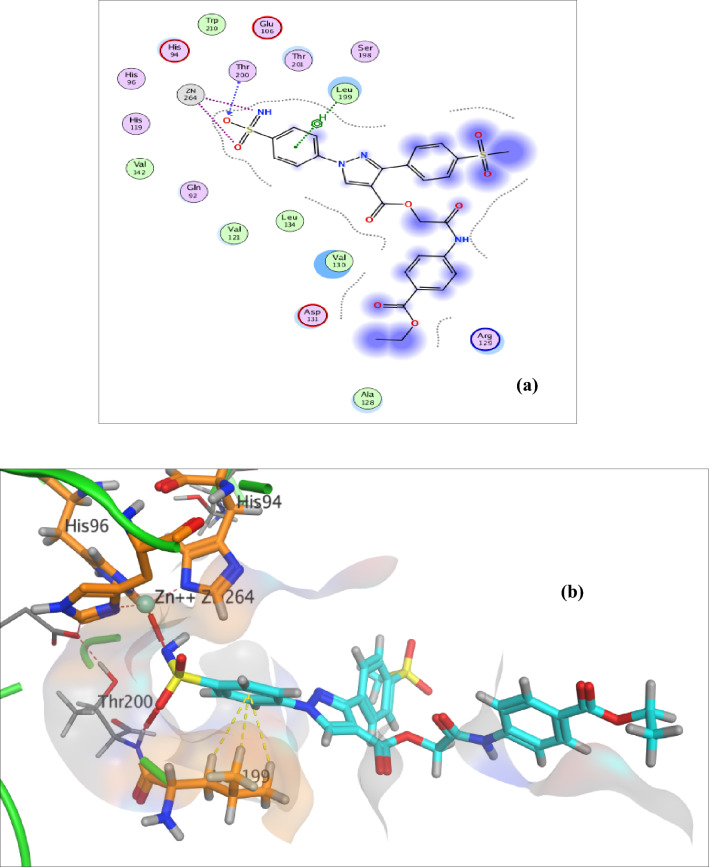
2D **a** and 3D **b** interaction of compound **5e** in the hCA IX binding site

**Fig. 8:**
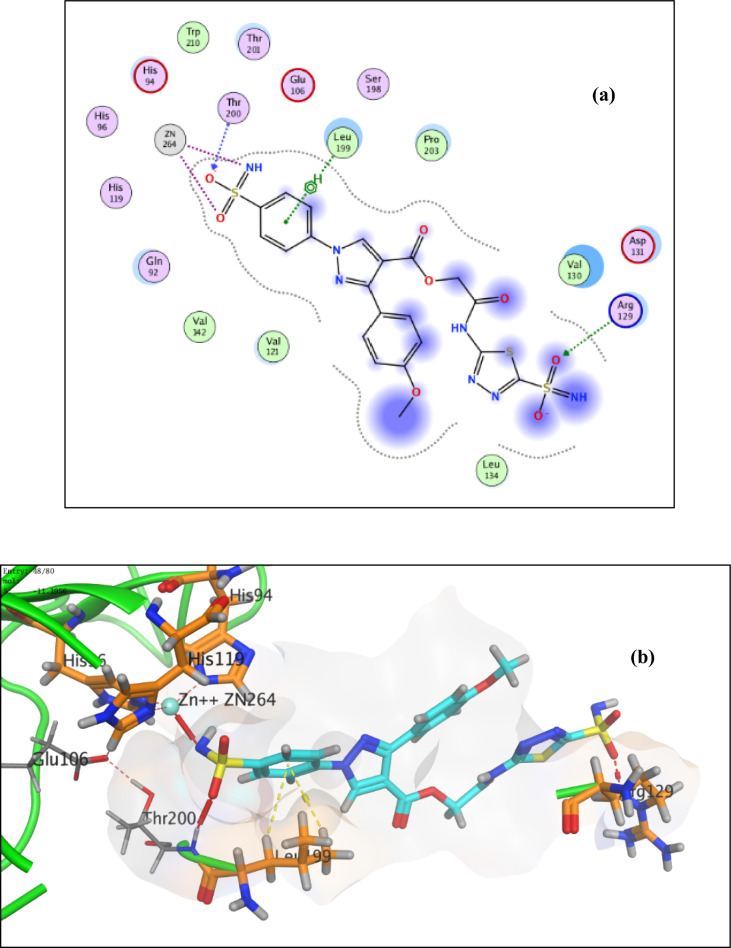
2D **a** and 3D **b** interaction of compound **6c** in the hCA IX binding site

Regarding the ester derivatives **4e**, **5e** and **6c**, the sulfamoyl phenyl ring establish hydrophobic contact with the hydrophobic side chain of the amino acid Leu199 (Supplementary data: S.6.3, Figure S67), Fig. 7 and 8, respectively. Also, extra two additional hydrogen bonds between the nitrogen of the pyrazole ring and the side chain amino group of His68 and between NHCO and the carbonyl side chain of the amino acid Gln71 are well characterized as in compound **4a **(Supplementary data: S.6.3, Figure S66). Also, it is obvious that an extra hydrogen bond between the oxygen of the sulfamoyl of acetazolamide moiety and sidechain amino group of the amino acid Arg129 was accomplished as in compound **6c **(Fig. 8). Furthermore, a hydrophobic interaction is formed between the hydrophobic side chain of the amino acid Leu91 and the benzene ring as in compounds **4e **(Supplementary data: S.6.3, Figure: S67).

**Fig. 9:**
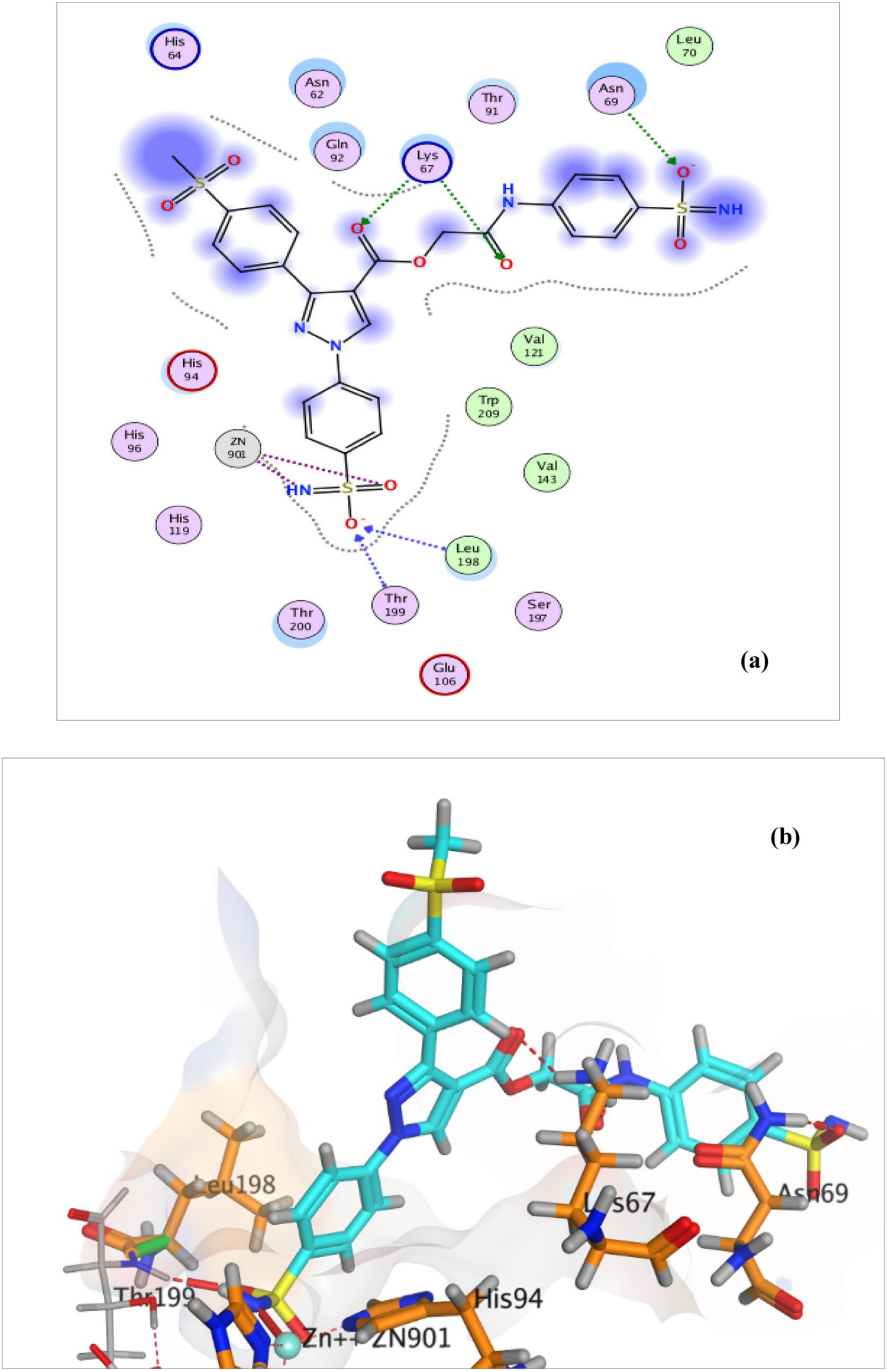
2D **a** and 3D **b** interaction of compound **4e** in the hCA XII binding site

**Fig. 10:**
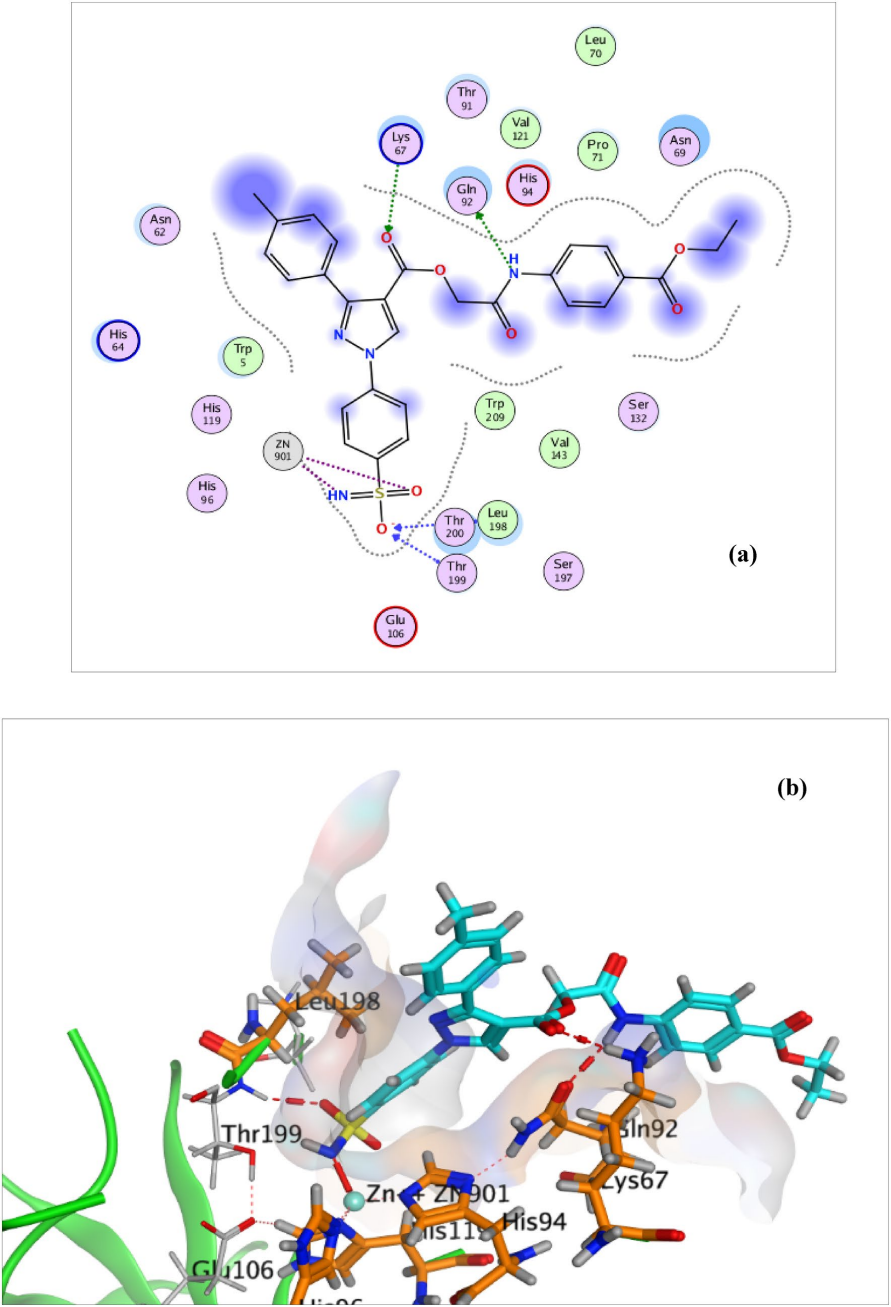
2D **a** and 3D **b** interaction of compound **5b** in the hCA XII binding site

Concerning hCA XII, all the synthesized candidates elicited three major interactions: with Zn^+2^ ion at the active site, Leu198 and Thr199. An additional hydrogen bond is formed between compounds **4e**, **5b** and **6c** and the side chain amino group of the amino acid Lys67 **(**Fig. 9, 10)asis> and (Supplementary data: S.6.3, Figure S68), respectively. Furthermore, an extra hydrogen bond was achieved in compounds **4e** and **5b** between the amino acids Asn69 and Gln 92 side chain carbonyl group with oxygen of the added sulfamoyl moiety and NH group; respectively. Also, compound **6c** forms two extra hydrogen bonds between the NH and oxygen of sulfamoyl of acetazolamide with the amino acid His64 and Lys170, respectively, as depicted in the (Supplementary data: S.6.3, Figure S68); respectively.

#### Toxicity prediction

The newly synthesized compounds **4a**, **4e**, **5b**, **5e** and **6c** were examined by the web server; Osiris Property Explorer (http://www.organic-chemistry.org/prog/peo/) to investigate their toxicities. This web server’ prediction is relied on how well the tested compound’s functional group match the precomputed set of structural fragments that are included in its database. Prediction results are demonstrated as colors; red, green, and yellow. Whereby, red color assumes a high risk of toxicity, yellow represents mild toxicity and the green color suggests low toxic effect [33]. The findings elucidated that all the tested candidates are anticipated to be safe and show no toxicity in terms of mutagenicity, tumorigenicity, irritating effect, or influence on the reproductive system except compound **6c** which has toxic effect on the reproductive system.

## Conclusion

Twenty pyrazole derivatives bearing sulfamoylphenyl moiety were designed via tail approach strategy where the sulfamoylphenyl pyrazole scaffold as ZBG was 3,4-disubstituted with suitable hydrophobic and hydrophilic moieties; respectively to accommodate the diverse nature of the enzyme active site halves. The IC_50_ of compound **5b** (0.10 µM) were non-significantly different from that of AAZ as hCA XII inhibitors. The results of the anticancer screening showed that derivatives **4d** and **5a-d** had a promising anticancer activity against all the tumor cell lines in the panel. Also, compound **5b** elicited an increment of the total apoptotic cells % in MCF-7 under both normal and hypoxic conditions (42.33 and 37.31%) compared to the control cells (2.52 and 2.18%); respectively. The in silico molecular modelling simulation indicated critical interaction pattern with the active site of carbonic anhydrase which might explain the obtained activity. On the other hand, the toxicity prediction revealed that the synthesized compounds are anticipated to be safe exhibiting no toxicity in terms of mutagenicity, tumorigenicity, irritability, or reproductive system effects. These encouraging results open the door for further investigation of the synthesized derivatives and introduce compound **5b** as potential anticancer compound via hCA XII inhibition.

## Experimental

### Chemistry

The intense steps of the methodology used for the chemical syntheses of the given compounds are presented in (Supplementary data, S1.1.)

Based on the published literature, the following starting and intermediate compounds were synthesized: **1a-e** [26], **2a-e** [16, 20, 27, 28], **7a-e** [20] and **8a-e**.[16, 20, 34]

#### General procedure for the synthesis of compounds (3a-e)

Sodium methoxide solution (1 mol Na metal dissolved in 15 mL absolute methanol) was added to the appropriate acid **2a-e** (1 mol) dissolved in 10 mL absolute methanol. The reaction mixture was refluxed for 25–30 h. The formed precipitate was washed by diethyl ether and then filtered to give the target compounds.

##### Sodium 3-phenyl-1-(4-sulfamoylphenyl)-1H-pyrazole-4-carboxylate (3a)

Yield: 85%, m.p. 145–150 °C. R_f_ = 0.17 (CHCl_3_, methanol). IR (KBr) υ_max_/cm^−1^: 3363, 3300 (NH_2_), 3062 (CH Ar), 1690 (C=O), 1589 (NH bending), 1535, 1504 (C=C), 1365, 1165 (SO_2_). ^1^H NMR (400 MHz, DMSO-*d*_*6*_) δ ppm: 7.36- 7.47 (m, 5H, Ar H), 7.94 (s, 2H, NH_2_, D_2_O exchangeable), 8.09–8.17 (m, 4H, Ar H), 8.81 (s, 1H, CH pyrazole). ^13^C NMR (100 MHz, DMSO-*d*_*6*_) δ ppm: 118.4, 127.4, 127.7, 127.9, 128.1, 129.2, 129.5, 133.9, 141.6, 142.0, 152.5 (C Ar), 167.8 (C=O).

##### Sodium 1-(4-sulfamoylphenyl)-3-(p-tolyl)-1H-pyrazole-4-carboxylate (3b)

Yield: 88%, m.p. 279–280 °C. R_f_ = 0.20 (CHCl_3_, methanol). IR (KBr) υ_max_/cm^−1^: 3300, 3294 (NH_2_), 3066 (CH Ar), 2924, 2850 (CH aliph.), 1690 (C=O), 1597 (NH bending), 1558, 1508 (C=C), 1365, 1161 (SO_2_). ^1^H NMR (400 MHz, DMSO-*d*_*6*_) δ ppm: 2.34 (s, 3H, CH_3_), 7.16 (d, *J* = 7.96 Hz, 2H, Ar H), 7.38 (s, 2H, NH_2_, D_2_O exchangeable), 7.90 (d, *J* = 8.68 Hz, 2H, Ar H), 8.08–8.10 (m, 4H, Ar H), 8.56 (s, 1H, CH pyrazole). ^13^C NMR (100 MHz, DMSO-*d*_*6*_) δ ppm: 21.3 (CH_3_), 118.3, 125.7, 127.7, 128.4, 129.3, 131.2, 131.6, 137.1, 141.4, 142.0, 151.8 (C Ar), 167.2 (C=O).

##### Sodium 3-(4-methoxyphenyl)-1-(4-sulfamoylphenyl)-1H-pyrazole-4-carboxylate (3c)

Yield: 90%, m.p. > 300 °C. R_f_ = 0.18 (CHCl_3_, methanol). IR (KBr) υ_max_/cm^−1^: 3441, 3383 (NH_2_), 3082 (CH Ar), 2935, 2835 (CH aliph.), 1704 (C=O), 1597 (NH bending), 1577, 1527 (C=C), 1346, 1165 (SO_2_). ^1^H NMR (400 MHz, DMSO-*d*_*6*_) δ ppm: 3.79 (s, 3H, OCH_3_), 6.91 (d, *J* = 8.84 Hz, 2H, Ar H), 7.78 (d, *J* = 8.68 Hz, 2H, Ar H), 7.85 (d, *J* = 8.60 Hz, 2H, Ar H), 7.92 (s, 2H, NH_2_, D_2_O exchangeable), 8.18 (d, *J* = 8.84 Hz, 2H, Ar H), 8.48 (s, 1H, CH pyrazole). ^13^C NMR (100 MHz, DMSO-*d*_*6*_) δ ppm: 55.5 (OCH_3_), 113.2, 117.6, 124.2, 126.7, 127.0, 130.7, 131.8, 139.9, 148.3, 151.1, 159.2 (C Ar), 168.0 (C=O).

##### Sodium 3-(4-bromophenyl)-1-(4-sulfamoylphenyl)-1H-pyrazole-4-carboxylate (3d)

Yield: 78%, m.p. 289–291 °C. R_f_ = 0.22 (CHCl_3_, methanol). IR (KBr) υ_max_/cm^−1^: 3383, 3313 (NH_2_), 3070 (CH Ar), 1690 (C=O), 1597 (NH bending), 1558, 1527 (C=C), 1365, 1161 (SO_2_). ^1^H NMR (400 MHz, DMSO-*d*_*6*_) δ ppm: 7.48 (s, 2H, NH_2_, D_2_O exchangeable), 7.58 (d, *J* = 8.28 Hz, 2H, Ar H), 7.94 (d, *J* = 8.40 Hz, 2H, Ar H), 8.09–8.16 (m, 4H, Ar H), 8.84 (s, 1H, CH pyrazole). ^13^C NMR (100 MHz, DMSO-*d*_*6*_) δ ppm: 118.8, 121.8, 130.2, 130.9, 131.1, 131.5, 132.0, 133.0, 141.7, 142.1, 151.2 (C Ar), 166.5 (C=O).

##### Sodium 3-(4-(methylsulfonyl) phenyl)-1-(4-sulfamoylphenyl)-1H-pyrazole-4-carboxylate (3e)

Yield: 85%, m.p. 125–129 °C. R_f_ = 0.18 (CHCl_3_, methanol). IR (KBr) υ_max_/cm^−1^: 3402, 3250 (NH_2_), 3020 (CH Ar), 2924, 2831 (CH aliph.), 1690 (C=O), 1593 (NH bending), 1527, 1504 (C=C), 1361, 1149 (SO_2_). ^1^H NMR (400 MHz, DMSO-*d*_*6*_) δ ppm: 3.23 (s, 3H, CH_3_SO_2_), 7.92 (d, *J* = 8.24 Hz, 6H, Ar H and NH_2_, D_2_O exchangeable), 8.07 (d, *J* = 8.60 Hz, 2H, Ar H), 8.46 (d, *J* = 8.46 Hz, 2H, Ar H), 8.77 (s, 1H, CH pyrazole). ^13^C NMR (100 MHz, DMSO-*d*_*6*_) δ ppm: 44.1 (CH_3_SO_2_), 118.7, 125.0, 126.7, 127.7, 130.2, 132.7, 138.8, 139.8, 141.4, 142.9, 150.4 (C Ar), 167.5 (C=O).

#### General procedure for the synthesis of compounds (4a-e), (5a-e) and (6a-e)

Equimolar amounts of **3a-e** (1 mmol) and 2**-**chloro-*N*-(4-sulfamoylphenyl) acetamide (0.24 gm, 1 mmol), ethyl 4-(2-chloroacetamido) benzoate (1 mmol, 0.23 gm) or 2-chloro-*N*-(5-sulfamoyl-1,3,4-thiadiazol-2-yl) acetamide (0.25 gm, 1 mmol) were dissolved in DMF and refluxed. After reaction completion as monitored by TLC, the mixture was poured on ice-water, the solid formed was filtered off, washed with cold water and crystallized from ethanol to give target compounds **(4a-e)**, **(5a-e)** and **(6a-e)**, respectively.

##### 2-Oxo-2-[(4-sulfamoylphenyl) amino] ethyl 3-phenyl-1-(4-sulfamoylphenyl)-1H-pyrazole-4-carboxylate (4a)

Yield: 73%, m.p. 160–162 °C. R_f_ = 0.54 (CHCl_3_, methanol). IR (KBr) υ_max_/cm^−1^: 3302, 3271, 3128 (NH_2_, NH), 3066 (CH Ar), 1705, 1690 (2 C=O), 1597 (NH bending), 1535, 1504 (C=C), 1334, 1157 (SO_2_). ^1^H NMR (400 MHz, DMSO-*d*_*6*_) δ ppm: 4.90 (s, 2H, CH_2_), 7.28 (s, 2H, NH_2_, D_2_O exchangeable), 7.46–7.49 (m, 4H, Ar H), 7.75–7.81 (m, 3H, Ar H), 7.86 (d, *J* = 5.00 Hz, 2H, Ar H), 7.95 (s, 2H, NH_2_, D_2_O exchangeable), 8.00 (d, *J* = 8.44 Hz, 2H, Ar H), 8.25 (d, *J* = 8.32 Hz, 2H, Ar H), 9.39 (s, 1H, CH pyrazole), 10.54 (s, 1H, NH, D_2_O exchangeable). ^13^C NMR (100 MHz, DMSO-*d*_*6*_) δ ppm: 63.1 (CH_2_), 113.3, 119.4, 119.7, 127.2, 127.8, 128.4, 129.4, 129.6, 131.8, 135.0, 139.2, 141.1, 141.8, 143.1, 154.0 (C Ar), 162.1 (COO), 166.5 (CO–NH). Anal. Calcd. for C_24_H_21_N_5_O_7_S_2_ (555.58): C, 51.89; H, 3.81; N, 12.61. Found: C, 52.17; H, 3.98; N, 12.86.

##### 2-Oxo-2-[(4-sulfamoylphenyl) amino] ethyl 1-(4-sulfamoylphenyl)-3-(p-tolyl)-1H-pyrazole-4-carboxylate (4b)

Yield: 80%, m.p. 169–172 °C. R_f_ = 0.57 (CHCl_3_, methanol). IR (KBr) υ_max_/cm^−1^: 3300, 3271, 3132 (NH_2_, NH), 3070 (CH Ar), 2931, 2877 (CH aliph.),1716, 1680 (2 C=O), 1597 (NH bending), 1527, 1504 (C=C), 1334, 1157 (SO_2_). ^1^H NMR (400 MHz, DMSO-*d*_*6*_) δ ppm: 2.36 (s, 3H, CH_3_), 4.89 (s, 2H, CH_2_), 7.27 (d, *J* = 7.88 Hz, 4H, Ar H), 7.48 (s, 2H, NH_2_, D_2_O exchangeable), 7.74–7.80 (m, 4H, Ar H), 7.95 (s, 2H, NH_2_, D_2_O exchangeable), 7.99 (d, *J* = 8.68 Hz, 2H, Ar H), 8.23 (d, *J* = 8.68 Hz, 2H, Ar H), 9.37 (s, 1H, CH pyrazole), 10.52 (s, 1H, NH, D_2_O exchangeable). ^13^C NMR (100 MHz, DMSO-*d*_*6*_) δ ppm: 21.3 (CH_3_), 63.1 (CH_2_), 113.2 (C4, pyrazole), 119.4, 119.7, 127.2, 127.8, 128.9, 129.0, 129.4, 134.9, 138.9, 139.1, 141.1, 141.7, 143.0, 154.0 (C Ar), 162.1 (COO), 166.5 (CO–NH). Anal. Calcd. for C_25_H_23_N_5_O_7_S_2_ (569.61): C, 52.72; H, 4.07; N, 12.30. Found: C, 53.01; H, 4.29; N, 12.47.

##### 2-Oxo-2-[(4-sulfamoylphenyl) amino] ethyl 3-(4-methoxyphenyl)-1-(4-sulfamoylphenyl)-1H-pyrazole-4-carboxylate (4c)

Yield: 88%, m.p. 138–140 °C. R_f_ = 0.51 (CHCl_3_, methanol). IR (KBr) υ_max_/cm^−1^: 3325, 3263, 3109 (NH_2_, NH), 3070 (CH Ar), 2939, 2839 (CH aliph.),1720, 1701 (2 C=O), 1593 (NH bending), 1527, 1500 (C=C), 1330, 1157 (SO_2_). ^1^H NMR (400 MHz, DMSO-*d*_*6*_) δ ppm: 3.80 (s, 3H, OCH_3_), 4.89 (s, 2H, CH_2_), 7.01 (d, *J* = 8.80 Hz, 2H, Ar H), 7.27 (s, 2H, NH_2_, D_2_O exchangeable), 7.47 (s, 2H, NH_2_, D_2_O exchangeable), 7.76–7.80 (m, 4H, Ar H), 7.83 (d, *J* = 8.68 Hz, 2H, Ar H), 7.98 (d, *J* = 8.72 Hz, 2H, Ar H), 8.23 (d, *J* = 8.72 Hz, 2H, Ar H), 9.35 (s, 1H, CH pyrazole), 10.53 (s, 1H, NH, D_2_O exchangeable). ^13^C NMR (100 MHz, DMSO-*d*_*6*_) δ ppm: 55.6 (OCH_3_), 63.1 (CH_2_), 113.0, 113.8, 119.4, 119.6, 124.1, 127.2, 127.8, 130.9, 134.9, 139.1, 141.1, 141.7, 142.9, 153.8, 160.3 (C Ar), 162.2 (COO), 166.5 (CO–NH). Anal. Calcd. for C_25_H_23_N_5_O_8_S_2_ (585.61): C, 51.28; H, 3.96; N, 11.96. Found: C, 51.55; H, 3.85; N, 12.07.

##### 2-Oxo-2-[(4-sulfamoylphenyl) amino] ethyl 3-(4-bromophenyl)-1-(4-sulfamoylphenyl)-1H-pyrazole-4-carboxylate (4d)

Yield: 75%, m.p. 178–180 °C. R_f_ = 0.75 (CHCl_3_, methanol). IR (KBr) υ_max_/cm^−1^: 3329, 3259, 3113 (NH_2_, NH), 3074 (CH Ar), 1720, 1681 (2 C=O), 1597 (NH bending), 1531, 1504 (C=C), 1334, 1157 (SO_2_). ^1^H NMR (400 MHz, DMSO-*d*_*6*_) δ ppm: 4.90 (s, 2H, CH_2_), 7.28 (s, 2H, NH_2_, D_2_O exchangeable), 7.49 (s, 2H, NH_2_, D_2_O exchangeable), 7.67 (d, *J* = 8.40 Hz, 2H, Ar H), 7.74–7.80 (m, 4H, Ar H), 7.84 (d, *J* = 8.48 Hz, 2H, Ar H), 7.99 (d, *J* = 8.72 Hz, 2H, Ar H), 8.24 (d, *J* = 8.72 Hz, 2H, Ar H), 9.41 (s, 1H, CH pyrazole), 10.55 (s, 1H, NH, D_2_O exchangeable). ^13^C NMR (100 MHz, DMSO-*d*_*6*_) δ ppm: 63.2 (CH_2_), 113.3, 119.4, 119.8, 123.0, 127.2, 127.8, 131.0, 131.4, 131.6, 135.2, 139.2, 141.0, 141.7, 143.2, 152.8 (C Ar), 162.0 (COO), 166.4 (CO–NH). Anal. Calcd. for C_24_H_20_BrN_5_O_7_S_2_ (634.48): C, 45.43; H, 3.18; N, 11.04. Found: C, 45.70; H, 3.40; N, 11.27.

##### 2-Oxo-2-[(4-sulfamoylphenyl) amino] ethyl 3-(4-(methylsulfonyl) phenyl)-1-(4-sulfamoylphenyl)-1H-pyrazole-4-carboxylate (4e)

Yield: 80%, m.p. 132–135 °C. R_f_ = 0.59 (CHCl_3_, methanol). IR (KBr) υ_max_/cm^−1^: 3300, 3263, 3180 (NH_2_, NH), 3066 (CH Ar), 2927, 2808 (CH aliph.), 1715, 1698 (2 C=O), 1597 (NH bending), 1535, 1500 (C=C), 1311, 1153 (SO_2_). ^1^H NMR (400 MHz, DMSO-*d*_*6*_) δ ppm: 3.26 (s, 3H, CH_3_), 4.91 (s, 2H, CH_2_), 7.28 (s, 2H, NH_2_, D_2_O exchangeable), 7.50 (s, 2H, NH_2_, D_2_O exchangeable), 7.73–7.80 (m, 4H, Ar H), 7.95–8.03 (m, 4H, Ar H), 8.14 (d, *J* = 8.28 Hz, 2H, Ar H), 8.25 (d, *J* = 8.52 Hz, 2H, Ar H), 9.45 (s, 1H, CH pyrazole), 10.56 (s, 1H, NH, D_2_O exchangeable). ^13^C NMR (100 MHz, DMSO-*d*_*6*_) δ ppm: 43.9 (CH_3_SO_2_), 63.2 (CH_2_), 113.7, 119.4, 119.9, 127.1, 127.2, 127.8, 130.5, 135.3, 136.7, 139.1, 141.0, 141.3, 141.7, 143.3, 152.4 (C Ar), 161.9 (COO), 166.4 (CO–NH). MS, *m/Z* (%): 633.7 (M^+^), 17.34%. Anal. Calcd. for C_25_H_23_N_5_O_9_S_3_ (633.67): C, 47.39; H, 3.66; N, 11.05. Found: C, 47.61; H, 3.80; N, 11.31.

##### 2-[(4-(Ethoxycarbonyl) phenyl) amino]-2-oxoethyl 3-phenyl-1-(4-sulfamoylphenyl)-1H-pyrazole-4-carboxylate (5a)

Yield: 80%, m.p. 128–131 °C. R_f_ = 0.35 (CHCl_3_, methanol). IR (KBr) υ_max_/cm^−1^: 3332, 3259, 3132 (NH_2_, NH), 3066 (CH Ar), 2939, 2904 (CH aliph.), br. 1701 (3 C=O), 1600 (NH bending), 1535, 1504 (C=C), 1338, 1280 (SO_2_). ^1^H NMR (400 MHz, DMSO-*d*_*6*_) δ ppm: 1.31 (t, *J* = 6.92 Hz, 3H, CH_3_CH_2_), 4.28 (q, *J* = 7.08 Hz, 2H, CH_3_CH_2_), 4.89 (s, 2H, CH_2_), 7.45–7.48 (m, 4H, Ar H and NH_2_, D_2_O exchangeable), 7.74 (d, *J* = 8.36 Hz, 2H, Ar H), 7.86 (d, 2H, *J* = 8.36 Hz, Ar H), 7.94 (d, *J* = 8.48 Hz, 3H, Ar H), 7.99 (d, *J* = 8.44 Hz, 2H, Ar H), 8.24 (d, *J* = 8.32 Hz, 2H, Ar H), 9.39 (s, 1H, CH pyrazole), 10.53 (s, 1H, NH, D_2_O exchangeable). ^13^C NMR (100 MHz, DMSO-*d*_*6*_) δ ppm: 14.6 (CH_3_CH_2_), 60.9 (CH_3_CH_2_), 63.1 (CH_2_), 113.2, 119.1, 119.7, 125.0, 127.8, 128.4, 130.7, 131.8, 135.0, 141.1, 143.0, 143.1, 154.0 (C Ar), 162.1, 165.7 (2 COO), 166.5 (CO–NH). Anal. Calcd. for C_27_H_24_N_4_O_7_S (548.57): C, 59.12; H, 4.41; N, 10.21. Found: C, 59.39; H, 4.52; N, 10.47.

##### 2-[(4-(Ethoxycarbonyl) phenyl) amino]-2-oxoethyl 1-(4-sulfamoylphenyl)-3-(p-tolyl)-1H-pyrazole-4-carboxylate (5b)

Yield: 85%, m.p. 125–128 °C. R_f_ = 0.55 (CHCl_3_, methanol). IR (KBr) υ_max_/cm^−1^: 3332, 3271, 3124 (NH_2_, NH), 3070 (CH Ar), 2939, 2904 (CH aliph.), br. 1701 (3 C=O), 1600 (NH bending), 1535, 1504 (C=C), 1311, 1161 (SO_2_). ^1^H NMR (400 MHz, DMSO-*d*_*6*_) δ ppm: 1.31 (t, *J* = 7.08 Hz, 3H, CH_3_CH_2_), 2.35 (s, 3H, CH_3_), 4.29 (q, *J* = 7.08 Hz, 2H, CH_3_CH_2_), 4.88 (s, 2H, CH_2_), 7.26 (d, *J* = 7.96 Hz, 2H, Ar H), 7.48 (s, 2H, NH_2_, D_2_O exchangeable), 7.43–7.78 (m, 4H, Ar H), 7.94 (d, *J* = 8.76 Hz, 2H, Ar H), 7.99 (d, *J* = 8.72 Hz, 2H, Ar H), 8.23 (d, *J* = 8.76 Hz, 2H, Ar H), 9.37 (s, 1H, CH pyrazole), 10.53 (s, 1H, NH, D_2_O exchangeable). ^13^C NMR (100 MHz, DMSO-*d*_*6*_) δ ppm: 14.6 (CH_3_CH_2_), 21.3 (CH_3_), 60.9 (CH_3_CH_2_), 63.1 (CH_2_), 113.2, 119.1, 119.7, 125.0, 127.8, 129.0, 129.4, 130.7, 134.9, 138.9, 141.1, 143.2, 154.0 (C Ar), 162.1, 165.7 (2 COO), 166.5 (CO–NH). Anal. Calcd. for C_28_H_26_N_4_O_7_S (562.60): C, 59.78; H, 4.66; N, 9.96. Found: C, 60.04; H, 4.79; N, 9.84.

##### 2-[(4-(Ethoxycarbonyl) phenyl) amino]-2-oxoethyl 3-(4-methoxyphenyl)-1-(4-sulfamoylphenyl)-1H-pyrazole-4-carboxylate (5c)

Yield: 88%, m.p. 121–124 °C. R_f_ = 0.30 (CHCl_3_, methanol). IR (KBr) υ_max_/cm^−1^: 3321, 3294, 3120 (NH_2_, NH), 3070 (CH Ar), 2939, 2904 (CH aliph.), br. 1701 (3 C=O), 1600 (NH bending), 1527, 1503 (C=C), 1311, 1161 (SO_2_). ^1^H NMR (400 MHz, DMSO-*d*_*6*_) δ ppm: 1.31 (t, *J* = 7.32 Hz, 3H, CH_3_CH_2_), 3.80 (s, 3H, OCH_3_), 4.28 (q, *J* = 7.96 Hz, 2H, CH_3_CH_2_), 4.89 (s, 2H, CH_2_), 7.01 (d, *J* = 8.32 Hz, 2H, Ar H), 7.48 (s, 2H, NH_2_, D_2_O exchangeable), 7.74 (d, *J* = 8.80 Hz, 2H, Ar H), 7.84 (d, *J* = 8.32 Hz, 2H, Ar H), 7.93–8.00 (m, 4H, Ar H), 8.23 (d, *J* = 8.40 Hz, 2H, Ar H), 9.35 (s, 1H, CH pyrazole), 10.54 (s, 1H, NH, D_2_O exchangeable). ^13^C NMR (100 MHz, DMSO-*d*_*6*_) δ ppm: 14.6 (CH_3_CH_2_), 55.6 (OCH_3_), 60.9 (CH_3_CH_2_), 63.1 (CH_2_), 113.8, 119.1, 119.6, 124.1, 125.0, 127.8, 130.5, 130.7, 130.9, 134.9, 141.1, 142.9, 143.2, 153.8, 160.3 (C Ar), 162.2, 165.7 (2 COO), 166.5 (CO–NH). Anal. Calcd. for C_28_H_26_N_4_O_8_S (578.60): C, 58.12; H, 4.53; N, 9.68. Found: C, 58.43; H, 4.70; N, 9.85.

##### 2-[(4-(Ethoxycarbonyl) phenyl) amino]-2-oxoethyl 3-(4-bromophenyl)-1-(4-sulfamoylphenyl)-1H-pyrazole-4-carboxylate (5d)

Yield: 80%, m.p. 96–99 °C. R_f_ = 0.70 (CHCl_3_, methanol). IR (KBr) υ_max_/cm^−1^: 3310, 3263, 3190 (NH_2_, NH), 3070 (CH Ar), 2935, 2904 (CH aliph.), br. 1697 (3 C=O), 1597 (NH bending), 1531, 1504 (C=C), 1311, 1161 (SO_2_). ^1^H NMR (400 MHz, DMSO-*d*_*6*_) δ ppm: 1.31 (t, *J* = 7.04 Hz, 3H, CH_3_CH_2_), 4.28 (q, *J* = 7.21 Hz, 2H, CH_3_CH_2_), 4.90 (s, 2H, CH_2_), 7.50 (s, 2H, NH_2_, D_2_O exchangeable), 7.66 (d, *J* = 8.32 Hz, 2H, Ar H), 7.74 (d, *J* = 8.56 Hz, 2H, Ar H), 7.93–8.01 (m, 4H, Ar H), 8.19 (d, *J* = 8.44 Hz, 2H, Ar H), 8.24 (d, *J* = 8.40 Hz, 2H, Ar H), 9.40 (s, 1H, CH pyrazole), 10.55 (s, 1H, NH, D_2_O exchangeable). ^13^C NMR (100 MHz, DMSO-*d*_*6*_) δ ppm: 14.6 (CH_3_CH_2_), 60.9 (CH_3_CH_2_), 63.2 (CH_2_), 113.3, 119.1, 119.8, 122.9, 125.0, 127.8, 130.7, 131.3, 131.4, 131.6, 134.8, 135.2, 141.0, 141.2, 143.2, 152.8 (C Ar), 162.0, 165.7 (2 COO), 166.4 (CO–NH). Anal. Calcd. for C_27_H_23_BrN_4_O_7_S (627.47): C, 51.68; H, 3.69; N, 8.93. Found: C, 51.82; H, 3.85; N, 9.17.

##### 2-[(4-(Ethoxycarbonyl) phenyl) amino]-2-oxoethyl 3-(4-(methylsulfonyl) phenyl)-1-(4-sulfamoylphenyl)-1H-pyrazole-4-carboxylate (5e)

Yield: 82%, m.p. 127–130 °C. R_f_ = 0.41 (CHCl_3_, methanol). IR (KBr) υ_max_/cm^−1^: 3329, 3271, 3120 (NH_2_, NH), 3070 (CH Ar), 2927, 2873 (CH aliph.), br. 1712 (3 C=O), 1600 (NH bending), 1531, 1504 (C=C), 1311, 1149 (SO_2_). ^1^H NMR (400 MHz, DMSO-*d*_*6*_) δ ppm: 1.30 (t, *J* = 8.00 Hz, 3H, CH_3_CH_2_), 3.27 (s, 3H, CH_3_), 4.27–4.28 (m, *J* = 8.44 Hz, 2H, CH_3_CH_2_), 4.92 (s, 2H, CH_2_), 7.50 (s, 2H, NH_2_, D_2_O exchangeable), 7.74 (d, *J* = 8.16 Hz, 2H, Ar H), 7.93–8.01 (m, 6H, Ar H), 8.14 (d, *J* = 8.00 Hz, 2H, Ar H), 8.26 (d, *J* = 8.48 Hz, 2H, Ar H), 9.46 (s, 1H, CH pyrazole), 10.61 (s, 1H, NH, D_2_O exchangeable). ^13^C NMR (100 MHz, DMSO-*d*_*6*_) δ ppm: 14.6 (CH_3_CH_2_), 43.9 (CH_3_SO_2_), 60.9 (CH_3_CH_2_), 63.3 (CH_2_), 113.7, 119.1, 119.9, 125.0, 127.1, 127.8, 130.4, 130.7, 135.3, 136.7, 141.0, 141.3, 143.2, 143.3, 152.3 (C Ar), 161.9, 165.7 (2 COO), 166.4 (CO–NH). MS, *m/Z* (%): 626.1 (M^+^), 11.57%. Anal. Calcd. for C_28_H_26_N_4_O_9_S_2_ (626.66): Calculated: C, 53.67; H, 4.18; N, 8.94. Found: C, 53.81; H, 4.40; N, 9.15.

##### 2-Oxo-2-[(5-sulfamoyl-1,3,4-thiadiazol-2-yl) amino] ethyl 3-phenyl-1-(4-sulfamoylphenyl)-1H-pyrazole-4-carboxylate (6a)

Yield: 70%, m.p. 135–137 °C. R_f_ = 0.53 (CHCl_3_, methanol). IR (KBr) υ_max_/cm^−1^: 3310, 3248, 3140 (NH_2_, NH), 3066 (CH Ar), 2935, 2820 (CH aliph.), 1708, 1639 (2 C=O), 1597 (NH bending), 1531, 1508 (C=C), 1311, 1161 (SO_2_). ^1^H NMR (400 MHz, DMSO-*d*_*6*_) δ ppm: 4.97 (s, 2H, CH_2_), 7.43 (s, 4H, 2 NH_2_, D_2_O exchangeable), 7.91 (d, *J* = 7.56 Hz, 2H, Ar H), 7.96–8.00 (m, 3H, Ar H), 8.11 (d, *J* = 8.68 Hz, 2H, Ar H), 8.24 (d, *J* = 8.56 Hz, 2H, Ar H), 8.67 (s, 1H, CH pyrazole), 9.38 (s, 1H, NH, D_2_O exchangeable). ^13^C NMR (100 MHz, DMSO-*d*_*6*_) δ ppm: 52.8 (CH_2_), 118.5, 119.7, 127.8, 128.4, 129.1, 129.6, 131.7, 134.9, 138.8, 141.8, 143.0, 152.7, 155.5 (C Ar), 167.5 (COO), 173.8 (CO–NH). Anal. Calcd. for C_20_H_17_N_7_O_7_S_3_ (563.58): C, 42.62; H, 3.04; N, 17.07. Found: C, 42.93; H, 3.21; N, 17.28.

##### 2-Oxo-2-[(5-sulfamoyl-1,3,4-thiadiazol-2-yl) amino] ethyl 1-(4-sulfamoylphenyl)-3-(p-tolyl)-1H-pyrazole-4-carboxylate (6b)

Yield: 80%, m.p. 115–120 °C. R_f_ = 0.78 (CHCl_3_, methanol). IR (KBr) υ_max_/cm^−1^: 3310, 3251, 3136 (NH_2_, NH), 3070 (CH Ar), 2924, 2850 (CH aliph.), 1712, 1651 (2 C=O), 1597 (NH bending), 1527, 1504 (C=C), 1311, 1161 (SO_2_). ^1^H NMR (400 MHz, DMSO-*d*_*6*_) δ ppm: 2.37 (s, 3H, CH_3_), 5.05 (s, 2H, CH_2_), 7.26 (d, *J* = 7.96 Hz, 2H, Ar H), 7.47 (s, 4H, 2 NH_2_, D_2_O exchangeable), 7.74 (d, *J* = 7.32 Hz, 2H, Ar H), 7.97 (d, *J* = 9.04 Hz, 2H, Ar H), 8.18 (d, *J* = 8.20 Hz, 2H, Ar H), 9.17 (s, 1H, CH pyrazole), 9.36 (s, 1H, NH, D_2_O exchangeable). ^13^C NMR (100 MHz, DMSO-*d*_*6*_) δ ppm: 21.3 (CH_3_), 62.8 (CH_2_), 119.5, 119.7, 127.8, 128.9, 129.0, 129.4, 134.5, 135.0, 138.6, 141.3, 142.7, 153.9, 154.1 (C Ar), 164.1 (COO), 168.2 (CO–NH). Anal. Calcd. for C_21_H_19_N_7_O_7_S_3_ (577.61): C, 43.67; H, 4.32; N, 16.98. Found: C, 43.93; H, 4.50; N, 16.84.

##### 2-Oxo-2-[(5-sulfamoyl-1,3,4-thiadiazol-2-yl) amino] ethyl 3-(4-methoxyphenyl)-1-(4-sulfamoylphenyl)-1H-pyrazole-4-carboxylate (6c)

Yield: 75%, m.p. 240–241 °C. R_f_ = 0.40 (CHCl_3_, methanol). IR (KBr) υ_max_/cm^−1^: 3371, 3251, 3140 (NH_2_, NH), 3074 (CH Ar), 2935, 2839 (CH aliph.), 1697, 1666 (2 C=O), 1597 (NH bending), 1527, 1508 (C=C), 1338, 1161 (SO_2_). ^1^H NMR (400 MHz, DMSO-*d*_*6*_) δ ppm: 3.82 (s, 3H, OCH_3_), 4.94 (s, 2H, CH_2_), 7.02 (d, *J* = 8.32 Hz, 2H, Ar H), 7.47 (s, 4H, 2 NH_2_, D_2_O exchangeable), 7.83 (d, *J* = 6.84 Hz, 2H, Ar H), 7.97 (d, *J* = 8.56 Hz, 2H, Ar H), 8.19 (d, *J* = 8.44 Hz, 2H, Ar H), 9.17 (s, 1H, CH pyrazole), 12.64 (s, 1H, NH, D_2_O exchangeable). ^13^C NMR (100 MHz, DMSO-*d*_*6*_) δ ppm: 55.6 (OCH_3_), 61.9 (CH_2_), 113.7, 114.7, 119.4, 124.5, 127.7, 130.9, 134.6, 141.3, 142.7, 153.6, 160.1, 162.2, 162.7, (C Ar), 164.2 (COO), 166.4 (CO–NH). MS, *m/Z* (%): 593.7 (M^+^), 42.62%. Anal. Calcd. for C_21_H_19_N_7_O_8_S_3_ (593.60): C, 42.49; H, 3.23; N, 16.52. Found: C, 42.71; H, 3.48; N, 16.70.

#### General procedure for the synthesis of compounds (9a-e)

A mixture of the acid hydrazide compounds **8a-e** (0.01 mol) and ammonium thiocyanate (0.95 g, 0.01 mol) was dissolved in ethanol (10 mL). 10% HCl (3.6 mL) was then added, and refluxing the reaction mixture for 22–25 h. The solution was poured onto ice. The resultant precipitate was filtered, dried, and recrystallized from ethanol.

##### 2-[3-Phenyl-1-(4-sulfamoylphenyl)-1H-pyrazole-4-carbonyl] hydrazine-1-carbothioamide (9a)

Yield: 85%, m.p. 180–181 °C. R_f_ = 0.34 (CHCl_3_, methanol). IR (KBr) υ_max_/cm^−1^: 3452, 3340, 3182 (NH_2_, NH), 3090 (CH Ar), 1678 (C=O), 1600 (NH bending), 1543, 1504 (C=C), 1311, 1161 (SO_2_), 1273 (C=S). ^1^H NMR (400 MHz, DMSO-*d*_*6*_) δ ppm: 7.43–7.45 (m, 3H, Ar H), 7.48 (s, 2H, NH_2_, D_2_O exchangeable), 7.59 (s, 1H, NH, D_2_O exchangeable), 7.89–7.91 (m, 2H, Ar H), 8.05 (d, *J* = 7.84 Hz, 4H, Ar H), 9.18 (s, 1H, CH pyrazole), 9.45 (s, 1H, NH, D_2_O exchangeable), 10.25 (s, 2H, NH_2_, D_2_O exchangeable). ^13^C NMR (100 MHz, DMSO-*d*_*6*_) δ ppm: 116.1, 119.0, 128.1, 128.5, 128.9, 129.1, 132.0, 141.3, 142.7, 152.6 (C Ar), 162.5 (C=O), 182.5 (C=S). MS, *m/Z* (%): 416.4 (M^+^), 13.87%. Anal. Calcd. for C_17_H_16_N_6_O_3_S_2_ (416.47): C, 49.03; H, 3.87; N, 20.18. Found: C, 49.21; H, 4.06; N, 20.41.

##### 2-[1-(4-Sulfamoylphenyl)-3-(p-tolyl)-1H-pyrazole-4-carbonyl] hydrazine-1-carbothioamide (9b)

Yield: 85%, m.p. 178–180 °C. R_f_ = 0.41 (CHCl_3_, methanol). IR (KBr) υ_max_/cm^−1^: 3429, 3305, 3182 (NH_2_, NH), 3080 (CH Ar), 2974, 2920 (CH aliph.), 1678 (C=O), 1597 (NH bending), 1539, 1504 (C=C), 1311, 1161 (SO_2_), 1273 (C=S). ^1^H NMR (400 MHz, DMSO-*d*_*6*_) δ ppm: 2.36 (s, 3H, CH_3_), 7.25 (d, *J* = 8.04 Hz, 2H, Ar H), 7.47 (s, 2H, NH_2_, D_2_O exchangeable), 7.57 (s, 1H, NH, D_2_O exchangeable), 7.80 (d, *J* = 7.84 Hz, 2H, Ar H), 8.03–8.10 (m, 4H, Ar H), 9.16 (s, 1H, CH pyrazole), 9.44 (s, 1H, NH, D_2_O exchangeable), 10.23 (s, 2H, NH_2_, D_2_O exchangeable). ^13^C NMR (100 MHz, DMSO-*d*_*6*_) δ ppm: 21.3 (CH_3_), 115.9, 118.8, 128.1, 128.7, 129.1, 129.2, 132.0, 138.6, 141.4, 142.6, 152.6 (C Ar), 162.6 (C=O), 182.5 (C=S). Anal. Calcd. for C_18_H_18_N_6_O_3_S_2_ (430.50): C, 50.22; H, 4.21; N, 19.52. Found: C, 50.46; H, 4.37; N, 19.80.

##### 2-[3-(4-Methoxyphenyl)-1-(4-sulfamoylphenyl)-1H-pyrazole-4-carbonyl] hydrazine-1-carbothioamide (9c)

Yield: 90%, m.p. 205–207 °C. R_f_ = 0.37 (CHCl_3_, methanol). IR (KBr) υ_max_/cm^−1^: 3433, 3332, 3248 (NH_2_, NH), 3090 (CH Ar), 2974, 2839 (CH aliph.), 1670 (C=O), 1604 (NH bending), 1550, 1504 (C=C), 1338, 1161 (SO_2_), 1280 (C=S). ^1^H NMR (400 MHz, DMSO-*d*_*6*_) δ ppm: 3.81 (s, 3H, OCH_3_), 7.00 (d, *J* = 6.88 Hz, 2H, Ar H), 7.47 (s, 2H, NH_2_, D_2_O exchangeable), 7.58 (s, 1H, NH, D_2_O exchangeable), 7.87 (d, *J* = 8.64 Hz, 2H, Ar H), 8.02–8.10 (m, 4H, Ar H), 9.15 (s, 1H, CH pyrazole), 9.43 (s, 1H, NH, D_2_O exchangeable), 10.22 (s, 2H, NH_2_, D_2_O exchangeable). ^13^C NMR (100 MHz, DMSO-*d*_*6*_) δ ppm: 55.6 (OCH_3_), 113.9, 115.7, 118.8, 124.4, 128.1, 130.2, 132.0, 141.4, 142.5, 152.4, 160.1 (C Ar), 162.7 (C=O), 182.5 (C=S). Anal. Calcd. for C_18_H_18_N_6_O_4_S_2_ (446.50): C, 48.42; H, 4.06; N, 18.82. Found: C, 48.68; H, 4.20; N, 19.03.

##### 2-[3-(4-Bromophenyl)-1-(4-sulfamoylphenyl)-1H-pyrazole-4-carbonyl] hydrazine-1-carbothioamide (9d)

Yield: 85%, m.p. 238–241 °C. R_f_ = 0.52 (CHCl_3_, methanol). IR (KBr) υ_max_/cm^−1^: 3348, 3271, 3186 (NH_2_, NH), 3062 (CH Ar), 1662 (C=O), 1597 (NH bending), 1527, 1496 (C=C), 1330, 1157 (SO_2_), 1284 (C=S). ^1^H NMR (400 MHz, DMSO-*d*_*6*_) δ ppm: 7.48 (s, 2H, NH_2_, D_2_O exchangeable), 7.65 (d, *J* = 8.56 Hz, 2H, Ar H), 7.88 (d, *J* = 8.40 Hz, 3H, Ar H and NH, D_2_O exchangeable), 8.03–8.10 (m, 4H, Ar H), 9.17 (s, 1H, CH pyrazole), 9.44 (s, 1H, NH, D_2_O exchangeable), 10.28 (s, 2H, NH_2_, D_2_O exchangeable). ^13^C NMR (100 MHz, DMSO-*d*_*6*_) δ ppm: 116.1, 119.1, 122.6, 128.1, 130.9, 131.3, 131.5, 132.3, 141.2, 142.8, 151.5 (C Ar), 162.3 (C=O), 182.6 (C=S). Anal. Calcd. for C_17_H_15_BrN_6_O_3_S_2_ (495.37): C, 41.22; H, 3.05; N, 16.97. Found: C, 41.39; H, 3.24; N, 17.15.

##### 2-[3-(4-(Methylsulfonyl) phenyl)-1-(4-sulfamoylphenyl)-1H-pyrazole-4-carbonyl] hydrazine-1-carbothioamide (9e)

Yield: 90%, m.p. 261–262 °C. R_f_ = 0.10 (CHCl_3_, methanol). IR (KBr) υ_max_/cm^−1^: 3309, 3236 (NH_2_, NH), 3016 (CH Ar), 2989, 2904 (CH aliph.), 1701 (C=O), 1612 (NH bending), 1597, 1508 (C=C), 1307, 1153 (SO_2_), 1284 (C=S). ^1^H NMR (400 MHz, DMSO-*d*_*6*_) δ ppm: 3.28 (s, 3H, CH_3_), 7.50 (s, 2H, NH_2_, D_2_O exchangeable), 7.65 (s, 1H, NH, D_2_O exchangeable), 8.00 (d, *J* = 8.48 Hz, 2H, Ar H), 8.06–8.10 (m, 4H, Ar H), 8.17 (d, *J* = 8.20 Hz, 2H, Ar H), 9.21 (s, 1H, CH pyrazole), 9.46 (s, 1H, NH, D_2_O exchangeable), 10.34 (s, 2H, NH_2_, D_2_O exchangeable). ^13^C NMR (100 MHz, DMSO-*d*_*6*_) δ ppm: 43.9 (CH_3_SO_2_), 116.6, 119.3, 127.2, 128.1, 129.7, 132.4, 136.9, 141.0, 141.2, 143.1, 151.0 (C Ar), 162.2 (C=O), 182.6 (C=S). Anal. Calcd. for C_18_H_18_N_6_O_5_S_3_ (494.56): C, 43.72; H, 3.67; N, 16.99. Found: C, 43.90; H, 3.81; N, 17.15.

#### General procedure for the synthesis of compound (10)

A solution of the acylthiosemicarbazide compound **9b** (2.1 g, 0.005 mol) and sulfuric acid (10 mL) was heated in a water bath at 90 °C for 8 h. After that, the reaction mixture was added to ice-water and neutralized with ammonium hydroxide while cooling. After being filtered, dried, and crystalized from ethanol, the precipitate was obtained.

##### 4-[4-(5-Amino-1,3,4-thiadiazol-2-yl)-3-(p-tolyl)-1H-pyrazol-1-yl] benzenesulfonamide (10)

Yield: 85%, m.p. 230–234 °C. R_f_ = 0.35 (CHCl_3_, methanol). IR (KBr) υ_max_/cm^−1^: 3271, 3194 (NH_2_), 3062 (CH Ar), 2850, 2773 (CH aliph.), 1635 (NH bending), 1593, 1516 (C=C), 1315, 1161 (SO_2_). ^1^H NMR (400 MHz, DMSO-*d*_*6*_) δ ppm: 2.38 (s, 3H, CH_3_), 7.30 (d, *J* = 7.92 Hz, 2H, Ar H), 7.46 (s, 4H, 2 NH_2_, D_2_O exchangeable), 7.60 (d, *J* = 8.20 Hz, 2H, Ar H), 7.98 (d, *J* = 8.80 Hz, 2H, Ar H), 8.17 (d, *J* = 8.80 Hz, 2H, Ar H), 9.20 (s, 1H, CH pyrazole). ^13^C NMR (100 MHz, DMSO-*d*_*6*_) δ ppm: 21.3 (CH_3_), 113.0, 119.3, 127.8, 129.3, 129.5, 130.6, 139.2, 141.3, 142.7, 148.3, 151.6, 164.0, 169.2 (C Ar). MS, *m/Z* (%): 412.2 (M^+^), 10.97%. Anal. Calcd. for C_18_H_16_N_6_O_2_S_2_ (412.49): C, 52.41; H, 3.91; N, 20.37. Found: C, 52.69; H, 4.07; N, 20.52.

#### General procedure for the synthesis of compound (11)

To a solution of acylthiosemicarbazide compound **9b** (2.1 g, 0.005 mol) in ethanol, sodium hydroxide 1% (15 mL) was added. Then, the reaction mixture was refluxed for 6 h. Then, acidify the reaction mixture with HCl (10%) while cooling. The resultant precipitate was filtered, dried, and crystallized from ethanol.

##### 4-[4-(5-Mercapto-1H-1,2,4-triazol-3-yl)-3-(p-tolyl)-1H-pyrazol-1-yl] benzenesulfonamide (11)

Yield: 80%, m.p. 255–257 °C. R_f_ = 0.40 (CHCl_3_, methanol). IR (KBr) υ_max_/cm^−1^: 3421, 3229 br. (NH_2_, NH), 3070 (CH Ar), 2920, 2881 (CH aliph.), 1631 (NH bending), 1597, 1512 (C=C), 1315, 1161 (SO_2_), 1226 (C=S). ^1^H NMR (400 MHz, DMSO-*d*_*6*_) δ ppm: 2.37 (s, 3H, CH_3_), 7.28 (d, *J* = 7.92 Hz, 2H, Ar H), 7.47 (s, 2H, NH_2_, D_2_O exchangeable), 7.66 (d, *J* = 8.20 Hz, 2H, Ar H), 8.01 (d, *J* = 8.92 Hz, 2H, Ar H), 8.07 (d, *J* = 8.92 Hz, 2H, Ar H), 9.11 (s, 1H, CH pyrazole), 13.55 (s, 1H, NH, D_2_O exchangeable), 13.59 (s, 1H, SH, D_2_O exchangeable). ^13^C NMR (100 MHz, DMSO-*d*_*6*_) δ ppm: 21.3 (CH_3_), 108.5, 119.3, 128.0, 128.6, 129.0, 129.3, 131.4, 138.8, 141.3, 142.7, 144.9, 151.5 (C Ar), 166.7 (C-SH). Anal. Calcd. for C_18_H_16_N_6_O_2_S_2_ (412.49): C, 52.41; H, 3.91; N, 20.37. Found: C, 52.58; H, 4.12; N, 20.49.

### Biological assays

#### Carbonic anhydrase inhibitory activity

Biological investigation of the target compounds as hCA IX and hCA XII inhibitors was carried out at the laboratory of the Egyptian company for the production of vaccines, sera, and medications (VACSERA, Giza, Egypt), using the spectrophotometric method described by Pocker and Meany [35, 36] (Supplementary data, S.1.2).

#### Anticancer activity

The guidelines of the Drug Evaluation Branch’s protocol at the NCI in Bethesda served as the basis for the in vitro anticancer screening assays [37, 38]. at one dose 10 µM against a panel of 60 cancer cell lines. The human tumor cell lines used belonged to nine panels: leukemia, melanoma, lung, colon, CNS, ovarian, renal, prostate and breast cancers using the sulforhodamine B (SRB) assay in a 48 h drug exposure protocol [39].

#### In vitro cytotoxic activity

Compound **5b** was selected for further investigation of its in vitro cytotoxicity against MCF-7 breast cancer cell line using MTT assay [40]. Additional data were depicted in the Supplementary data, S.1.3.

#### Cell cycle analysis

Following the manufacturer’s instructions, the Annexin V-FITC Apoptosis Detection Kit (Bio Vision) was used to assess the impact of compound **5b** on the MCF-7 cell line’s cell cycle stages [41–43]. and the experimental procedure was shown in the Supplementary data, S.1.4.1.

#### Apoptotic assay

Studying the apoptotic induction liability of compound **5b** on MCF-7 cancer cell line was performed using annexin V-FITC/propidium iodide double staining protocol. [41–44] More information was presented in the Supplementary data, S.1.4.2.

### In silico studies

#### Molecular modelling

Using two X-ray crystallographic structures of CA IX (PDB ID: 5FL4) and CA XII (PDB ID: 1JD0) co-crystallized with thiophene sulfamoyl inhibitor **(9FK)** [31] and **(AAZ)** inhibitor [32], respectively, molecular docking studies were carried out using Molecular Operating Environment (MOE) software version 2022.02. The methodology is described in detail in the Supplementary data, S.6.1.

#### Toxicity prediction

Compounds were drawn using ChemDraw, then the smiles were copied into another virtual filter (Osiris Property Explorer (https://www.organic-chemistry.org/prog/peo/) [45].

### ANOVA statistical analysis and CA IC_50_ calculations

The software used to calculate CA IC_50_ along with statistical analysis of the results is GraphPad Prism 9.00. Results were estimated by using one-way ANOVA and the Bonferroni’s multiple comparisons posthoc test was performed (*P* < 0.05) [46] (Supplementary data, S4 and S5).

## Supplementary Information

Below is the link to the electronic supplementary material.

Supplementary file1 (PDF 6799 KB)

## Data Availability

No datasets were generated or analysed during the current study.
