# Supplementary material for: Insight on novel sulfamoylphenyl pyrazole derivatives as anticancer carbonic anhydrase inhibitors
Source: Mol Divers. 2024 Nov 11;29(5):4705–25. doi: 10.1007/s11030-024-11023-3 (PMC12454493; doi:10.1007/s11030-024-11023-3)
Supplement: Supplementary file 1 — Supplementary file1 (PDF 6799 KB) [file 11030_2024_11023_MOESM1_ESM.pdf]

Insight on novel sulfamoylphenyl pyrazole derivatives as anticancer carbonic anhydrase inhibitors

Rehab F. Ahmed[a*], Walaa R. Mahmoud[a], Nagwa M. Abdelgawad[a], Amany Belal[b], Reem I. Alsantali[b], Mona F. Said[a]

[a]*Pharmaceutical Chemistry Department, Faculty of Pharmacy, Cairo University, Kasr El-Aini St., Cairo 11562, Egypt.*

[b]*Department of Pharmaceutical Chemistry, College of Pharmacy, Taif University, P.O.Box 11099,Taif 21944, Saudi Arabia*

S1. The Experimental procedures

S1.1. Chemistry

All the reagents were purchased from VWR International Merck, Germany or Sigma-Aldrich. All reactions were monitored by TLC using silica gel F254 plates using Merck, Germany and the spots were observed using a UV light with a wavelength of 254nm. Melting points were determined by open capillary tube method using Electrothermal Stuart SMP3 digital melting point apparatus and they were uncorrected. By using KBr discs, Infrared spectra were determined by Shimadzu-FTIR and expressed as wave number (cm^{-1}), Microanalytical unit Faculty of Pharmacy, Cairo University. The NMR spectra were recorded on Bruker in deuterated chloroform $CDCl_3$ or deuterated dimethylsulphoxide (DMSO-*d6*) at 400 MHz for ^{1}HNMR and 100 MHz for ^{13}CMR on Bruker AVANCE III 400 MHz FT-NMR spectrometer, the Microanalytical unit, Faculty of Pharmacy, Cairo University. Chemical Shifts were quoted in δ as parts per million (ppm). As for the proton magnetic resonance, D_2O was carried out for NH and OH exchangeable proton. The elemental microanalyses were performed at the Regional Centre for Mycology and Biotechnology, Al-Azhar University. Also, mass spectra were recorded using Thermo Scientific ISOLT mass spectrometer at the Regional Center for Mycology and Biotechnology, Al-Azhar University. All compounds were chemically named using chemical name facility of ChemDraw Ultra software V 10.

S.1.2. Carbonic anhydrase inhibitory activity:

The spectrophotometric assay was conducted in HEPES-Tris buffer, NaCl of pH 7.5 (20mM) at 25◦C. Each inhibitory well-consisted of 50 μL of assay buffer solution, 20 μL of hCA enzyme solution (20 ng/μL in Assay Buffer), and 50 μL of test compound in HPLC grade DMSO (maintain 10% of the final concentration). The mixture solution was preincubated for 15 min at 25°C. Substrate p-nitrophenyl acetate (p-NPA) (0.7mM) was prepared in HPLC grade methanol and the reaction was started by adding 20 μL to a well in a 96- well-plate. The amount of product formed was measured for 30 min continuously at 1 min intervals at 400 nm in a 96-well-plate using xMARK microplate spectrophotometer, Bio-Rad (USA). The activity of the controlled compound was taken as 100%. All experiments were carried out in triplicates of each used concentration, and results are represented as a mean of the triplicate.

S.1.3. *In vitro* cytotoxic activity:

Firstly, cultures were delivered into sterile area then each vial was supplied with 3 mL of medium or balanced salt solution free from phenol red and serum. Cultures were subjected to incubation foe 2-4 g. After the incubation period, MTT solubilization solution was added to remove the produced formazan crystals. Spectrophotometric measurement of absorbance was carried out at 690 nm for the background which then subtracted from absorbance measured at 570 nm. Five different drug concentrations were used 100, 25, 6.3, 1.6 and 0.4 nM and measured in triplicate.

S.1.4. Cell cycle analysis and Apoptotic assay

S.1.4.1. Cell cycle analysis

The effect of compound **6e** on the cell cycle profile of 768-0, SF-539 and HS 578T was determined using Annexin V-FITC Apoptosis Detection Kit (Bio Vision) according to the manufacturer's procedures. Briefly, cell lines were cultured and incubated with each of the tested compounds for 48 h. Then, the cells were collected by centrifugation and resuspended in 500 µl of 1X binding buffer. 5 µl of Annexin V-FITC was added and 5 µl of propidium iodide (PI 50 mg/ml, optional.). The cells were incubated at room temperature for 5 min in the dark. Annexin V-FITC binding was analyzed by flow cytometry (Ex = 488 nm; Em = 530 nm) using FITC signal detector and PI staining was analyzed by the phycoerythrin emission signal detector.

S.1.4.2. Apoptotic assay:

cell lines were cultured and incubated with each of the tested compounds for 48 h. Then, the cells were collected by centrifugation and resuspended in 500 µl of 1X binding buffer. 5 µl of Annexin V-FITC was added and 5 µl of propidium iodide (PI 50 mg/ml, optional.). the cells were incubated at room temperature for 5 min in the dark. Annexin V-FITC binding was analyzed by flow cytometry (Ex = 488 nm; Em = 530 nm) using FITC signal detector and PI staining was analyzed by the phycoerythrin emission signal detector.

S2- ^{1}H-NMR and ^{13}C-NMR spectra

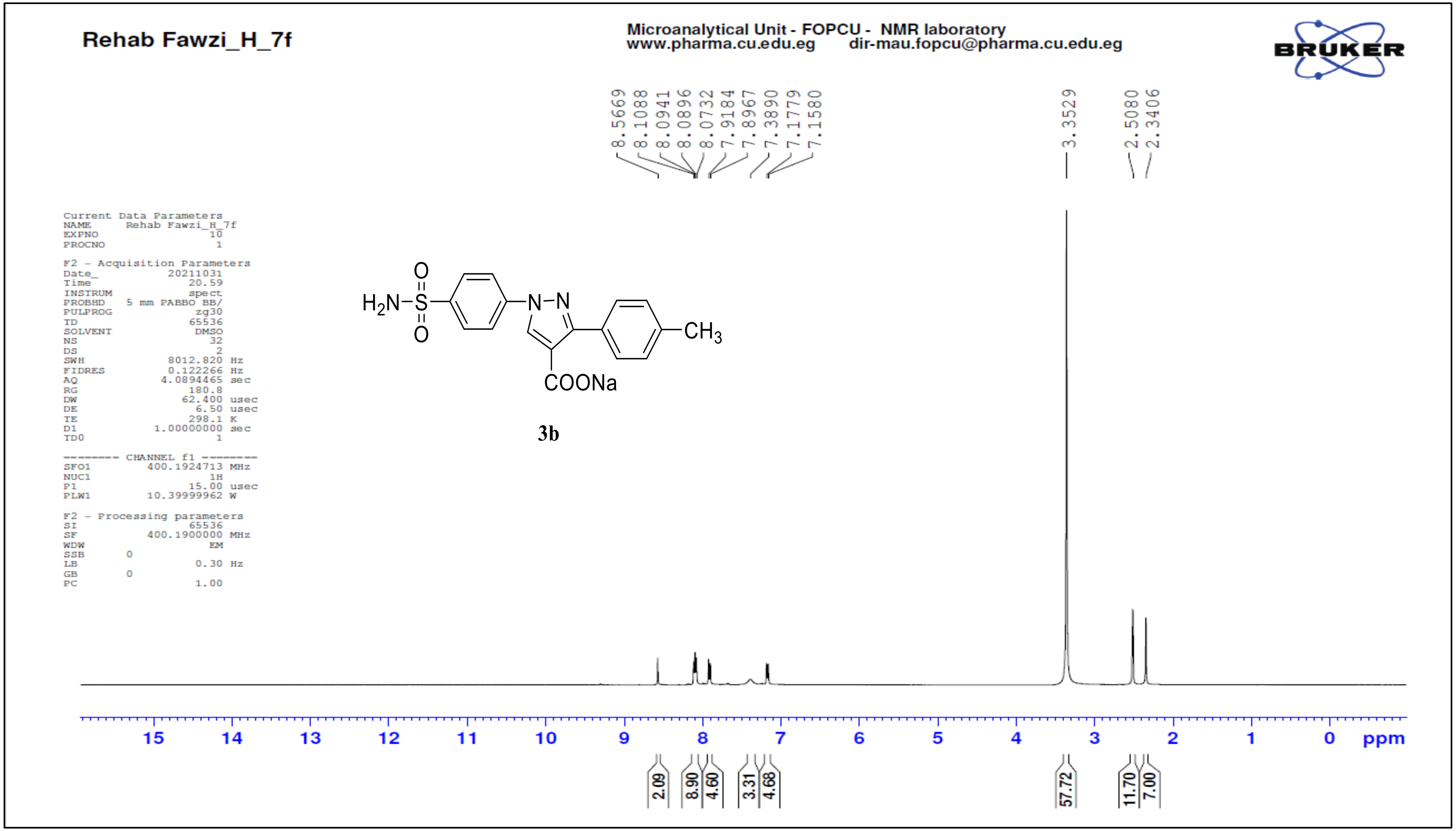

Figure S1: ^{1}H NMR spectrum of Sodium 1-(4-sulfamoylphenyl)-3-(*p*-tolyl)-1*H*-pyrazole-4-carboxylate **(3b)**.

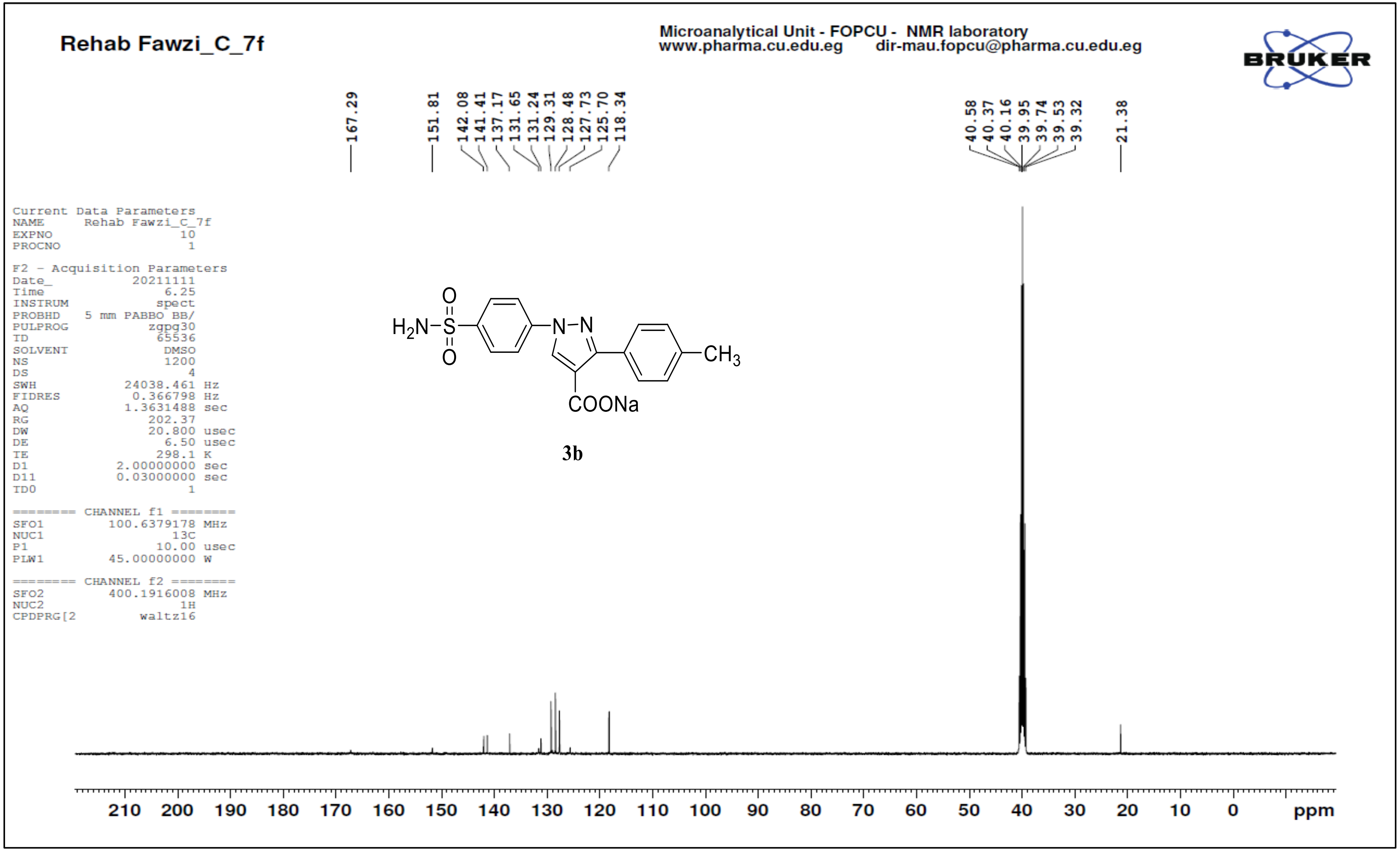

Figure S2: ^{13}C NMR spectrum of Sodium 1-(4-sulfamoylphenyl)-3-(*p*-tolyl)-1*H*-pyrazole-4-carboxylate **(3b)**.

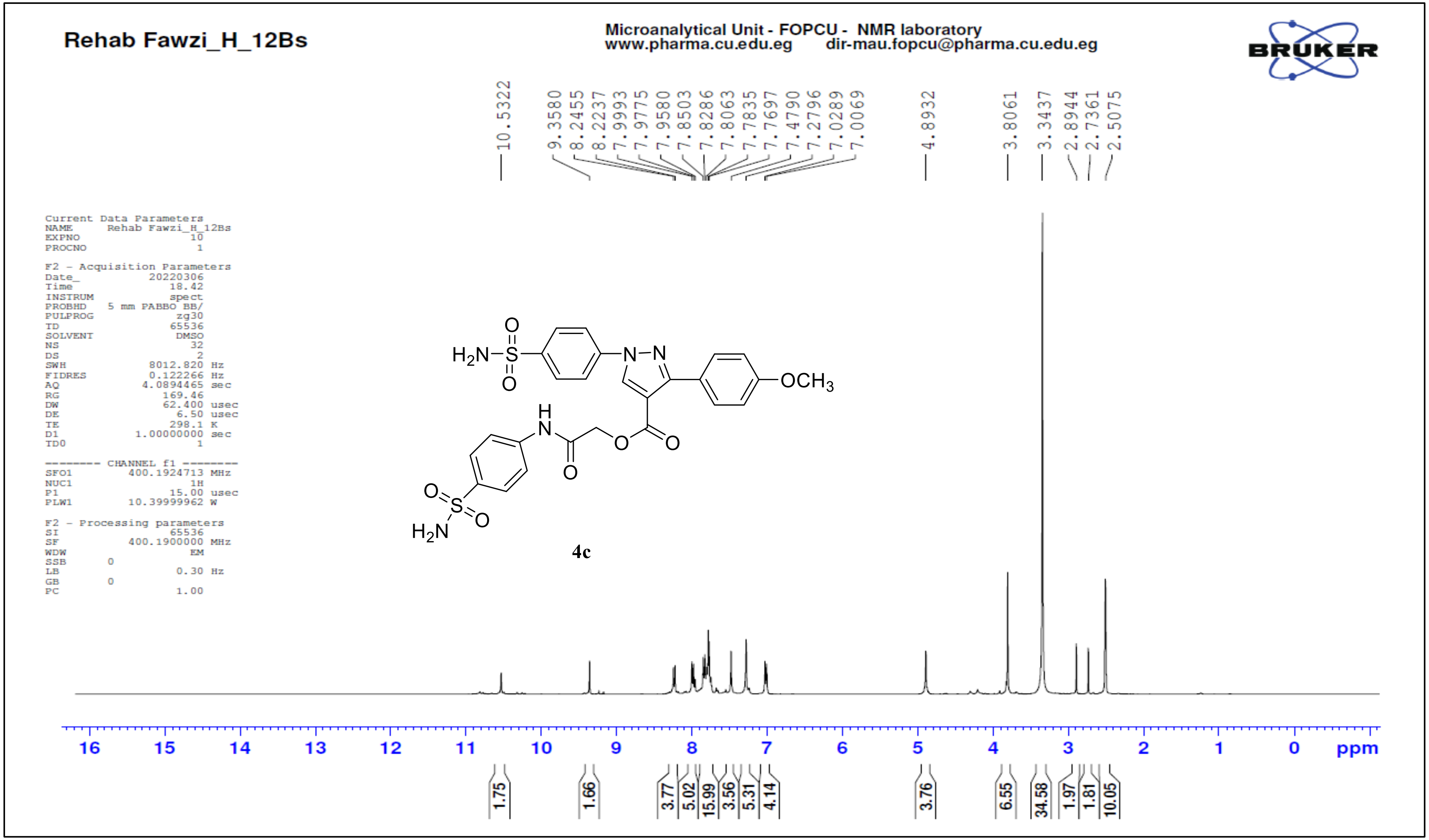

Figure S3: ^{1}H NMR spectrum of 2-Oxo-2-[(4-sulfamoylphenyl) amino] ethyl 3-(4-methoxyphenyl)-1-(4-sulfamoylphenyl)-1*H*-pyrazole-4-carboxylate **(4c)**.

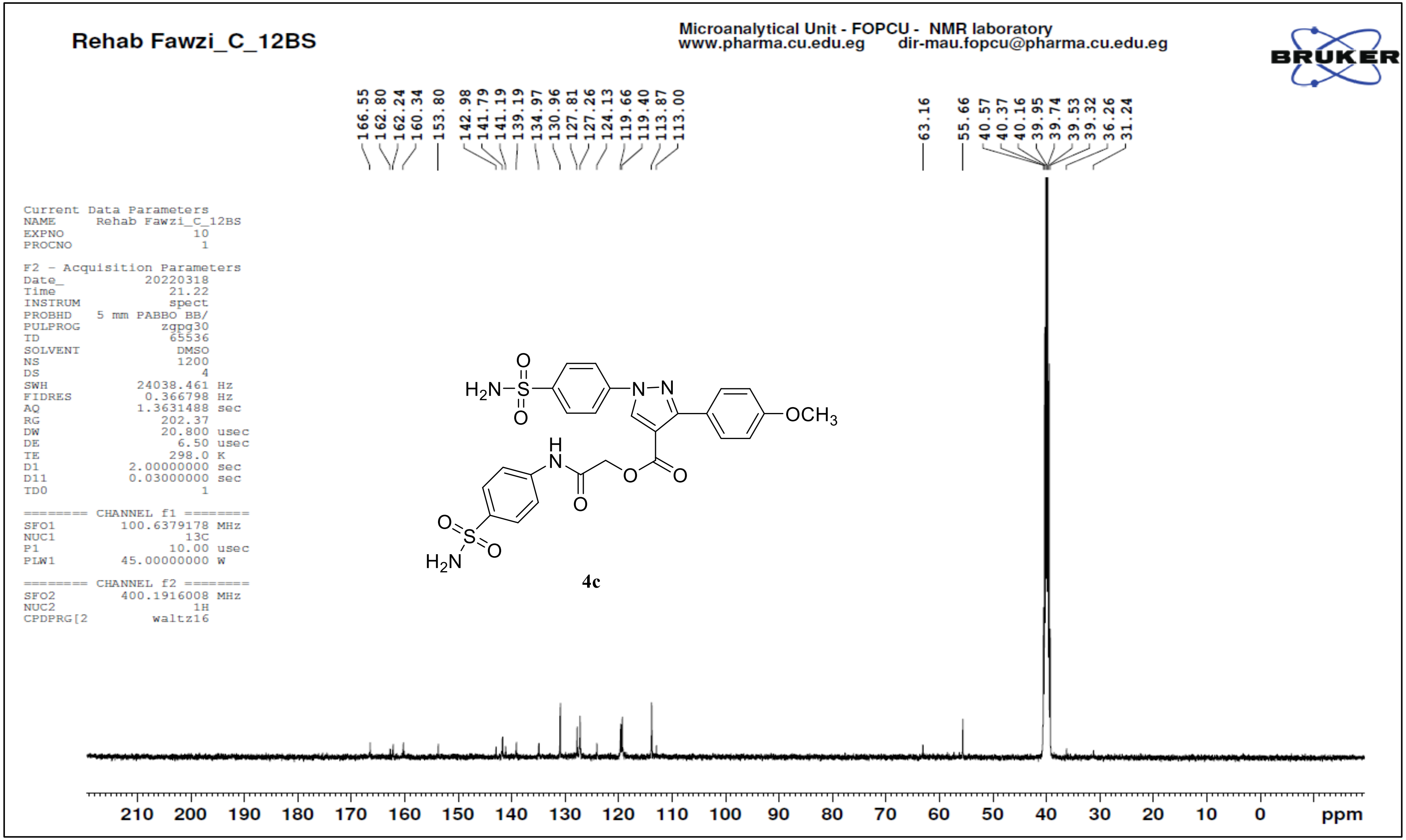

Figure S4: ^{13}C NMR spectrum of 2-Oxo-2-[(4-sulfamoylphenyl) amino] ethyl 3-(4-methoxyphenyl)-1-(4-sulfamoylphenyl)-1*H*-pyrazole-4-carboxylate **(4c)**.

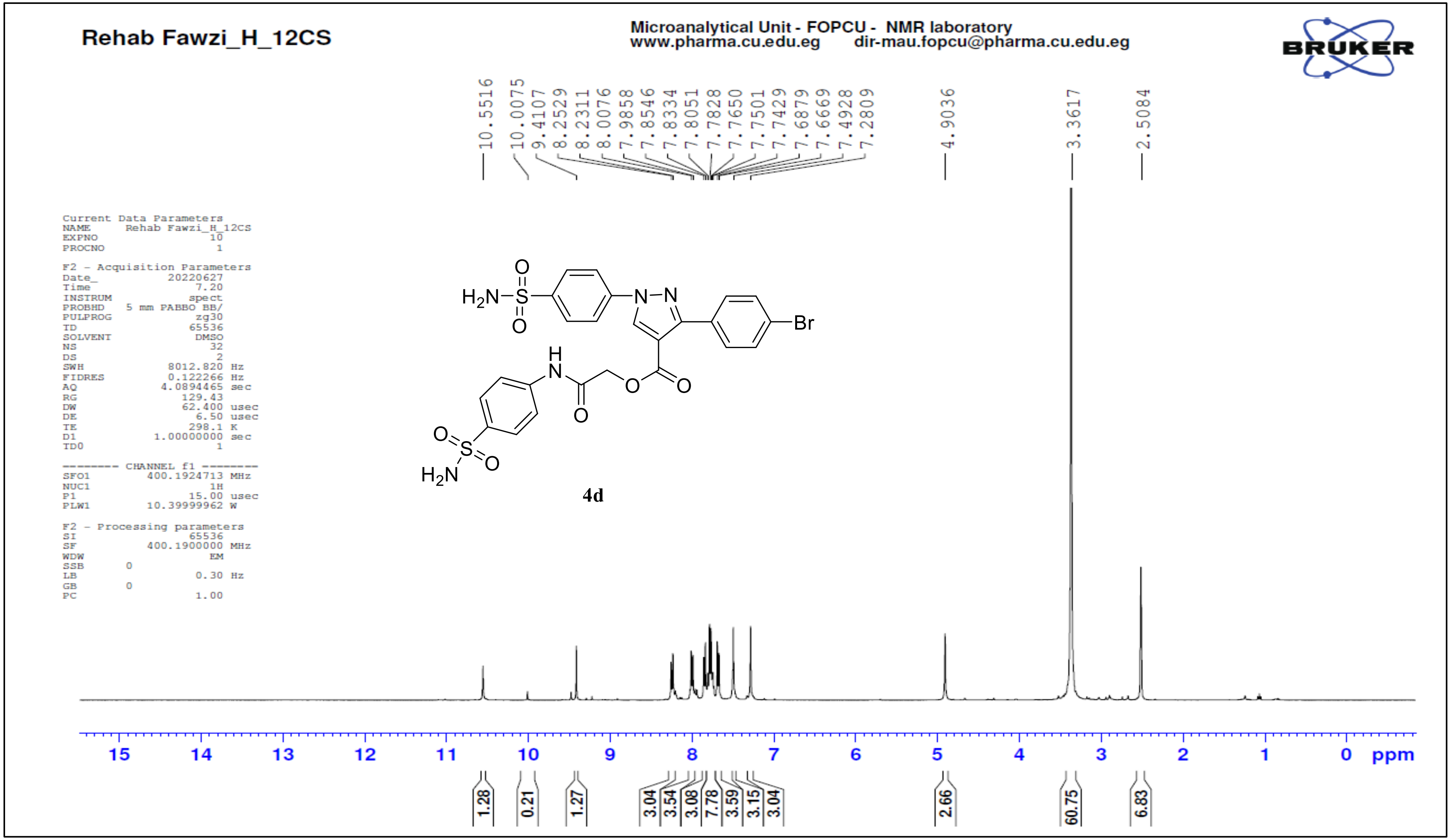

Figure S5: ^{1}H NMR spectrum of 2-Oxo-2-[(4-sulfamoylphenyl) amino] ethyl 3-(4-bromophenyl)-1-(4-sulfamoylphenyl)-1*H*-pyrazole-4-carboxylate **(4d)**.

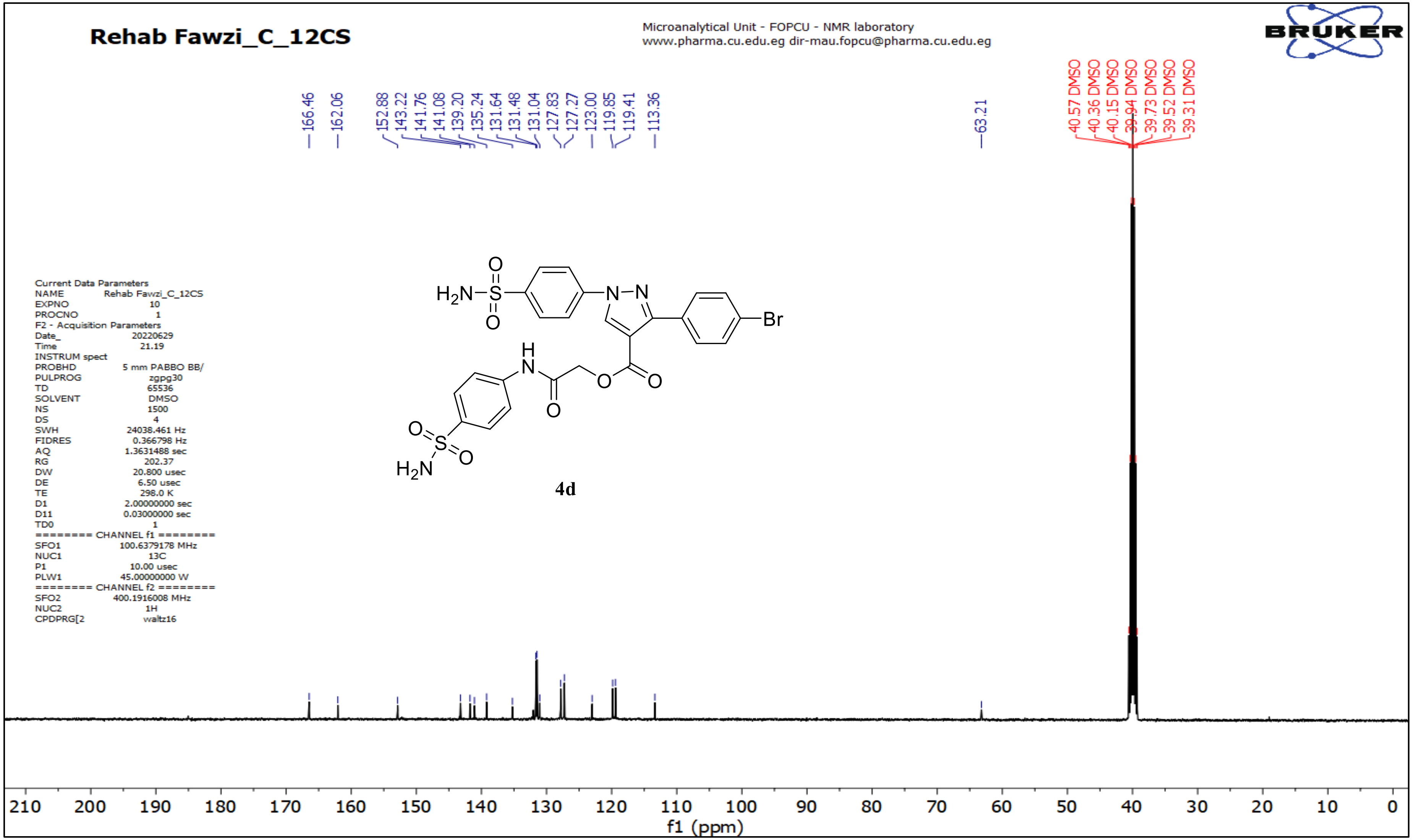

Figure S6: ^{13}C NMR spectrum of 2-Oxo-2-[(4-sulfamoylphenyl) amino] ethyl 3-(4-bromophenyl)-1-(4-sulfamoylphenyl)-1*H*-pyrazole-4-carboxylate **(4d)**.

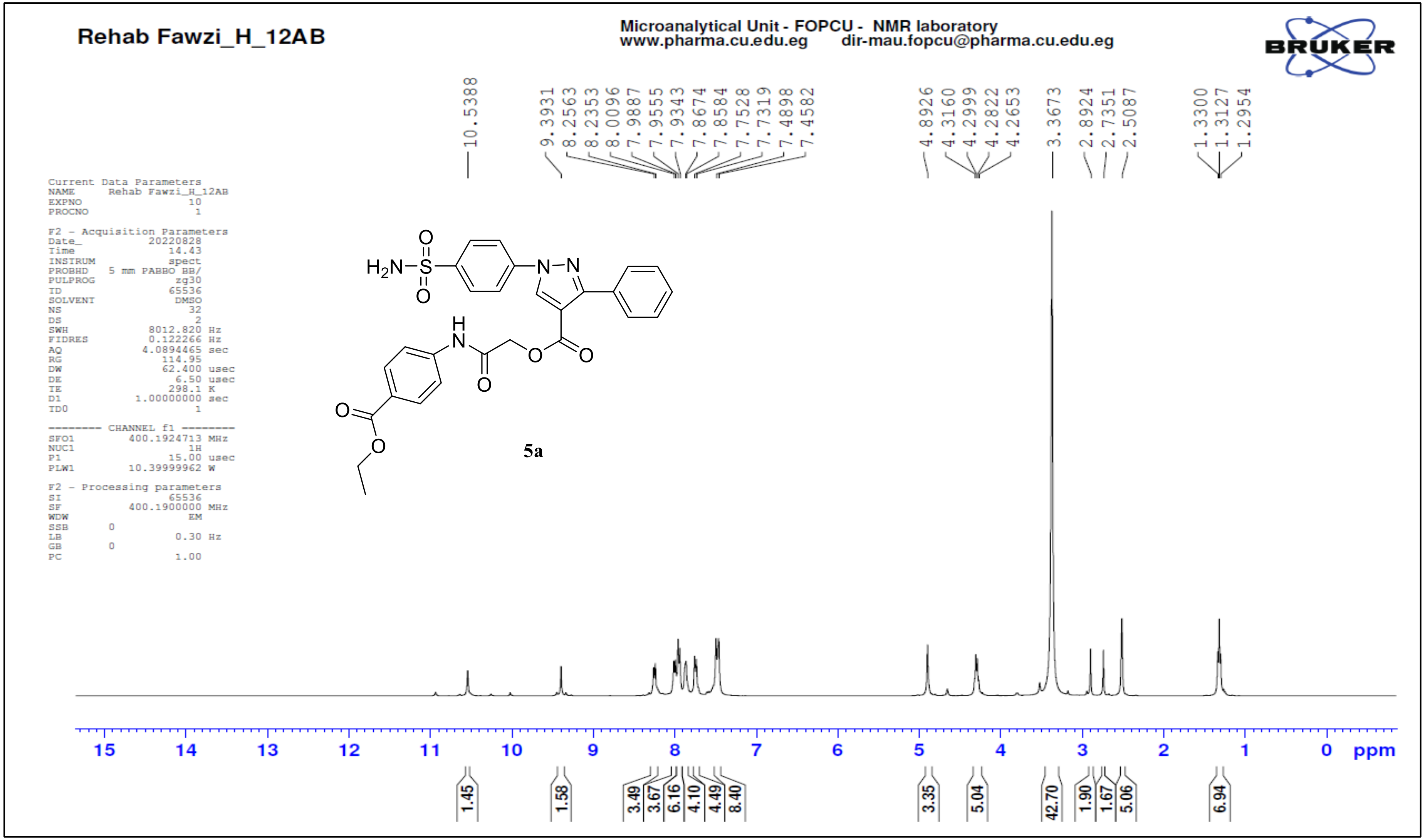

Figure S7: ^{1}H NMR spectrum of 2-[(4-(Ethoxycarbonyl) phenyl) amino]-2-oxoethyl 3-phenyl-1-(4-sulfamoylphenyl)-1*H*-pyrazole-4-carboxylate **(5a)**.

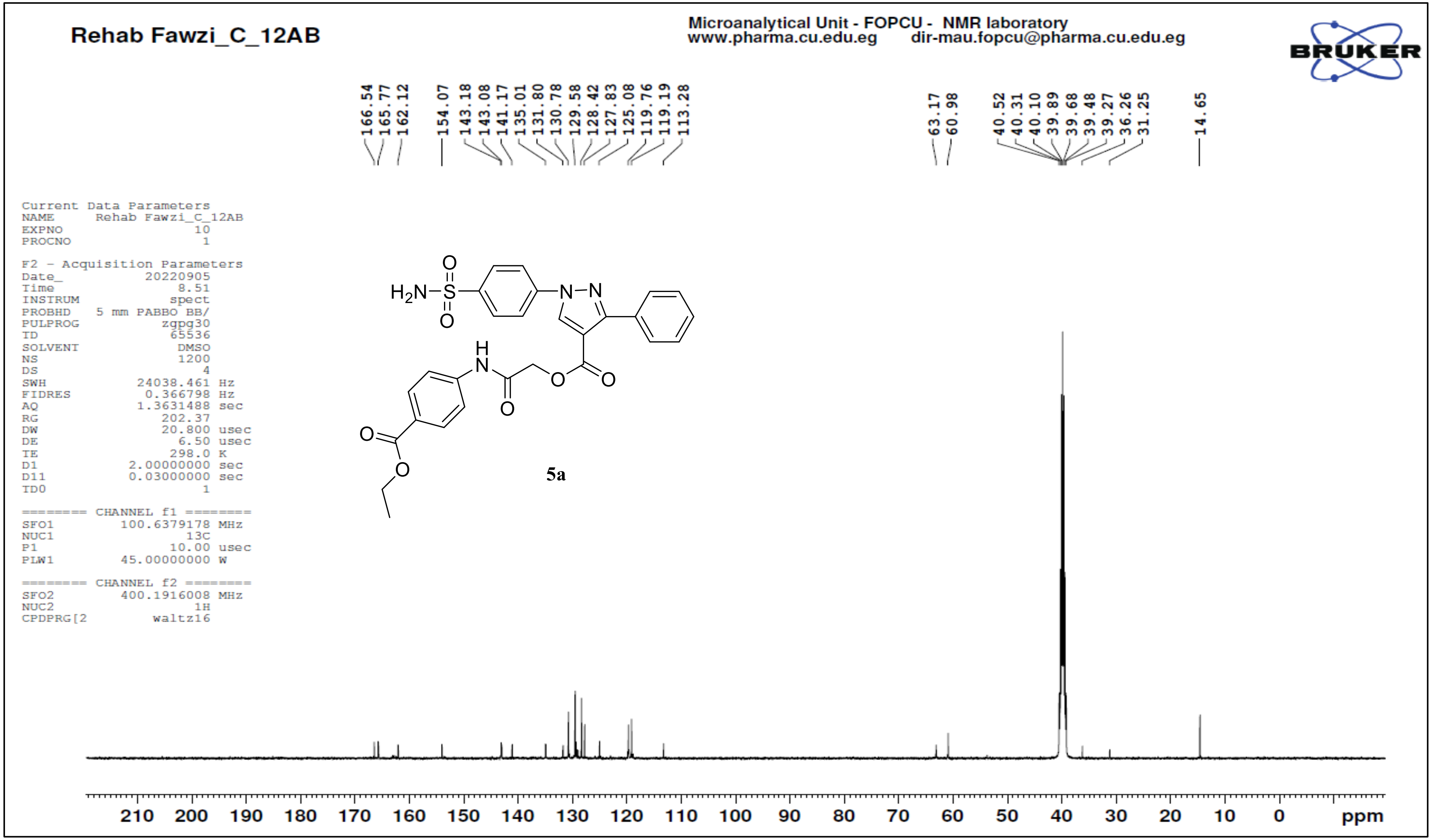

Figure S8: ^{13}C NMR spectrum of 2-[(4-(Ethoxycarbonyl) phenyl) amino]-2-oxoethyl 3-phenyl-1-(4-sulfamoylphenyl)-1*H*-pyrazole-4-carboxylate **(5a)**.

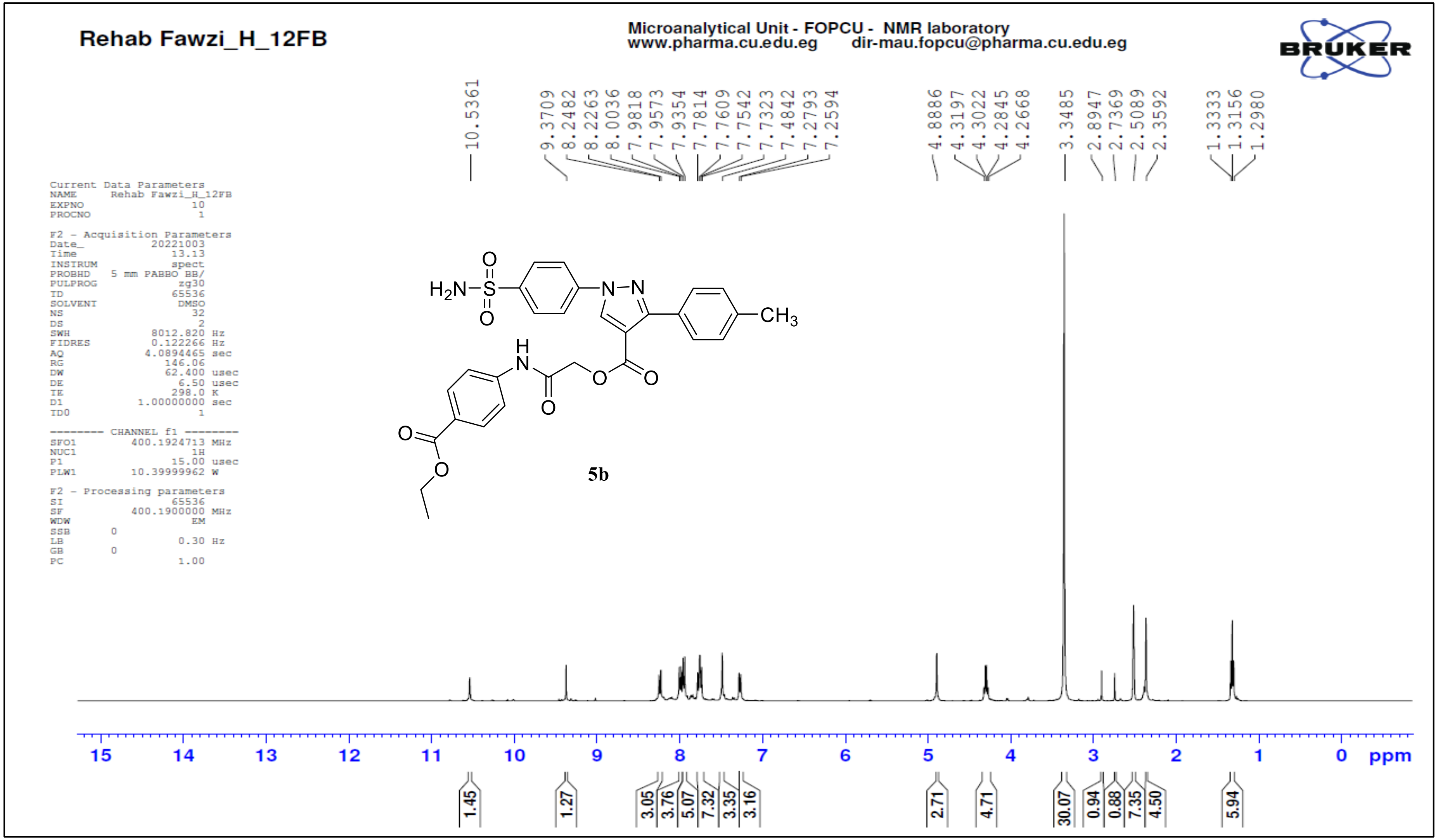

Figure S9: ^{1}H NMR spectrum of 2-[(4-(Ethoxycarbonyl) phenyl) amino]-2-oxoethyl 1-(4-sulfamoylphenyl)-3-(*p*-tolyl)-1*H*-pyrazole-4-carboxylate **(5b)**.

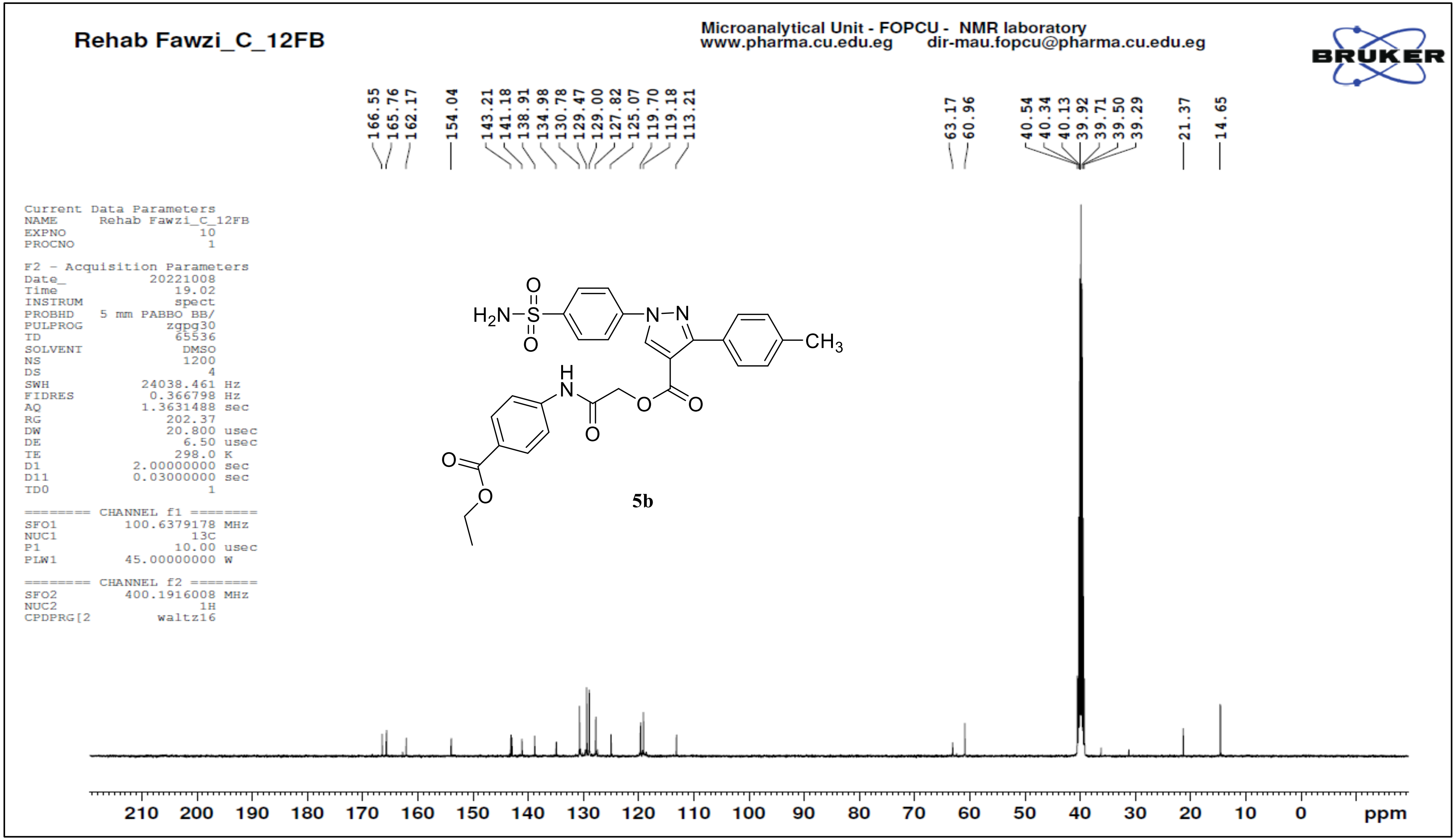

Figure S10: ^{13}C NMR spectrum of 2-[(4-(Ethoxycarbonyl) phenyl) amino]-2-oxoethyl 1-(4-sulfamoylphenyl)-3-(*p*-tolyl)-1*H*-pyrazole-4-carboxylate **(5b)**.

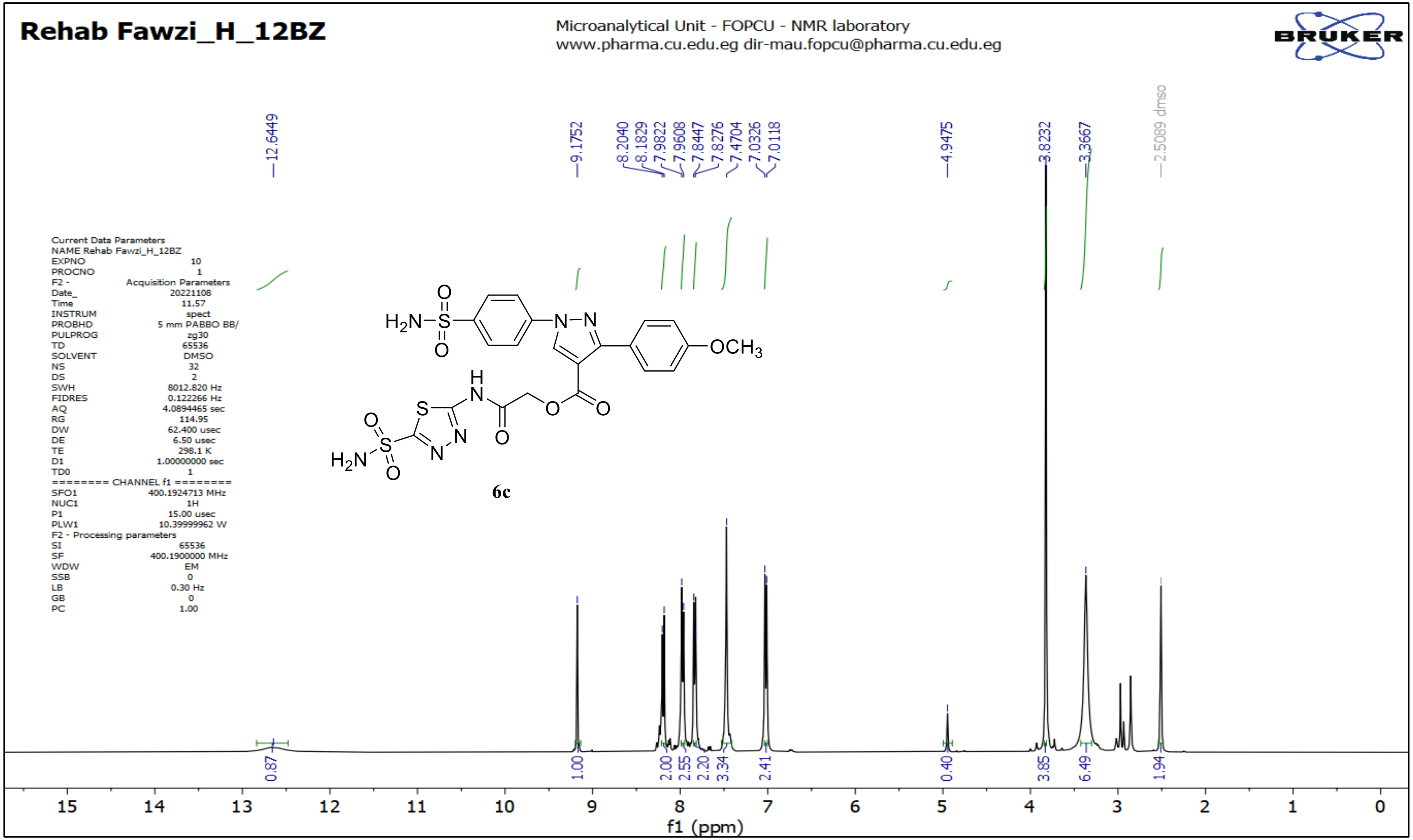

Figure S11: ^{1}H NMR spectrum of 2-Oxo-2-[(5-sulfamoyl-1,3,4-thiadiazol-2-yl) amino] ethyl 3-(4-methoxyphenyl)-1-(4-sulfamoylphenyl)-1*H*-pyrazole-4-carboxylate **(6c)**.

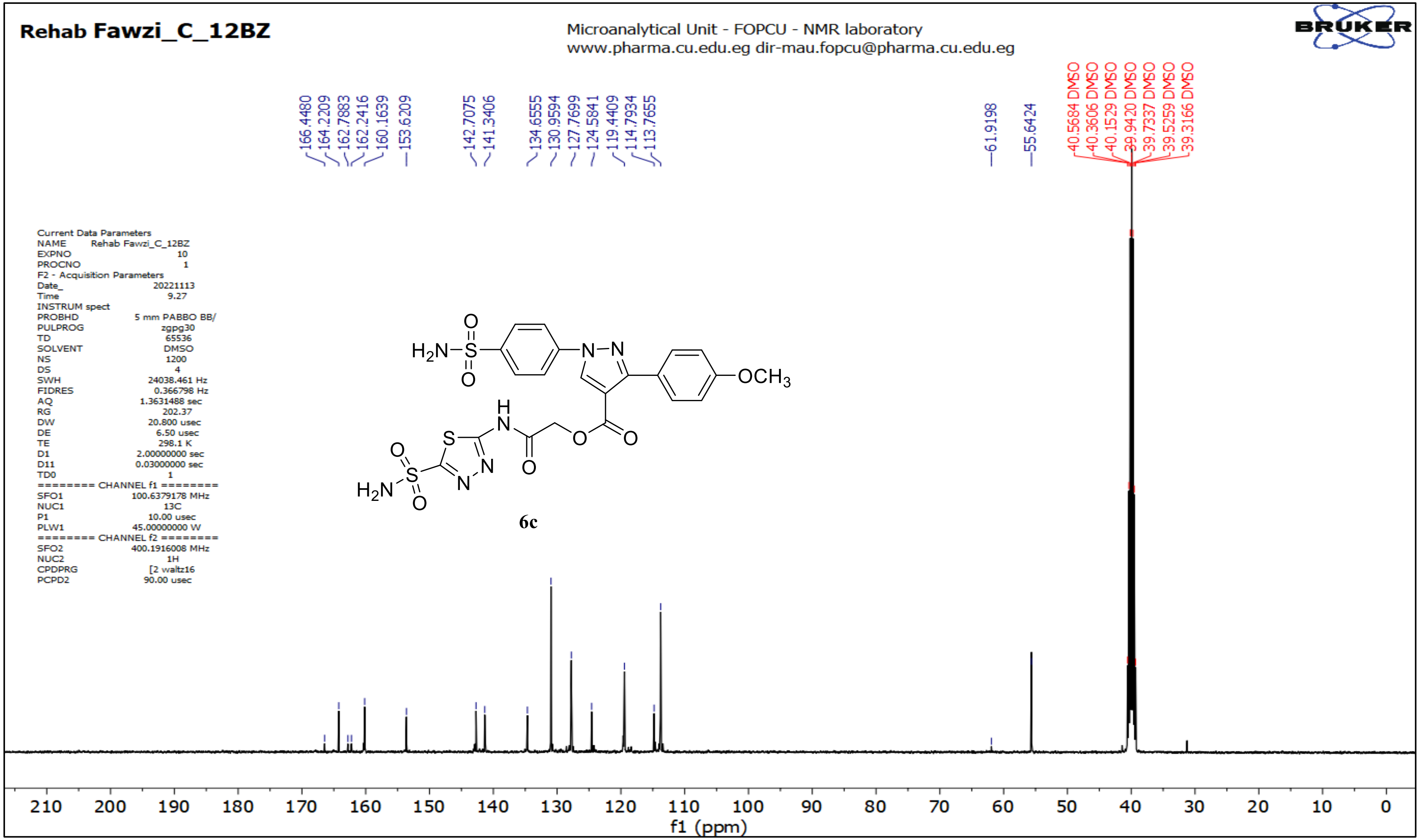

Figure S12: ^{13}C NMR spectrum of 2-Oxo-2-[(5-sulfamoyl-1,3,4-thiadiazol-2-yl) amino] ethyl 3-(4-methoxyphenyl)-1-(4-sulfamoylphenyl)-1*H*-pyrazole-4-carboxylate **(6c)**.

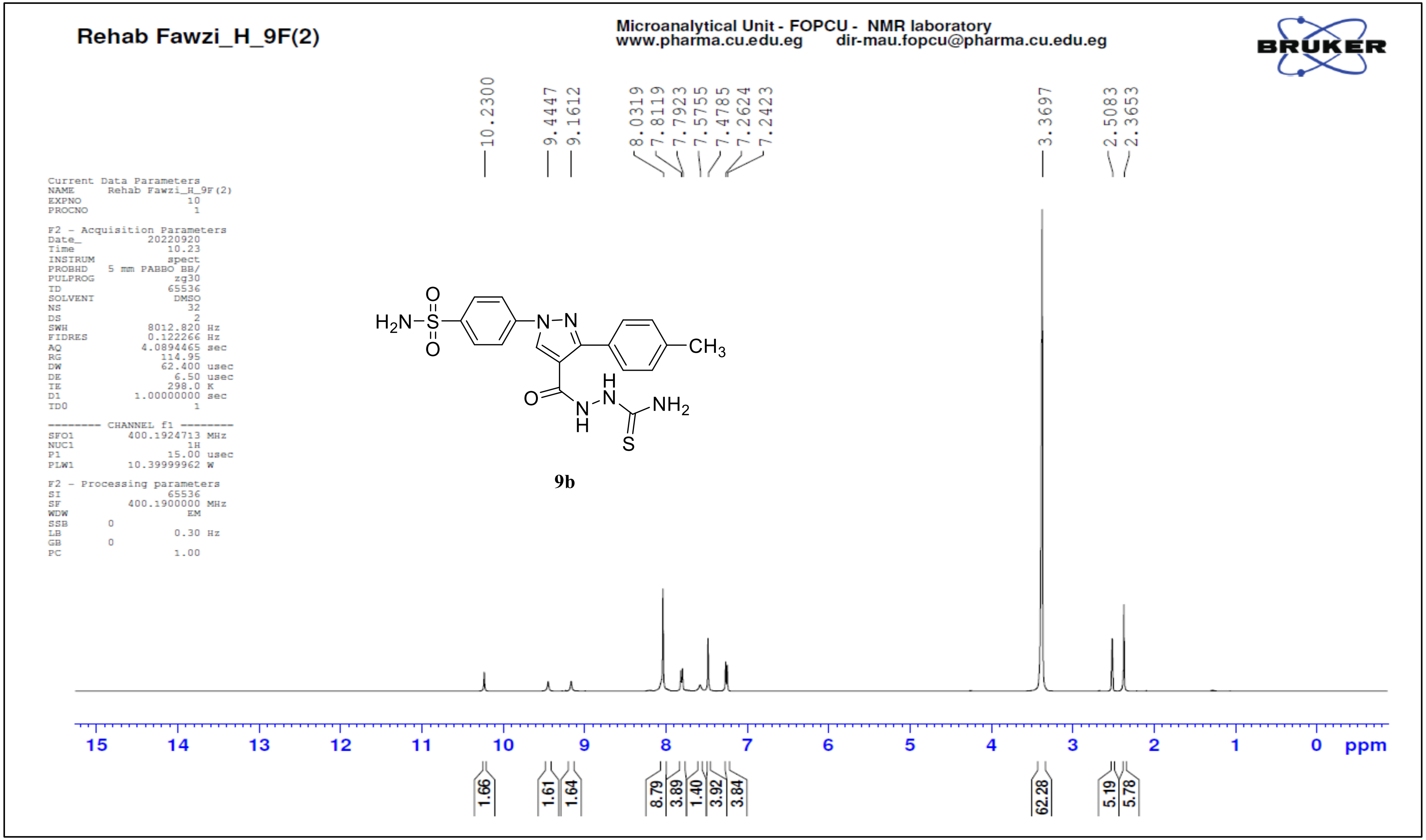

Figure S13: ^{1}H NMR spectrum of 2-[1-(4-Sulfamoylphenyl)-3-(*p*-tolyl)-1*H*-pyrazole-4-carbonyl] hydrazine-1-carbothioamide **(9b)**.

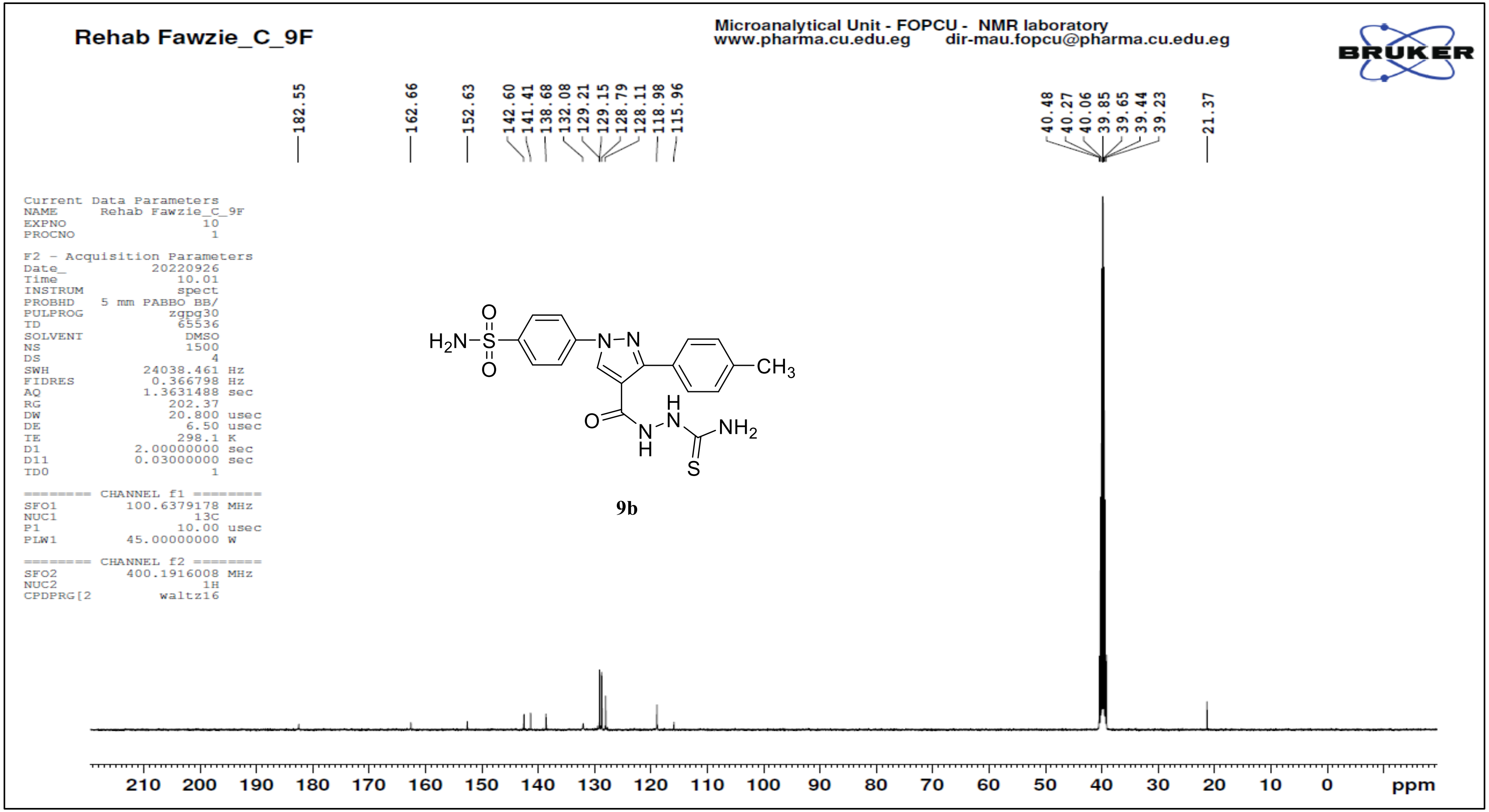

Figure S14: ^{13}C NMR spectrum of 2-[1-(4-Sulfamoylphenyl)-3-(*p*-tolyl)-1*H*-pyrazole-4-carbonyl] hydrazine-1-carbothioamide **(9b)**.

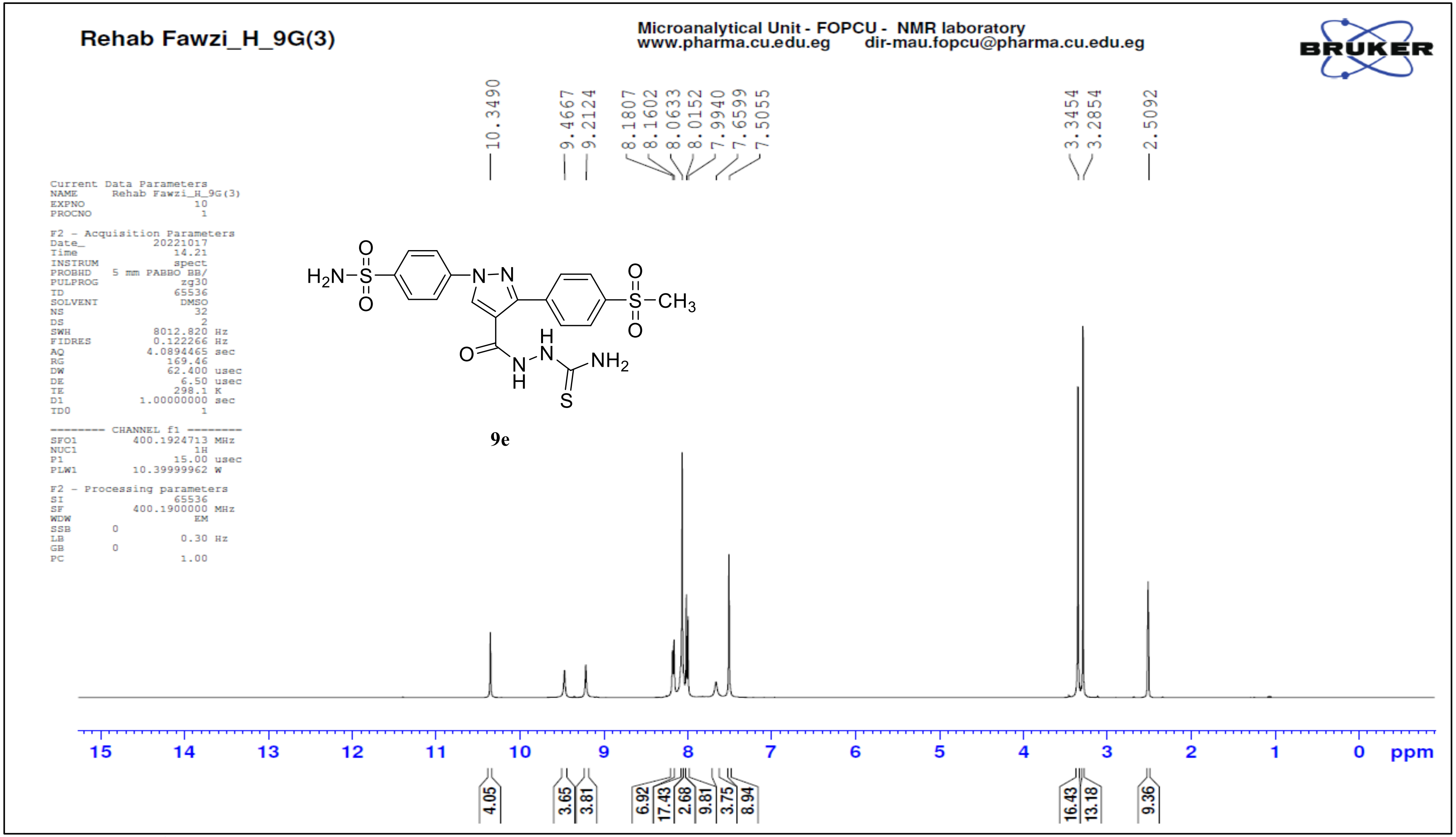

Figure S15: ^{1}H NMR spectrum of 2-[3-(4-(Methylsulfonyl) phenyl)-1-(4-sulfamoylphenyl)-1*H*-pyrazole-4-carbonyl] hydrazine-1-carbothioamide **(9e)**.

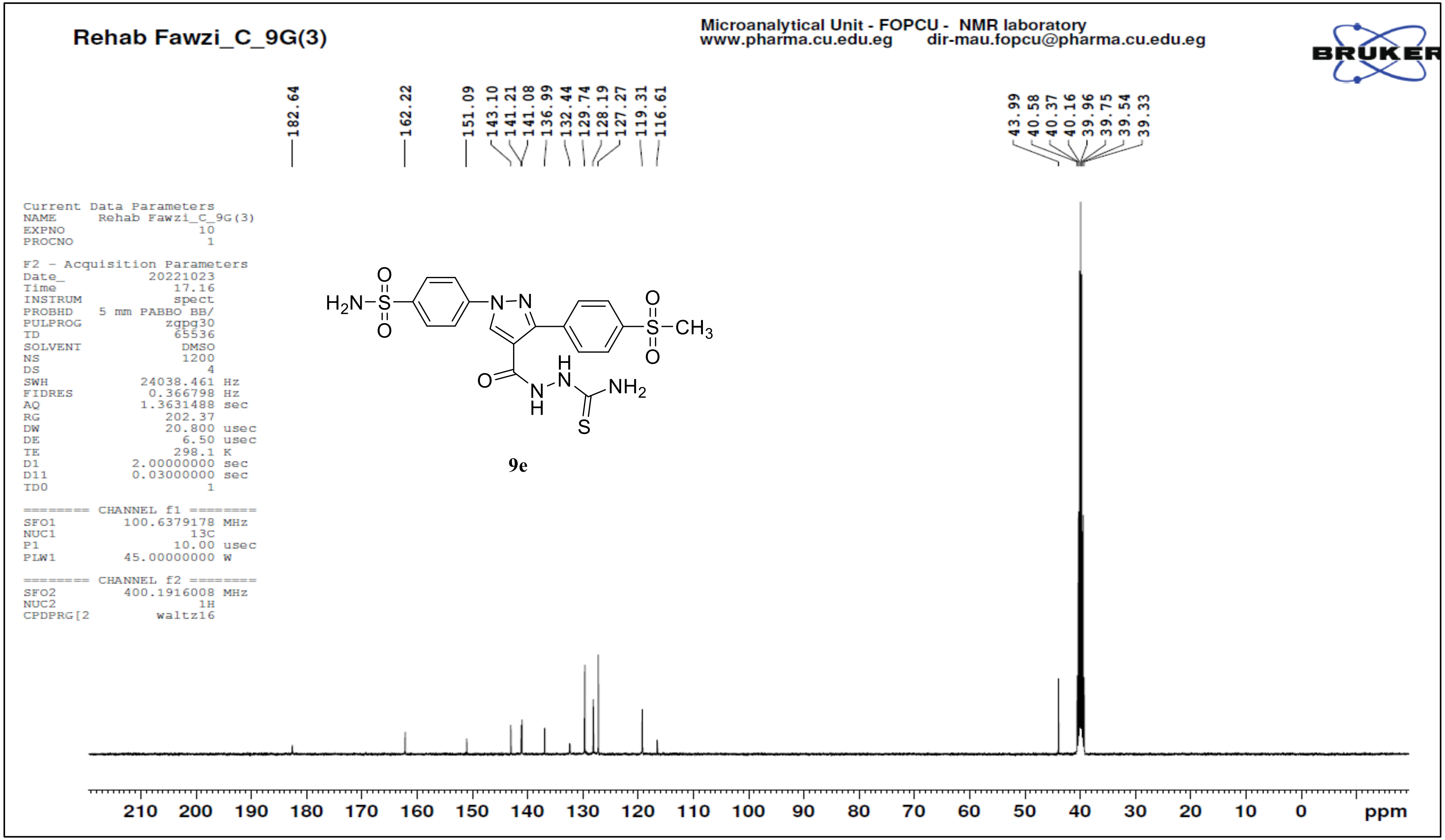

Figure S16: ^{13}C NMR spectrum of 2-[3-(4-(Methylsulfonyl) phenyl)-1-(4-sulfamoylphenyl)-1*H*-pyrazole-4-carbonyl] hydrazine-1-carbothioamide **(9e)**.

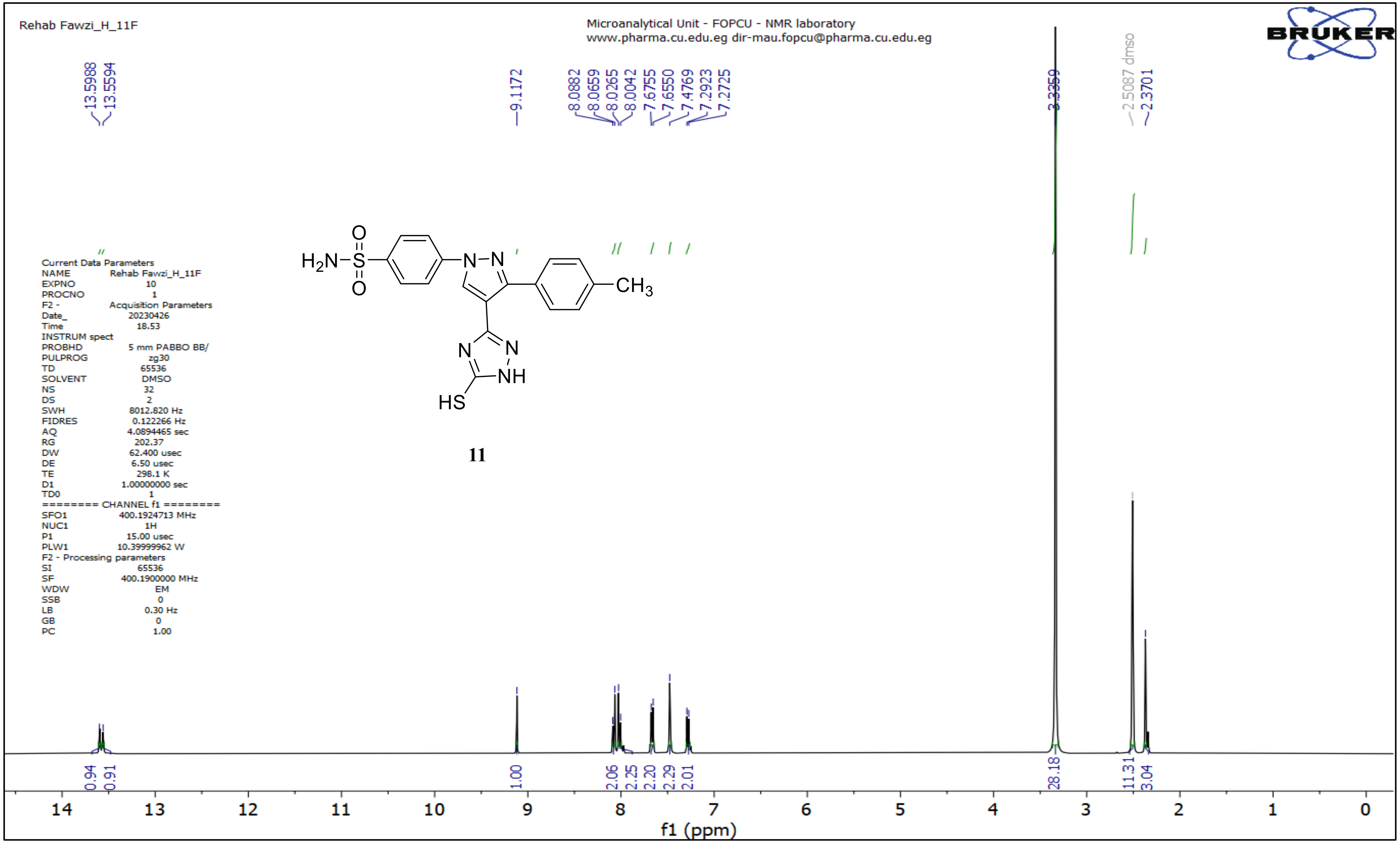

Figure S17: ^{1}H NMR spectrum of 4-[4-(5-Mercapto-1*H*-1,2,4-triazol-3-yl)-3-(*p*-tolyl)-1*H*-pyrazol-1-yl] benzenesulfonamide **(11)**.

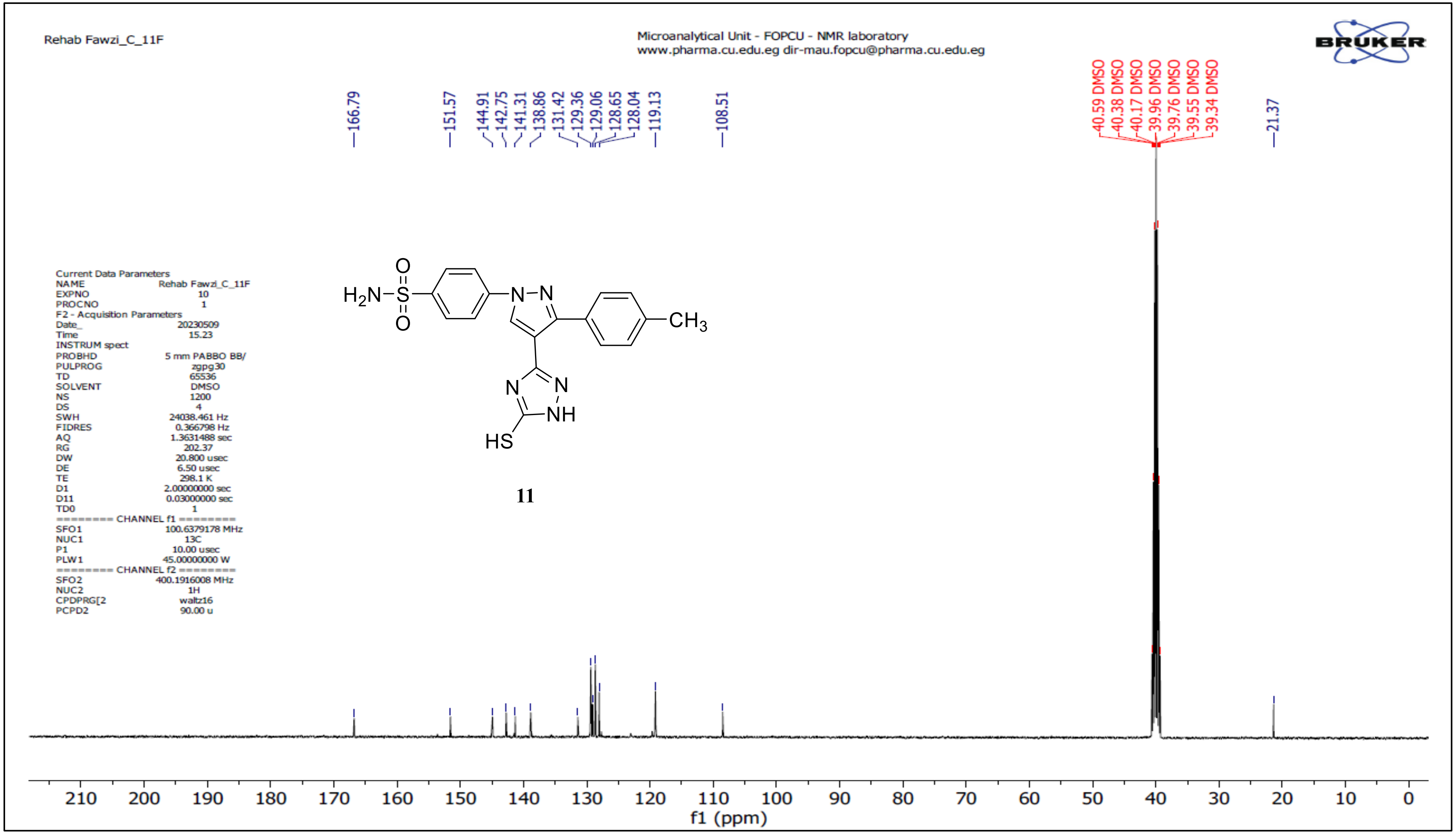

Figure S18: ^{13}C NMR spectrum of 4-[4-(5-Mercapto-1*H*-1,2,4-triazol-3-yl)-3-(*p*-tolyl)-1*H*-pyrazol-1-yl] benzenesulfonamide **(11)**.

S3- *In vitro* antiproliferative activity results against NCI-60 human tumor

Table S1: Growth inhibition percent (GI %) of compounds (**4d** and **5a-d**) against 60 different cell lines by the NCI.

Panel/ Cell line	4d	5a	5b	5c	5d
**Leukemia**					
CCRF-CEM	**60.23**	**81.96**	**75.44**	**62.61**	**85.80**
HL-60(TB)	37.58	**92.08**	**70.25**	48.35	**89.37**
K-562	**82.54**	**78.68**	**74.52**	**61.70**	**80.39**
MOLT-4	**60.09**	**88.96**	**83.81**	**65.90**	**93.98**
RPMI-8226	-	**86.85**	**64.08**	**58.60**	**80.32**
SR	22.89	**82.12**	**73.38**	**64.57**	**80.12**
**Non-Small Cell Lung Cancer**					
A549/ATCC	-	**60.32**	**51.12**	39.43	**62.90**
EKVX	20.96	48.97	**55.60**	42.24	**62.46**
HOP-62	-	19.34	-	11.98	26.17
HOP-92	49.60	38.23	**56.31**	25.99	**72.11**
NCI-H226	-	**54.74**	43.00	26.72	48.28
NCI-H23	-	**51.74**	40.30	17.53	49.44
NCI-H322M	21.48	32.36	23.66	9.97	21.22
NCI-H460	-	**63.54**	**60.81**	28.82	**65.69**
NCI-H522	19.81	**76.82**	48.97	30.99	**64.86**
**Colon Cancer**					
COLO 205	8.46	39.14	42.27	25.09	**61.21**
HCC-2998	-	**63.85**	47.04	-	**54.28**
HCT-116	**84.59**	**75.85**	**60.68**	35.19	**73.61**
HCT-15	10.55	**72.68**	**65.73**	48.54	**76.43**
HT29	-	**78.82**	**64.44**	17.27	**88.34**
KM12	-	**73.58**	**70.34**	26.16	**76.67**
SW-620	-	47.06	36.97	7.90	**50.64**

**CNS Cancer**					
SF-268	34.83	**52.23**	**55.42**	28.49	**64.08**
SF-295	-	**59.98**	**54.76**	32.73	**57.45**
SF-539	26.71	45.98	**60.94**	38.70	**63.29**
SNB-19	24.12	**60.90**	48.44	30.70	**57.92**
SNB-75	**64.33**	**60.64**	**62.89**	49.34	**74.91**
U251	24.98	**58.54**	**54.07**	34.29	**57.94**
**Melanoma**					
LOX IMVI	47.67	**69.15**	**53.58**	21.27	**72.91**
MALME-3M	-	**79.08**	**35.48**	21.01	46.12
M14	**79.60**	**63.47**	**56.85**	40.31	**73.71**
MDA-MB-435	**60.12**	45.06	47.30	27.32	**61.21**
SK-MEL-2	NT	**50.80**	27.90	23.07	49.11
SK-MEL-28	-	**54.54**	48.18	24.61	**55.62**
SK-MEL-5	14.35	**70.06**	39.32	34.89	36.27
UACC-257	-	48.03	39.15	25.39	38.33
UACC-62	11.90	**55.19**	**53.44**	44.46	**58.74**
**Panel/ Cell line**	**4d**	**5a**	**5b**	**5c**	**5d**
**Ovarian cancer**					
IGROV1	-	45.35	21.35	-	31.73
OVCAR-3	22.97	42.68	**53.48**	23.18	**62.63**
OVCAR-4	34.63	46.60	**58.80**	42.51	**63.97**
OVCAR-5	12.33	28.66	33.07	13.72	37.97
OVCAR-8	-	39.55	12.78	5.25	13.33
NCI/ADR-RES	NT	16.58	22.29	-	28.19
SK-OV-3	-	5.67	-	11.64	29.02
**Renal Cancer**					
786-0	**86.55**	**66.92**	**65.99**	**53.06**	**74.93**
A498	16.76	25.27	31.71	23.17	20.62

ACHN	-	**76.08**	**52.76**	21.30	**65.95**
CAKI-1	**63.50**	**61.60**	**70.20**	**55.62**	**81.87**
RXF 393	-	**66.45**	**73.19**	**55.31**	**82.82**
SN12C	11.85	NT	38.24	21.33	47.15
TK-10	-	40.09	13.96	23.80	18.38
UO-31	23.25	**62.74**	**58.82**	38.53	**58.85**
**Prostate Cancer**					
PC-3	23.09	**68.12**	**68.66**	**50.93**	**78.81**
DU-145	-	29.12	28.91	14.72	45.79
**Breast Cancer**					
MCF-7	**56.37**	**81.59**	**78.58**	48.76	**91.02**
MDA-MB-231/ATCC	33.88	35.79	27.81	18.79	43.66
HS 578T	36.91	44.61	**57.93**	31.27	**51.30**
BT-549	**79.94**	**64.08**	39.73	21.50	36.63
T-47D	**52.32**	**69.65**	**53.54**	46.18	**60.25**
MDA-MB-468	**66.46**	**61.97**	**52.48**	39.97	**67.57**

GI % < 5 % -
GI % > 50 %
not tested NT

Table S2: Growth inhibition percent (GI %) of compounds (**4a-c,e**, **5e**, **6a-c**, **9a-e**, **10** and **11**) against 60 different cell lines by the NCI.

**Panel/ Cell line**	**4a**	**4b**	**4c**	**4e**	**5e**	**6a**	**6b**	**6c**	**9a**	**9b**	**9c**	**9d**	**9e**	**10**	**11**
**Leukemia**															
CCRF-CEM	-	-	-	-	9.47	-	6.03	-	-	19.56	-	-	-	10.20	-
HL-60(TB)	16.64	5.09	13.91	-	6.26	-	-	-	-	-	-	-	-	9.66	-
K-562	16.66	7.16	9.61	-	9.58	-	16.74	-	-	-	-	-	-	11.07	-
MOLT-4	-	-	-	-	-	-	7.13	-	-	-	-	-	-	-	21.05
RPMI-8226	-	-	9.19	-	6.03	-	-	-	-	-	-	-	-	10.17	-
SR	-	30.24	-	-	22.09	16.99	15.69	-	-	-	-	6.97	-	-	15.75
**Non-Small Cell Lung Cancer**															
A549/ATCC	-	7.63	-	6.92	-	-	-	-	10.86	-	-	-	-	-	-
EKVX	-	-	-	-	-	9.86	7.25	11.20	-	-	-	-	-	10.54	-
HOP-62	-	-	-	-	-	-	-	-	8.30	-	-	-	-	-	-
HOP-92	-	-	-	-	-	-	22.24	27.39	-	-	-	-	-	30.76	-
NCI-H226	-	-	-	-	-	16.22	13.29	8.69	-	-	-	-	6.39	5.79	-
NCI-H23	-	-	-	-	8.79	-	9.63	-	-	-	-	-	-	10.42	-
NCI-H322M	-	6.16	-	-	-	-	-	-	-	-	-	14.95	-	-	-
NCI-H460	-	-	-	-	-	-	-	-	-	-	-	-	-	-	-
NCI-H522	-	25.63	-	23.14	13.46	13.31	-	5.91	23.86	-	8.06	8.15	12.34	15.31	-
**Colon Cancer**															
COLO 205	-	-	-	-	-	-	-	-	-	-	-	-	-	-	-
HCC-2998	-	-	-	-	-	-	-	-	-	-	-	-	-	-	-
HCT-116	27.83	-	37.53	-	-	-	-	-	-	-	-	-	-	-	-
HCT-15	-	-	-	-	-	-	-	-	-	-	-	-	-	-	-
HT29	-	11.59	-	6.67	-	-	-	-	10.78	-	-	-	-	NT	NT
KM12	-	-	-	-	-	-	-	-	-	-	-	-	-	-	-
SW-620	-	-	-	-	-	-	-	-	-	-	-	-	-	-	-
**CNS Cancer**															
SF-268	-	10.35	-	-	5.80	-	9.87	-	-	-	-	-	-	-	9.56
SF-295	-	-	-	-	-	-	-	-	-	-	-	-	-	-	9.95
SF-539	-	20.32	-	-	11.82	-	7.75	-	6.07	5.95	-	6.59	-	15.56	8.18
SNB-19	-	-	-	-	8.29	6.91	5.72	-	-	-	-	-	-	7.72	13.01
SNB-75	31.08	-	28.67	-	19.57	5.39	23.90	13.45	-	6.30	-	12.07	15.88	19.54	23.87
U251	-	-	-	-	5.78	-	-	-	-	-	-	-	-	9.42	5.33

Panel/ Cell line	4a	4b	4c	4e	5e	6a	6b	6c	9a	9b	9c	9d	9e	10	11
**Melanoma**															
LOX IMVI	-	-	-	-	11.55	8.67	12.13	8.56	-	-	-	-	-	11.75	13.48
MALME-3M	8.55	-	-	-	-	-	10.90	5.67	-	-	-	10.90	-	-	-
M14	23.17	-	43.32	-	-	-	-	-	-	-	-	-	-	-	-
MDA-MB-435	-	-	-	-	-	-	5.80	-	-	-	-	-	-	-	-
SK-MEL-2	NT	8.94	NT	-	-	-	-	-	-	-	-	-	-	-	-
SK-MEL-28	-	-	-	-	-	-	-	-	-	-	-	-	-	-	-
SK-MEL-5	-	-	-	-	-	-	23.01	-	-	-	-	-	-	18.51	-
UACC-257	-	5.42	-	6.07	-	-	-	-	-	-	-	-	7.00	5.16	-
UACC-62	-	-	-	-	15.80	17.73	14.42	9.09	8.26	5.90	8.41	-	7.82	-	12.94
**Ovarian cancer**															
IGROV1	-	-	-	-	-	-	-	-	-	-	-	20.24	-	-	-
OVCAR-3	-	-	-	-	-	-	-	-	-	-	-	-	-	-	11.44
OVCAR-4	-	-	-	-	12.31	-	16.03	-	-	-	-	-	-	-	10.09
OVCAR-5	-	5.99	-	-	7.15	-	-	-	-	-	-	-	-	-	-
OVCAR-8	-	-	-	-	-	-	-	-	-	-	-	-	-	-	-
NCI/ADR-RES	NT	-	NT	-	-	-	-	-	-	-	-	-	-	-	-
SK-OV-3	-	-	-	-	-	**54.56**	-	-	-	-	-	-	-	-	-
**Renal Cancer**															
786-0	31.20	9.87	28.93	-	13.16	NT	5.95	-	9.98	-	-	-	-	NT	NT
A498	-	-	-	-	19.73	-	7.37	7.75	-	-	-	-	-	15.52	-
ACHN	-	-	-	-	8.32	6.63	-	-	-	-	-	-	-	-	-
CAKI-1	11.58	15.19	8.36	8.93	30.76	7.52	27.98	21.51	-	7.05	-	12.06	9.93	12.09	11.44
RXF 393	-	-	-	-	-	-	-	-	-	-	-	-	-	9.90	10.09
SN12C	-	NT	-	NT	7.85	-	-	-	NT	-	NT	-	-	-	7.65
TK-10	-	-	-	-	-	-	-	-	12.34	-	-	-	-	15.39	-
UO-31	15.00	14.53	11.91	8.95	21.93	12.63	10.79	14.87	-	-	-	14.71	5.48	7.96	-
**Prostate Cancer**															
PC-3	-	-	8.81	-	-	-	7.13	-	-	-	-	-	-	8.50	-
DU-145	-	-	-	-	-	-	-	-	-	-	-	-	-	-	-
**Breast Cancer**															
MCF7	-	-	8.77	-	6.56	22.57	-	15.77	-	-	-	-	-	15.82	6.49
MDA-MB-231/ATCC	10.53	6.64	-	-	-	9.95	8.63	-	-	-	-	-	-	-	-
HS 578T	-	6.42	-	-	23.66	5.52	-	12.82	-	-	-	6.72	7.37	5.02	-

BT-549	43.40	6.70	41.65	-	18.93	-	-	-	-	-	-	-	-	11.24	-
T-47D	-	17.49	-	-	19.13	-	-	-	-	-	-	7.04	-	13.20	21.21
MDA-MB-468	-	18.57	-	-	20.27	-	-	7.76	-	-	-	-	7.21	5.43	-

GI % < 5 % -
GI % > 50 %
not tested NT

S4. The data used to calculate the IC_{50} in hCA IX

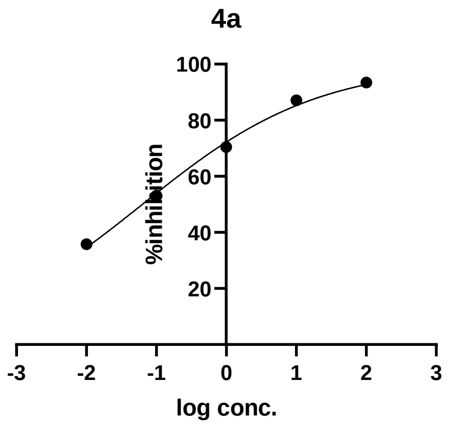

Figure S19: Graph representing log (concentration) against % Inhibition for compound **4a**.

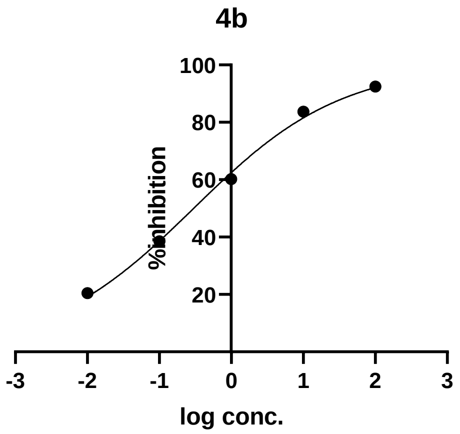

Figure S20: Graph representing log (concentration) against % Inhibition for compound **4b**.

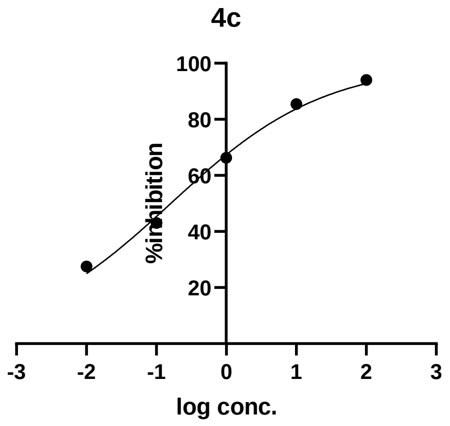

Figure S21: Graph representing log (concentration) against % Inhibition for compound **4c**.

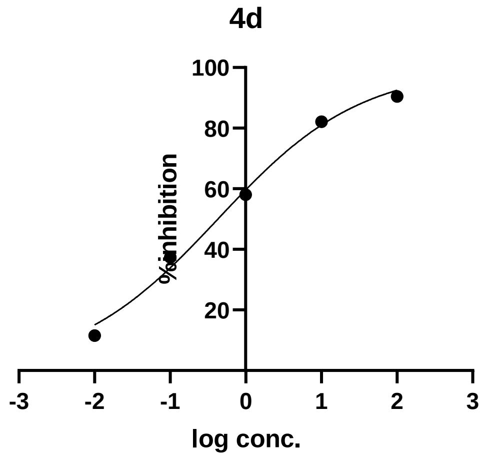

Figure S22: Graph representing log (concentration) against % Inhibition for compound **4d**.

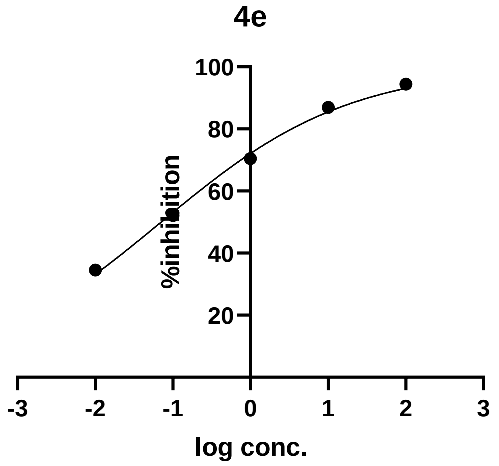

Figure S23: Graph representing log (concentration) against % Inhibition for compound **4e**.

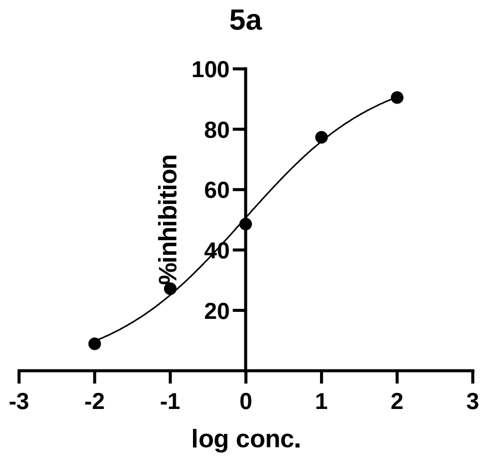

Figure S24: Graph representing log (concentration) against % Inhibition for compound **5a**.

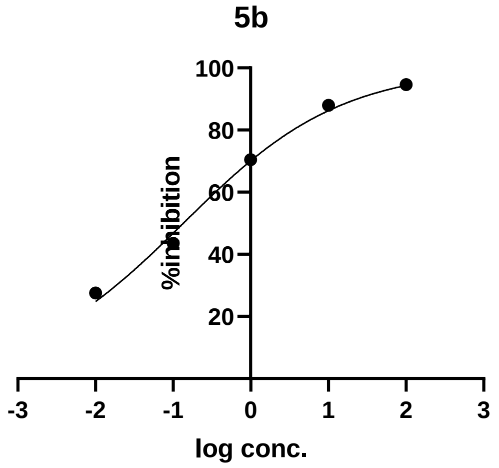

Figure S25: Graph representing log (concentration) against % Inhibition for compound **5b**.

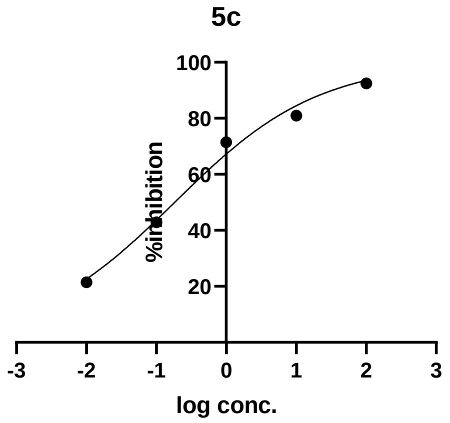

Figure S26: Graph representing log (concentration) against % Inhibition for compound **5c**.

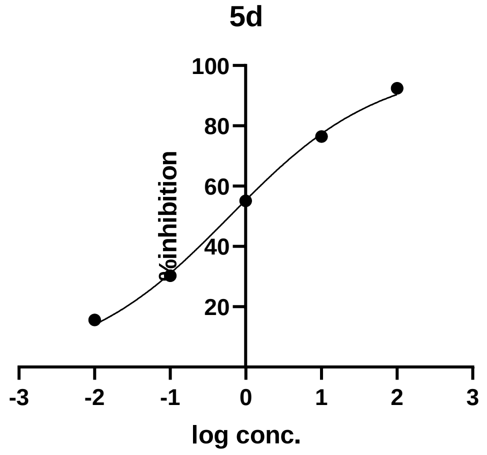

Figure S27: Graph representing log (concentration) against % Inhibition for compound **5d**.

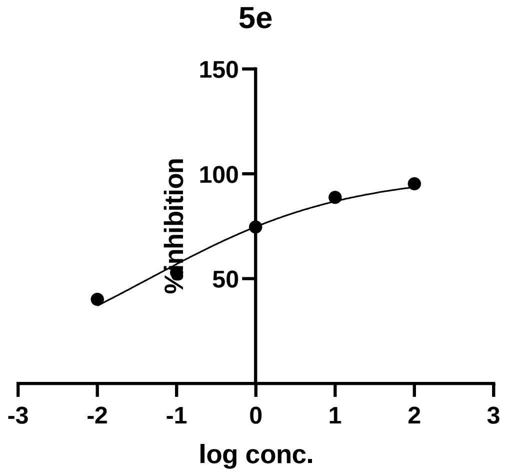

Figure S28: Graph representing log (concentration) against % Inhibition for compound **5e**.

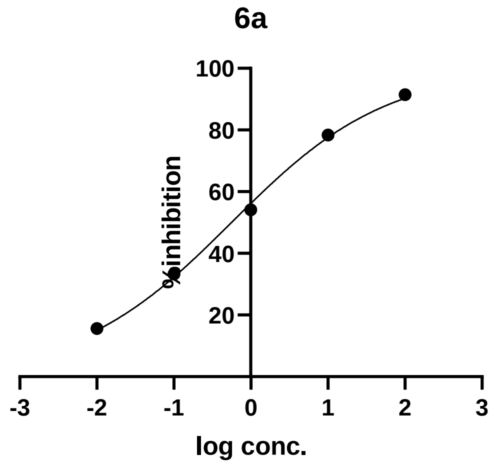

Figure S29: Graph representing log (concentration) against % Inhibition for compound **6a**.

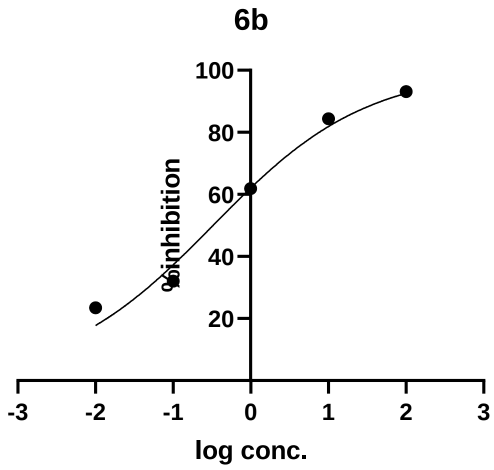

Figure S30: Graph representing log (concentration) against % Inhibition for compound **6b**.

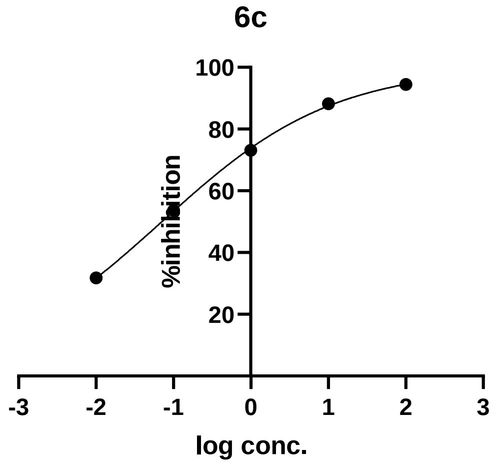

Figure S31: Graph representing log (concentration) against % Inhibition for compound **6c**.

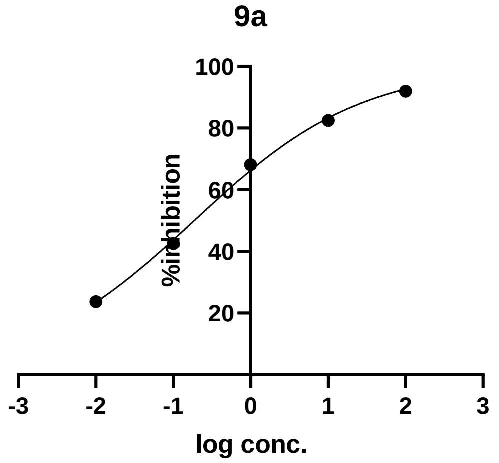

Figure S32: Graph representing log (concentration) against % Inhibition for compound **9a**.

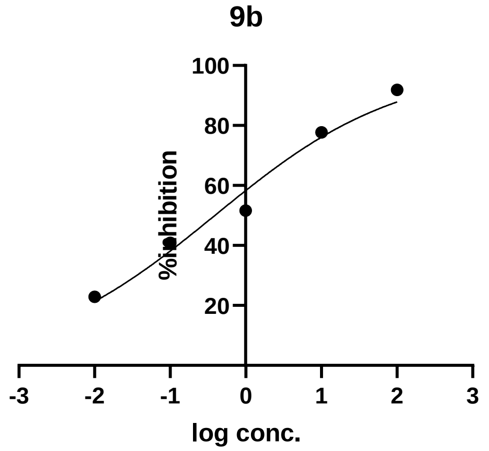

Figure S33: Graph representing log (concentration) against % Inhibition for compound **9b**.

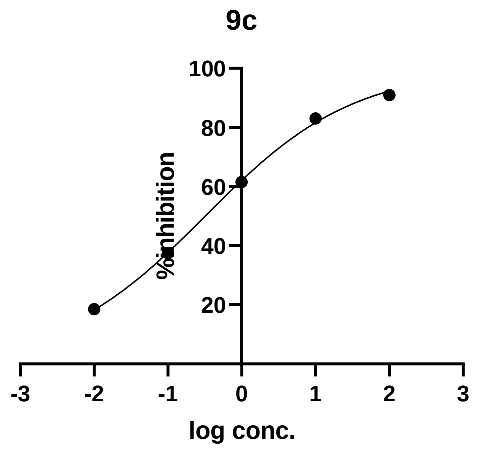

Figure S34: Graph representing log (concentration) against % Inhibition for compound **9c**.

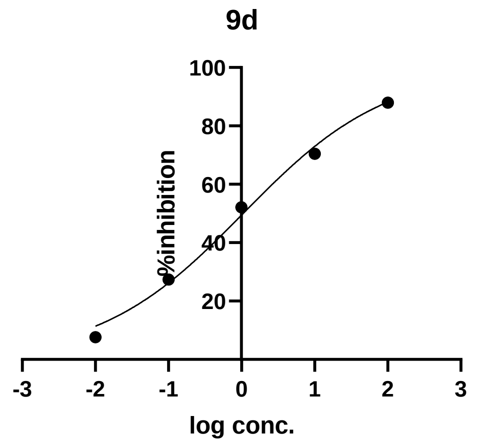

Figure S35: Graph representing log (concentration) against % Inhibition for compound **9d**.

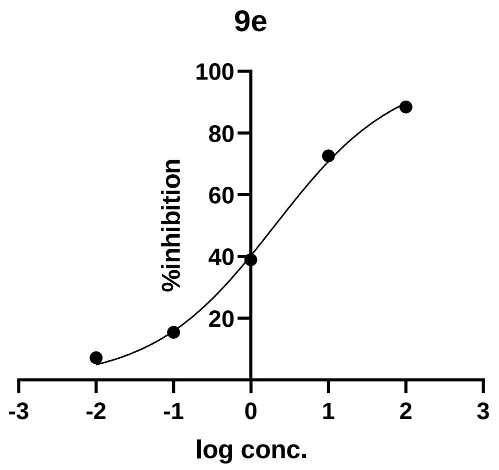

Figure S36: Graph representing log (concentration) against % Inhibition for compound **9e**.

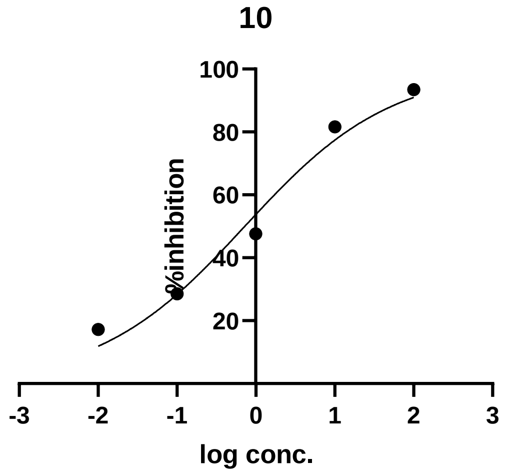

Figure S37: Graph representing log (concentration) against % Inhibition for compound **10**.

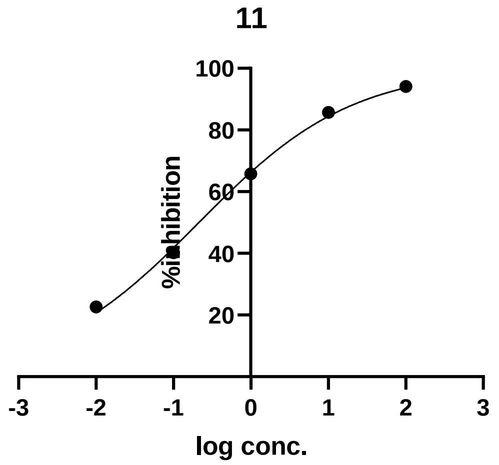

Figure S38: Graph representing log (concentration) against % Inhibition for compound **11**.

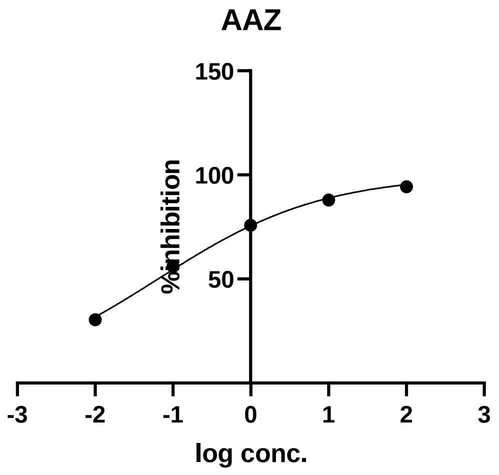

Figure S39: Graph representing log (concentration) against % Inhibition for compound (acetazolamide) **AAZ**.

S5. The data used to calculate the IC_{50} in hCA XII

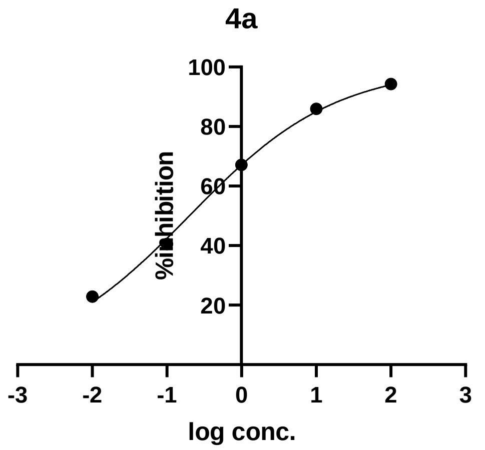

Figure S40: Graph representing log (concentration) against % Inhibition for compound **4a**.

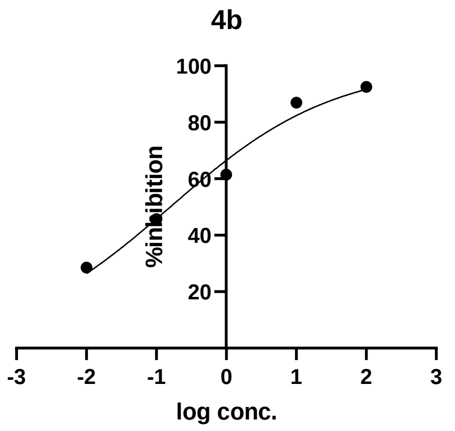

Figure S41: Graph representing log (concentration) against % Inhibition for compound **4b**.

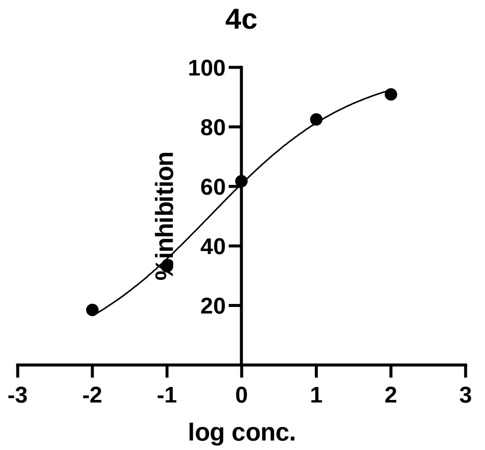

Figure S42: Graph representing log (concentration) against % Inhibition for compound **4c**.

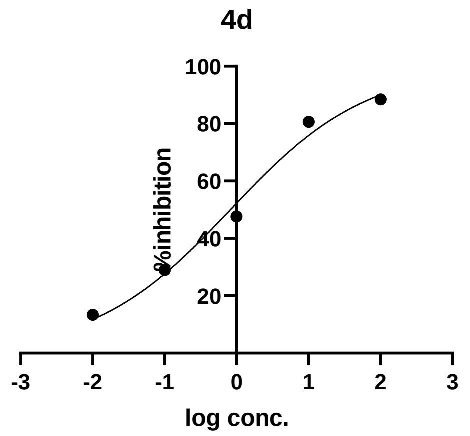

Figure S43: Graph representing log (concentration) against % Inhibition for compound **4d**.

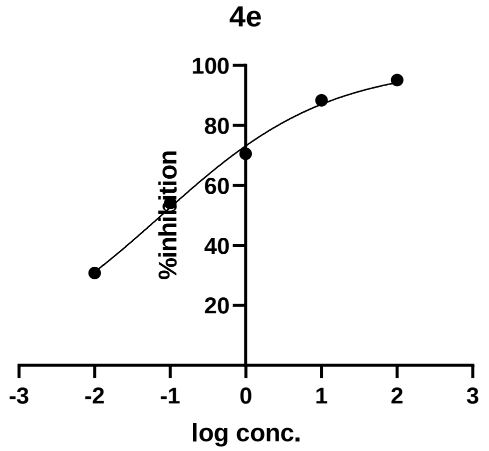

Figure S44: Graph representing log (concentration) against % Inhibition for compound **4e**.

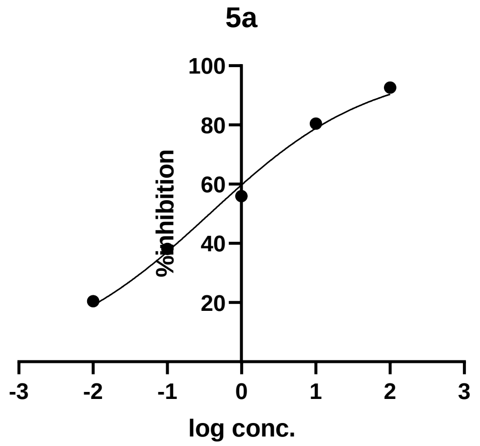

Figure S45: Graph representing log (concentration) against % Inhibition for compound **5a**.

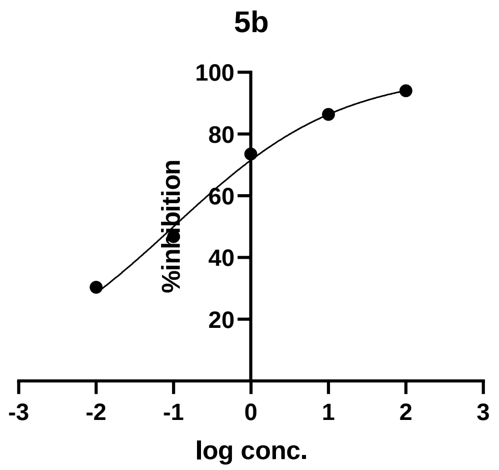

Figure S46: Graph representing log (concentration) against % Inhibition for compound **5b**.

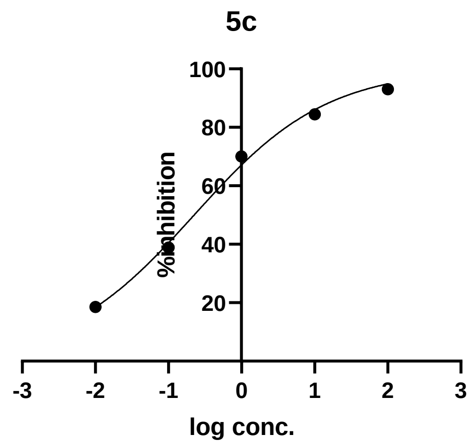

Figure S47: Graph representing log (concentration) against % Inhibition for compound **5c**.

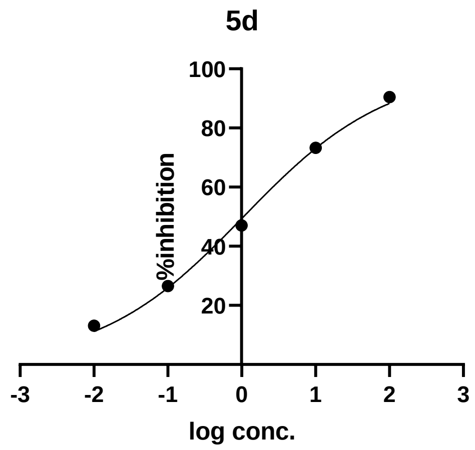

Figure S48: Graph representing log (concentration) against % Inhibition for compound **5d**.

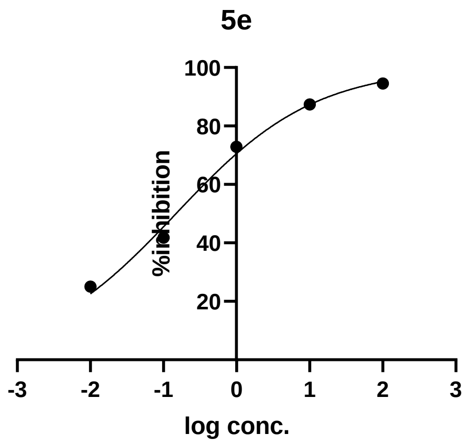

Figure S49: Graph representing log (concentration) against % Inhibition for compound **5e**.

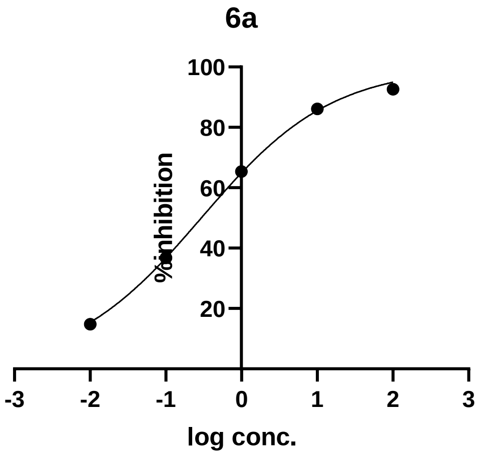

Figure S50: Graph representing log (concentration) against % Inhibition for compound **6a**.

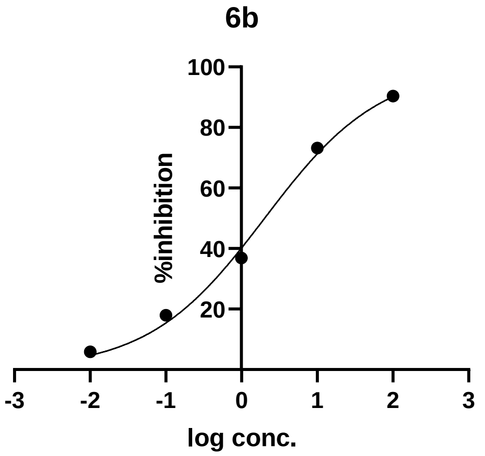

Figure S51: Graph representing log (concentration) against % Inhibition for compound **6b**.

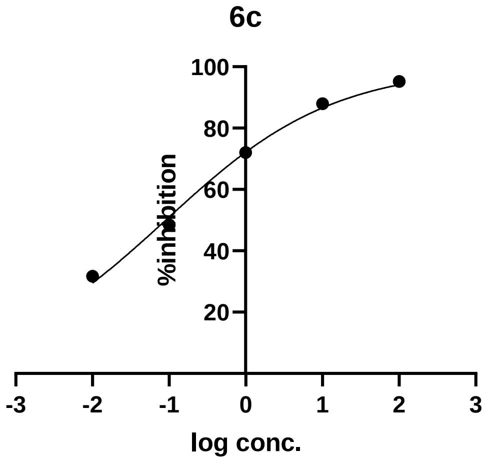

Figure S52: Graph representing log (concentration) against % Inhibition for compound **6c**.

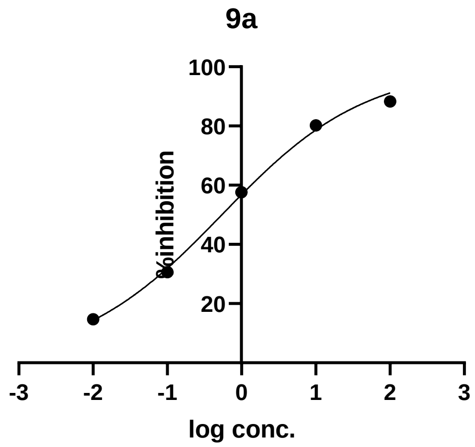

Figure S53: Graph representing log (concentration) against % Inhibition for compound **9a**.

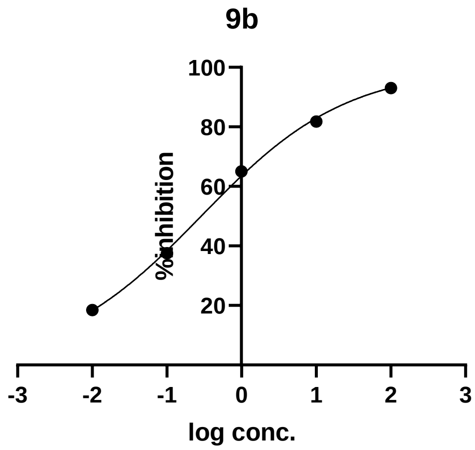

Figure S54: Graph representing log (concentration) against % Inhibition for compound **9b**.

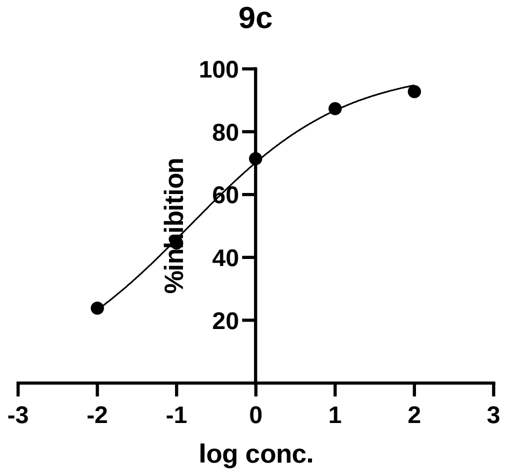

Figure S55: Graph representing log (concentration) against % Inhibition for compound **9c**.

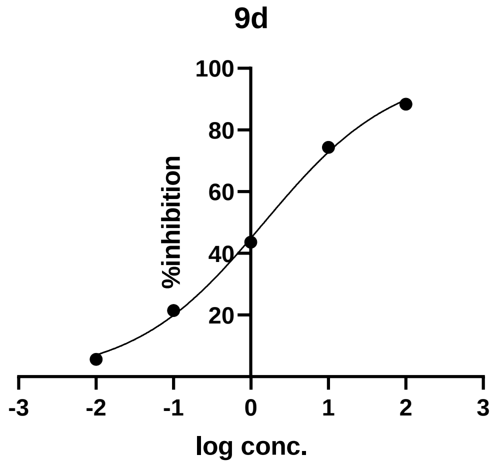

Figure S56: Graph representing log (concentration) against % Inhibition for compound **9d**.

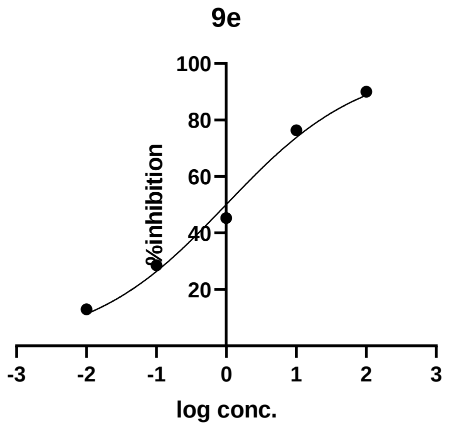

Figure S57: Graph representing log (concentration) against % Inhibition for compound **9e**.

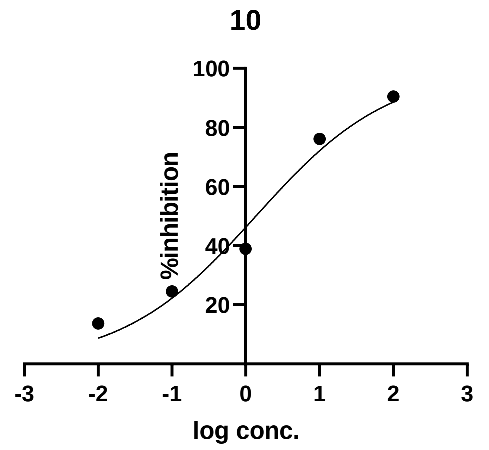

Figure S58: Graph representing log (concentration) against % Inhibition for compound **10**.

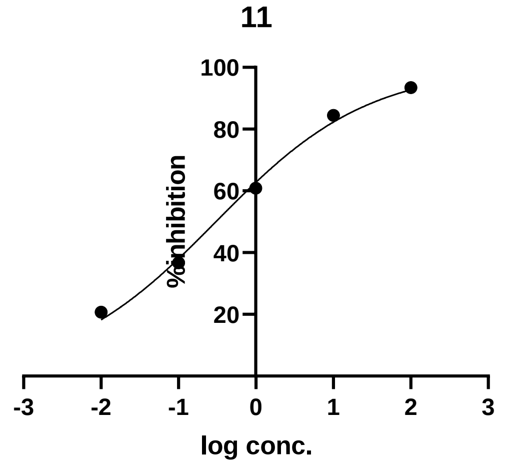

Figure S59: Graph representing log (concentration) against % Inhibition for compound **11**.

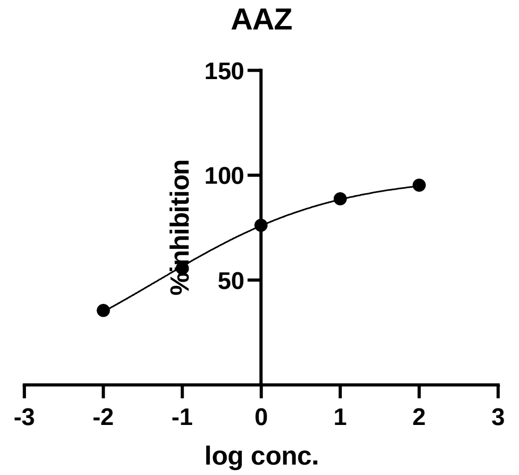

Figure S60: Graph representing log (concentration) against % Inhibition for compound (acetazolamide) **AAZ**.

S6. Molecular docking study:

S.6.1. Experimental methodology:

The proteins were downloaded from the protein data bank, https://www.rcsb.org/. First, chain A was only kept in case of each protein and water molecules and ligands not involved in binding were removed. Then, the protein structures were prepared for docking study using QuickPrep protocol in MOE. The co-crystallized ligand in each isozyme was used to define the binding site for docking. Triangle Matcher placement method and London dG scoring function were used for docking. Then, docking setup was validated by self-docking of the co-crystallized ligand in each protein in the active site of the enzyme. The self-docking validation step showed the suitability of the used docking protocol for the intended docking study by the small RMSD values and by the ability of the docking poses of the co-crystallized to reproduce all the key interactions accomplished by the co-crystallized ligand with the hot spots in CA IX and CA XII active sites (Zn^{+2}, Thr199 and/or Thr200). Then, the validated setup was used to study the binding mode of the newly synthesized compounds in the different carbonic anhydrase isoforms CA IX and CA XII and to predict their potential affinity to these isoforms.

S.6.2. Tables of most important docking parameters:

Table S3: The docking score, binding interactions, hydrogen bond length and IC_{50} of the most active compounds in hCA IX enzyme.

Compound	Docking score S (Kcal/mol)	Amino acid interactions	Interacting groups	H-bond Length (A°)	IC_{50} (µM) ± SD
**9FK**	-9.9253	Zn^{+2} (metal)<br>Leu199 (π interaction)<br>Thr200	NH and O of SO_2NH_2<br>Thiophene ring<br>O of SO_2NH_2	 <br>2.93	-
**4a**	-10.3314	Zn^{+2} (metal)<br>His68<br>Gln71 (π interaction)<br>Gln71<br>Thr200	NH and O of SO_2NH_2<br>N of pyrazole<br>Pyrazole ring<br>NHCO<br>O of SO_2NH_2	 3.86<br><br>3.25<br>2.94	0.062±0.003
**4e**	-12.3063	Zn^{+2} (metal)<br>Leu91 (π interaction)<br>Leu199 (π interaction)<br>Thr200	NH and O of SO_2NH_2<br>Benzene ring<br>Benzenesulfonamide phenyl ring<br>O of SO_2NH_2	 <br><br>2.96	0.072±0.003
**5e**	-10.4106	Zn^{+2} (metal)<br>Leu199 (π interaction)<br>Thr200	NH and O of SO_2NH_2<br>Benzenesulfonamide phenyl ring<br>O of SO_2NH_2	 <br>2.94	0.04±0.002
**6c**	-11.1956	Zn^{+2} (metal)	NH and O of SO_2NH_2		0.073±0.003

		Arg129<br>Leu199 (π interaction)<br>Thr200	O of SO_2NH_2 of acetazolamide<br>Benzenesulfonamide phenyl ring<br>O of SO_2NH_2	3.21<br><br>2.90	

Table S4: The docking score, binding interactions, hydrogen bond length and IC_{50} of the most active compounds in hCA XII enzyme.

Compound	Docking scores (Kcal/mol)	Amino acid interactions	Interacting groups	H-bond Length (A°)	IC_{50} (μM) ± SD
**AAZ**	-9.1868	Zn^{+2} (metal)<br>Leu198 (π interaction)<br>Thr199<br>Thr200	NH and O of SO_2NH_2<br>Thiadiazole ring<br>N of thiadiazole ring<br>N of thiadiazole ring	 <br>2.85<br>3.05	0.046±0.002
**4e**	-12.4889	Zn^{+2} (metal)<br>Lys67<br>Asn69<br>Leu198<br>Thr199	NH of SO_2NH_2<br>2 carbonyl groups<br>O of added SO_2NH_2<br>O of SO_2NH_2<br>O of SO_2NH_2	 3.17, 3.20<br>3.12<br>3.24<br>3.12	0.081±0.003
**5b**	-11.9156	Zn^{+2} (metal)<br>Lys67<br>Gln92<br>Leu198<br>Thr199	NH and O of SO_2NH_2<br>Carbonyl group<br>NH<br>O of SO_2NH_2<br>O of SO_2NH_2	 3.29<br>3.31<br>3.30<br>3.31	0.106±0.004
**6c**	-13.0829	Zn^{+2} (metal)<br>His64<br>Lys67<br>Lys170<br>Leu198<br>Thr199	NH and O of SO_2NH_2<br>NH of SO_2NH_2 of acetazolamide<br>Carbonyl group<br>O of SO_2NH_2 of acetazolamide<br>O of SO_2NH_2<br>O of SO_2NH_2	 3.10<br>3.21<br>3.01<br>3.24<br>2.99	0.095±0.004

S.6.3 The 2D representations of the docking poses of the co-crystallized ligands and remaining promising compounds.

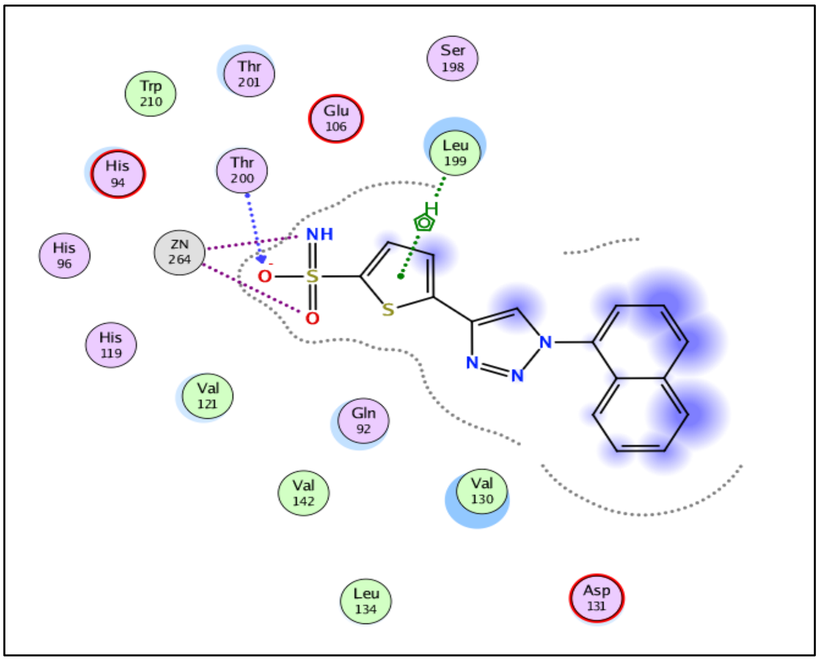

Figure S61: 2D Representation of the interaction of **9FK** in the active pocket of hCA IX enzyme

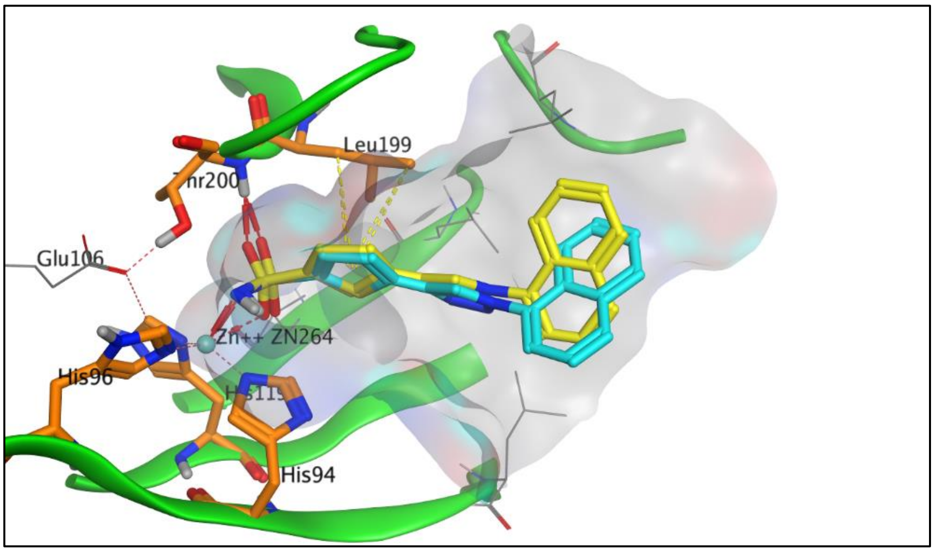

Figure S62: 3D Representation of the co-crystallized (yellow colour) and re-docked (cyan colour) ligand **9FK** in the active site of hCA IX enzyme.

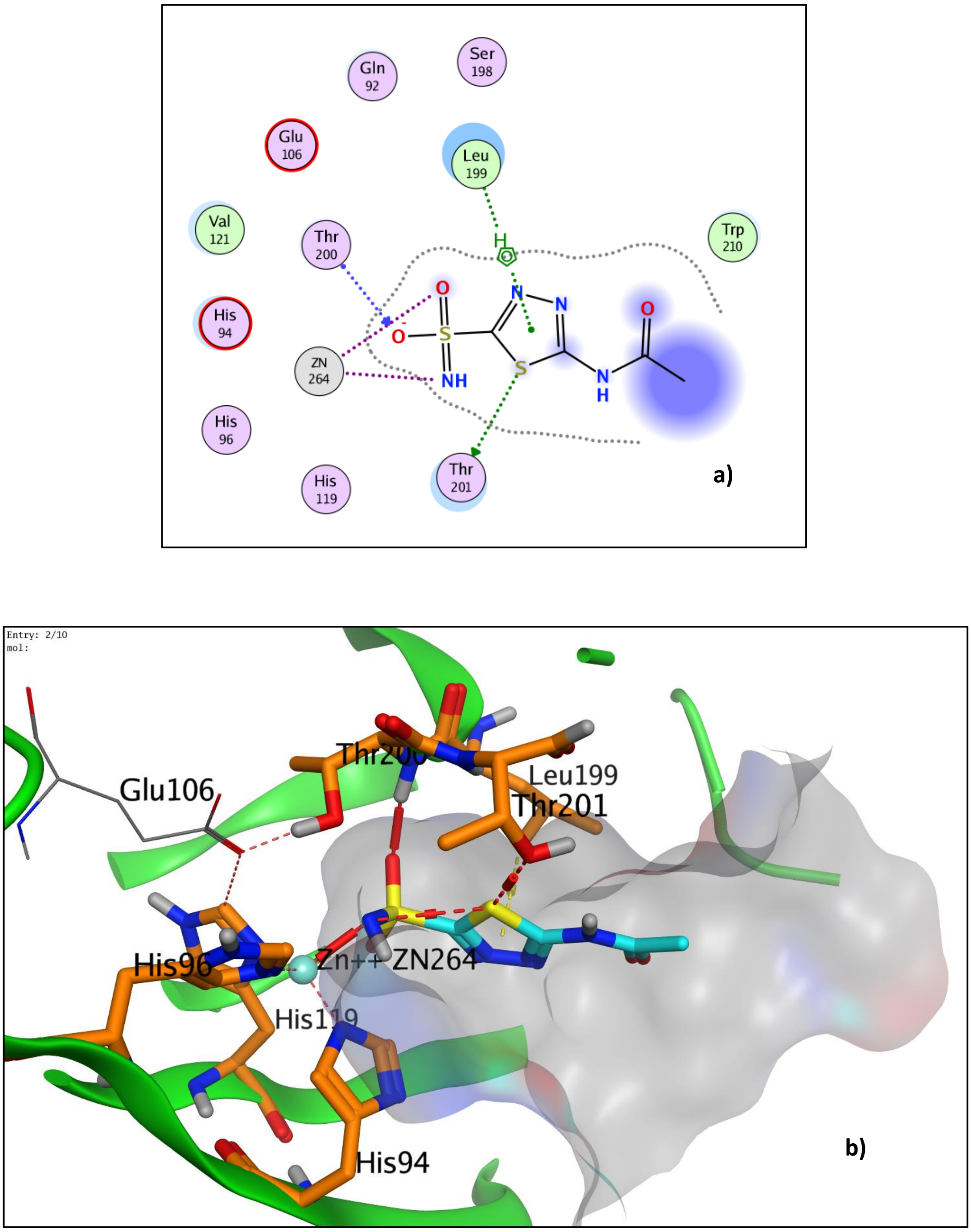

Figure S63: 2D diagram **(a)** and 3D representation **(b)** of the experimentally used reference **AAZ** in the hCA IX binding site.

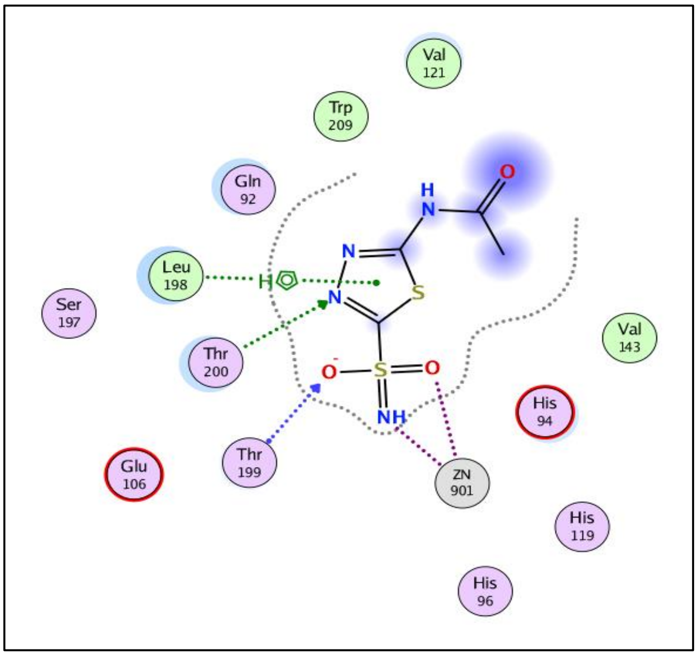

Figure S64: 2D Representation of ligand **AAZ** in the active pocket of hCA XII enzyme.

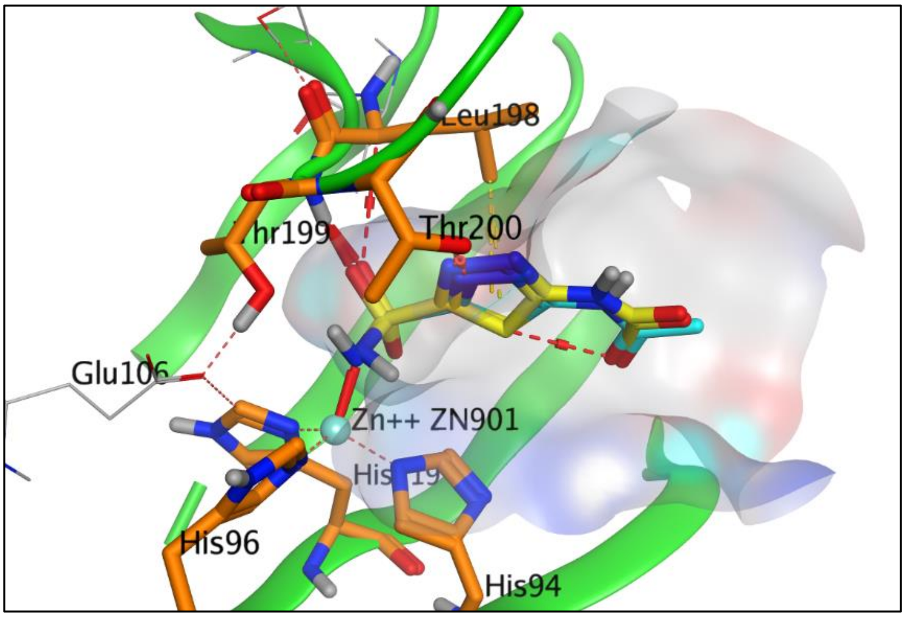

Figure S65: 3D Representation of the co-crystallized (yellow colour) and re-docked (cyan colour) ligand **AAZ** in the active site of hCA XII enzyme.

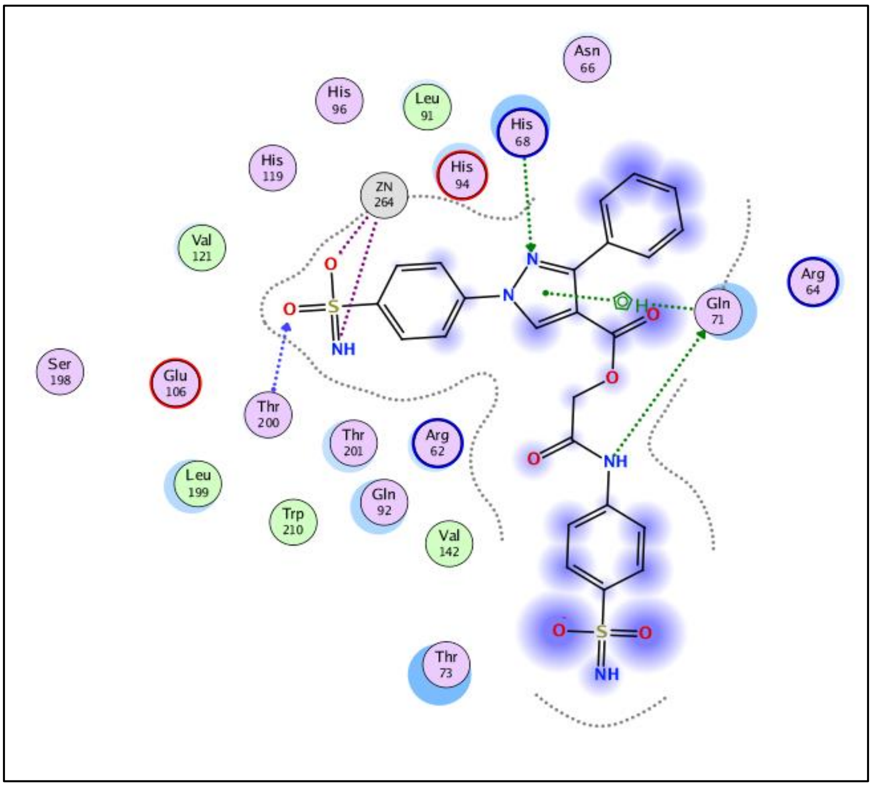

Figure S66: 2D representation of compound **4a** in the hCA IX binding site.

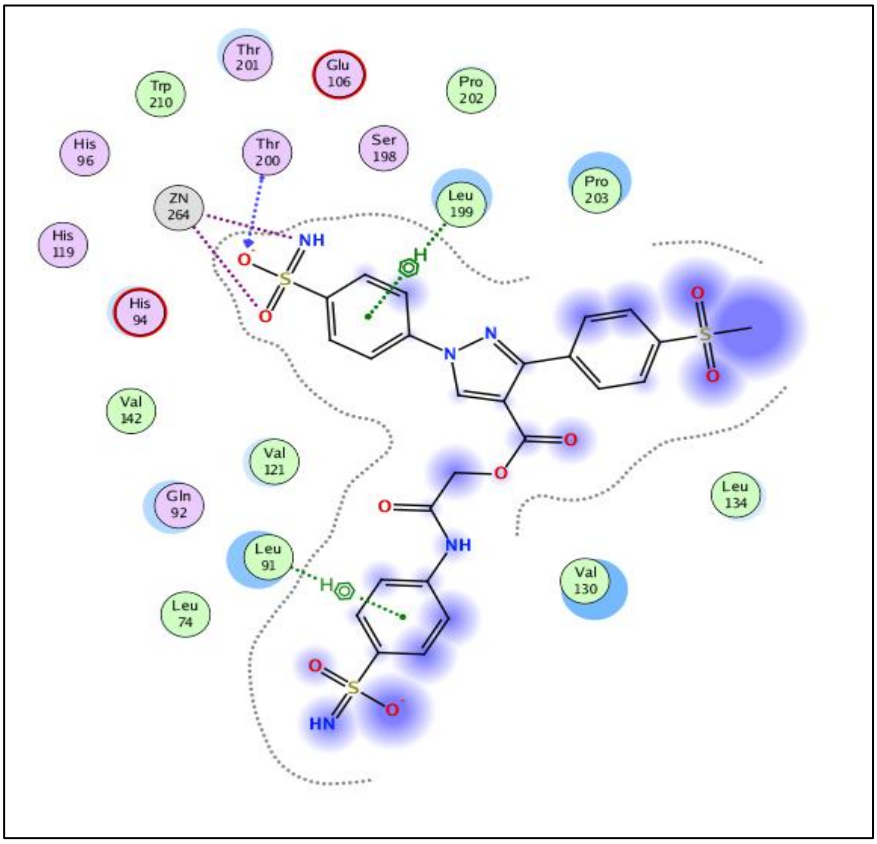

Figure S67: 2D representation of compound **4e** in the hCA IX binding site.

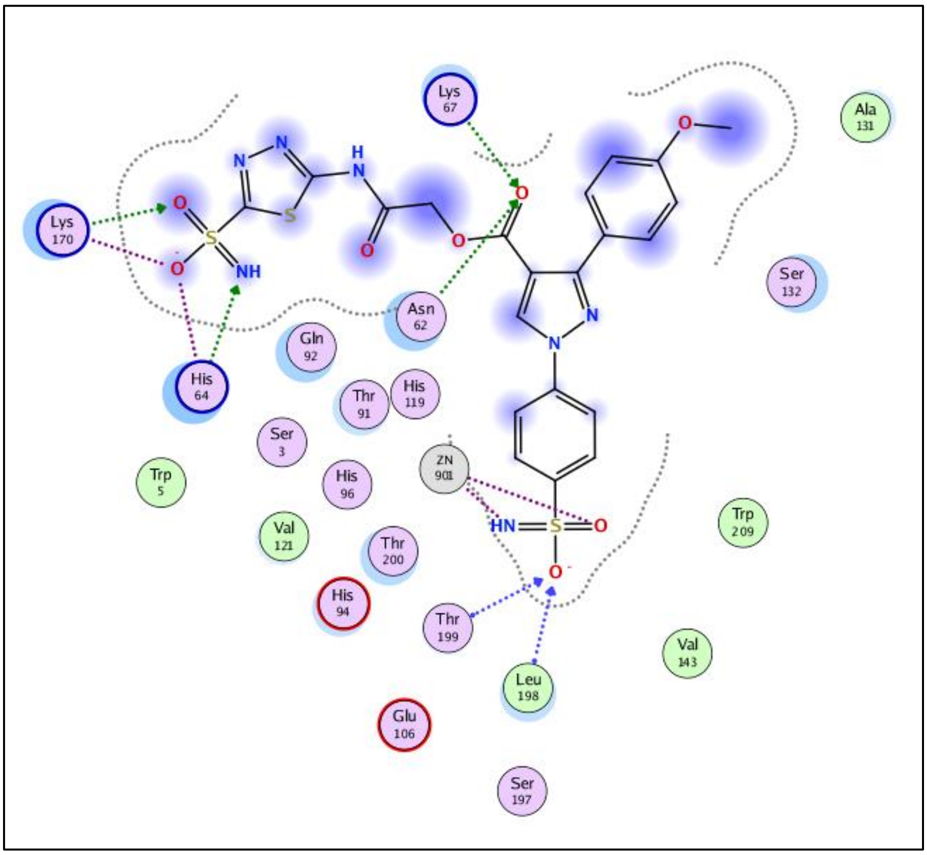

Figure S68: 2D representation of compound **6c** in the hCA XII binding site.

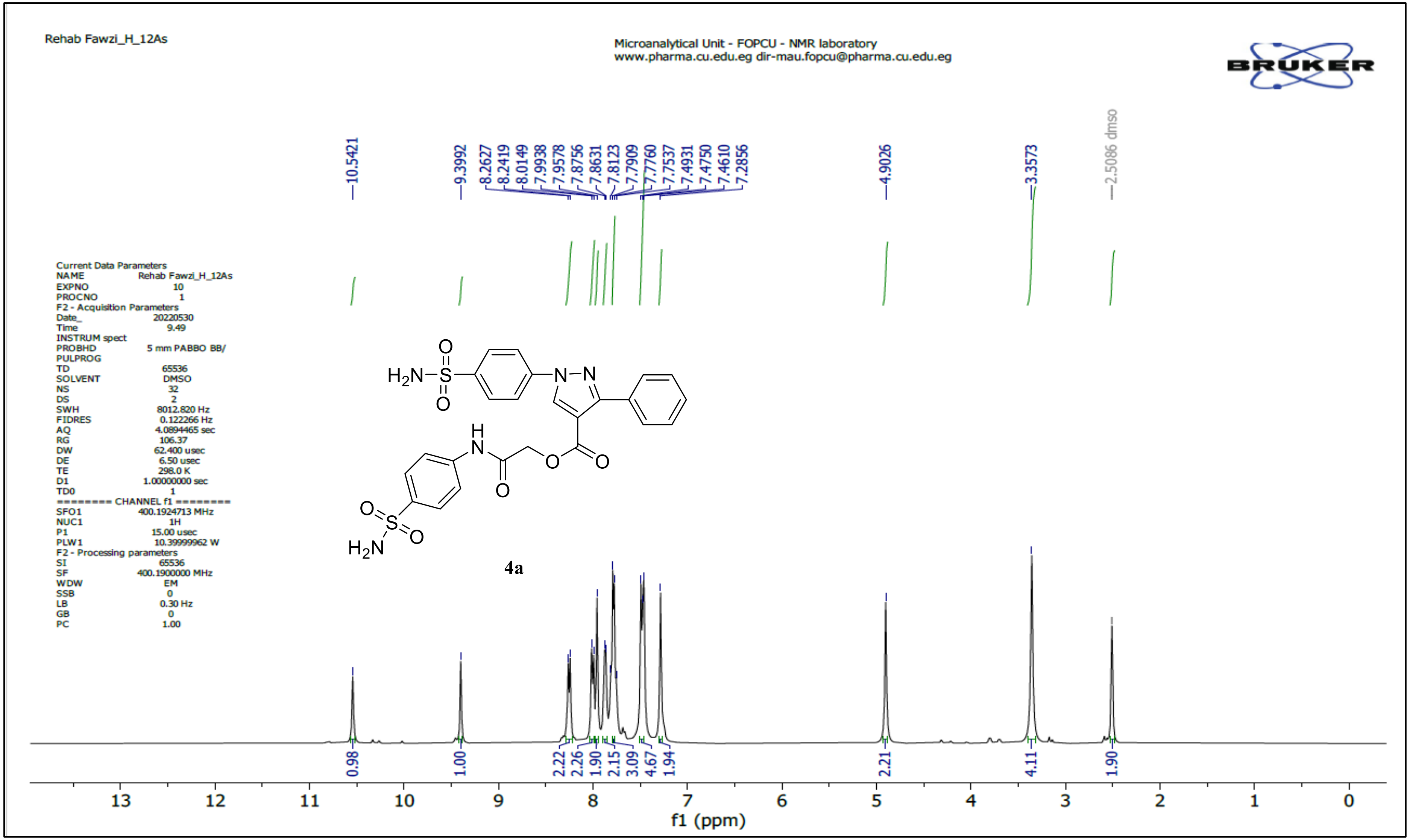

Figure S69: ^{1}H NMR spectrum of 2-Oxo-2-[(4-sulfamoylphenyl) amino] ethyl 3-phenyl-1-(4-sulfamoylphenyl)-*1H*-pyrazole-4-carboxylate **(4a)**.

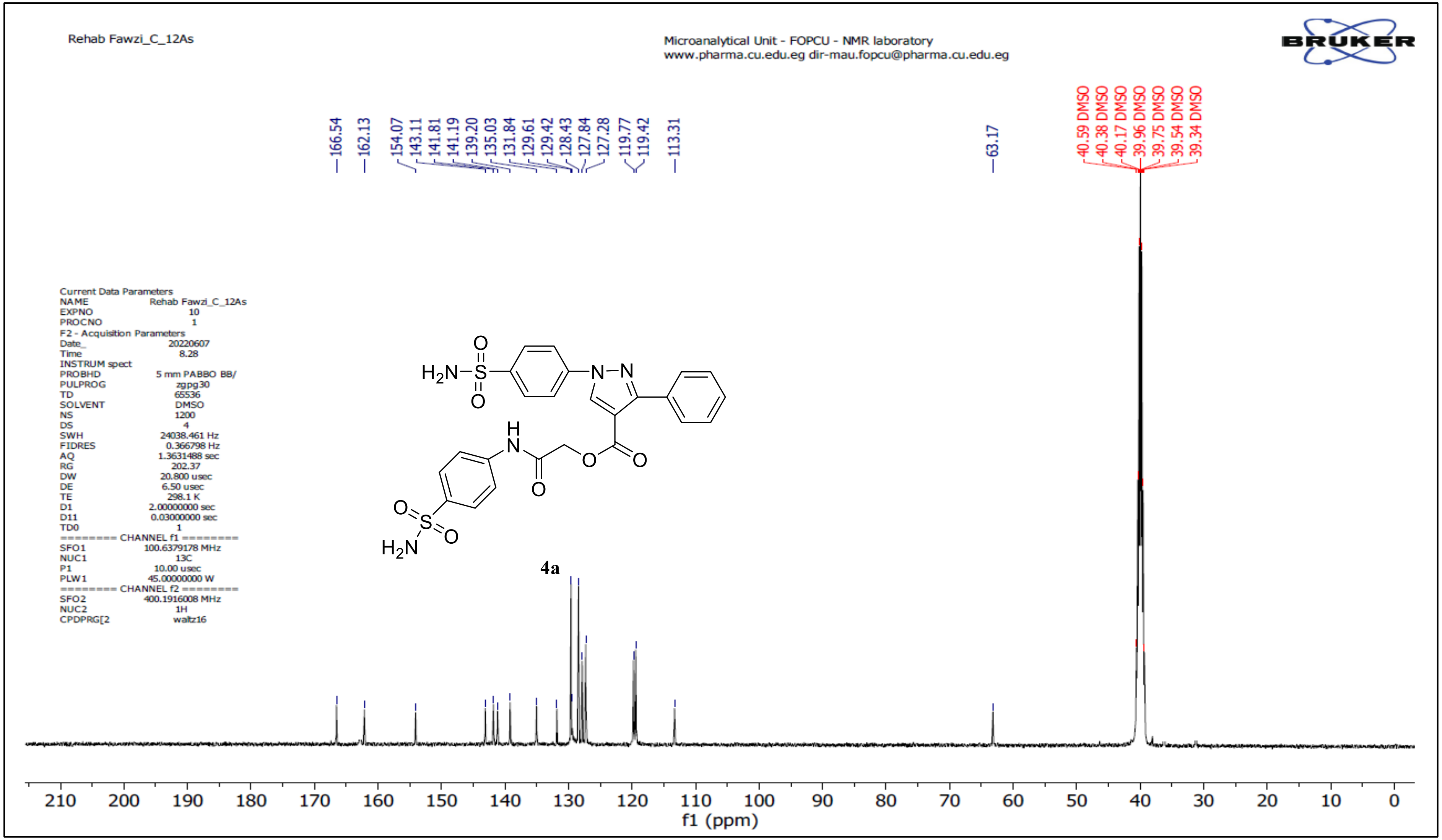

Figure S70: ^{13}C NMR spectrum of 2-Oxo-2-[(4-sulfamoylphenyl) amino] ethyl 3-phenyl-1-(4-sulfamoylphenyl)-*1H*-pyrazole-4-carboxylate **(4a)**.

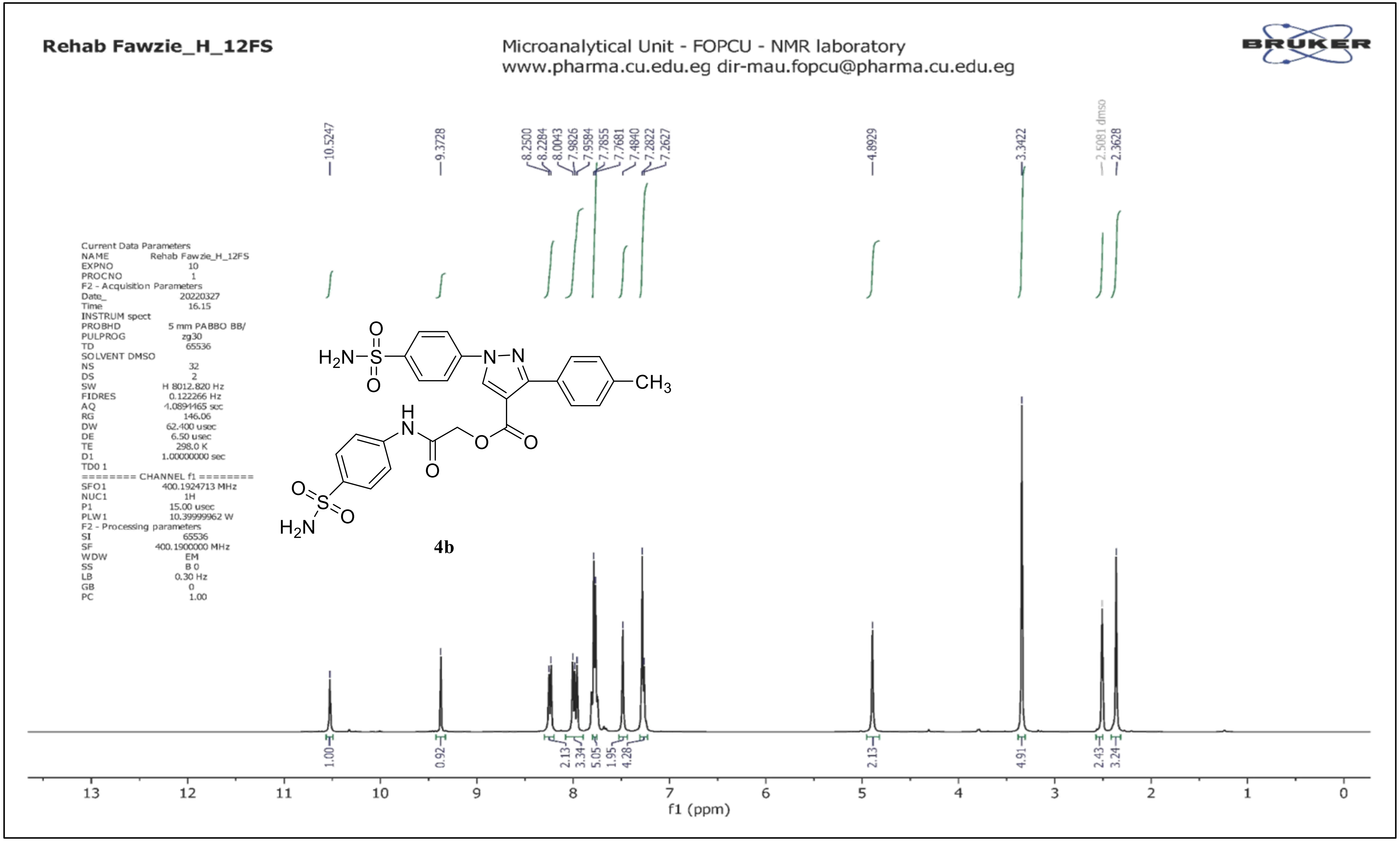

Figure S71: ^{1}H NMR spectrum of 2-Oxo-2-[(4-sulfamoylphenyl) amino] ethyl 1-(4-sulfamoylphenyl)-3-(*p*-tolyl)-1*H*-pyrazole-4-carboxylate **(4b)**

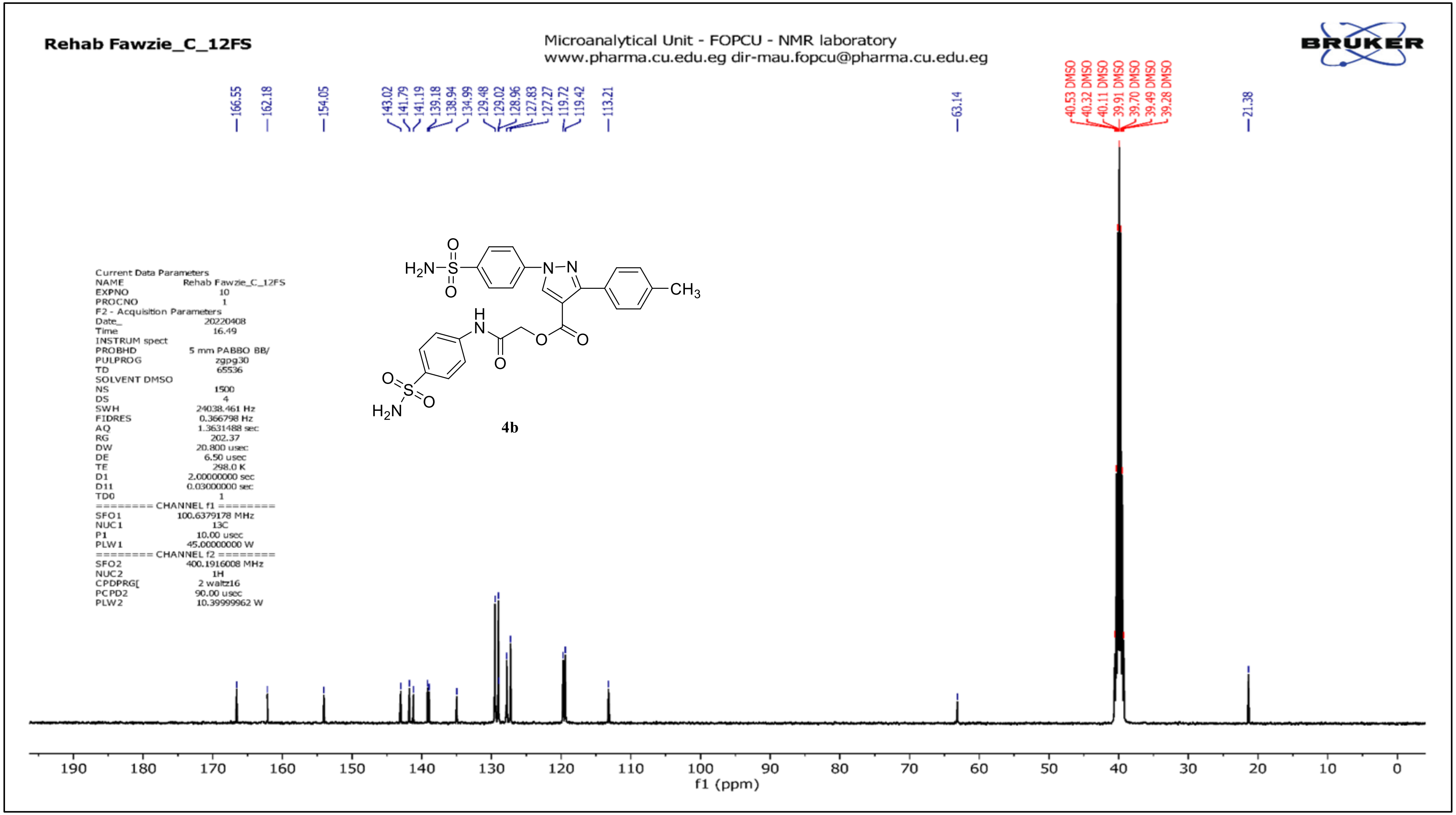

Figure S72: ^{13}C NMR spectrum of 2-Oxo-2-[(4-sulfamoylphenyl) amino] ethyl 1-(4-sulfamoylphenyl)-3-(*p*-tolyl)-1*H*-pyrazole-4-carboxylate **(4b)**

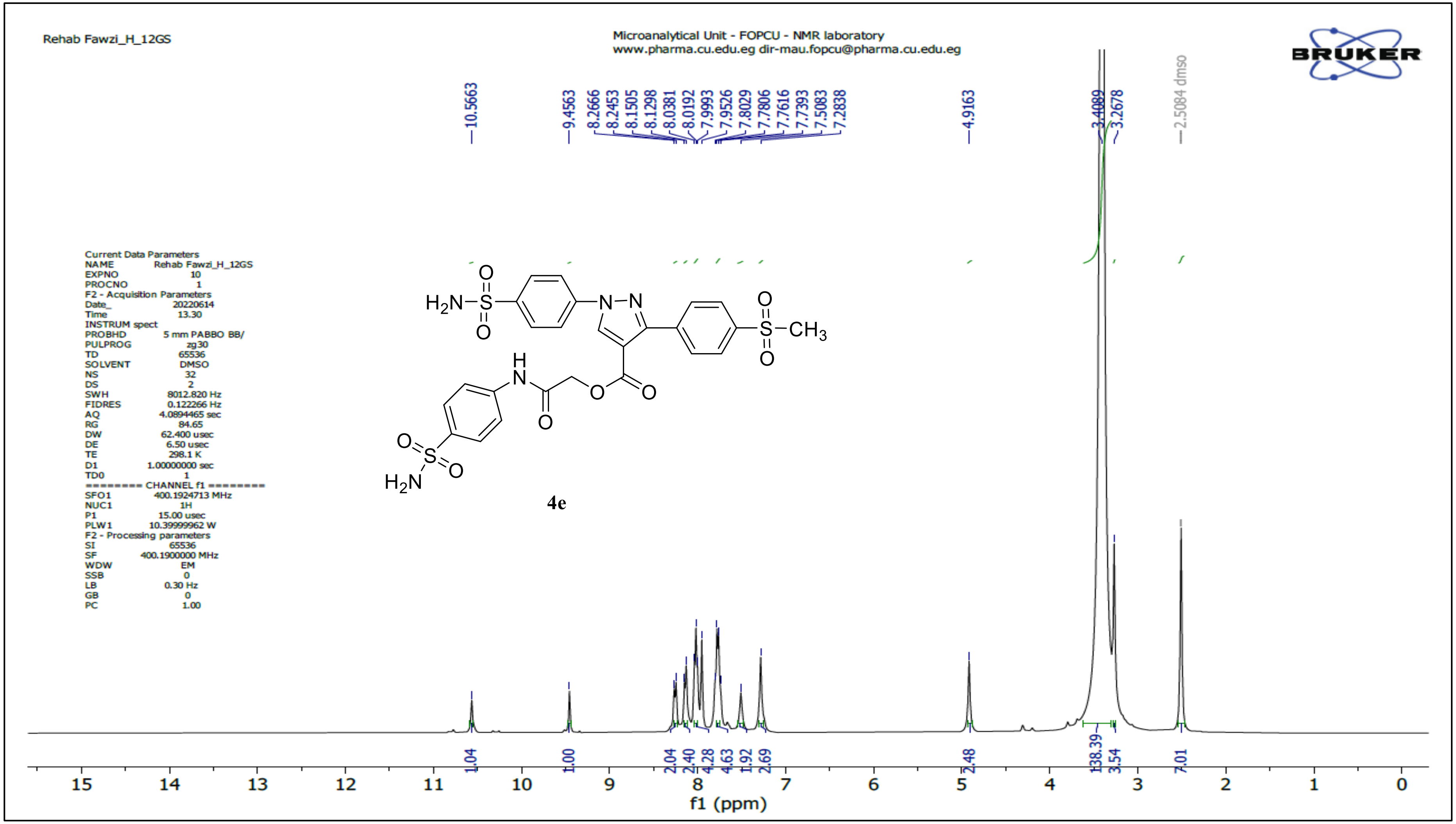

Figure S73: ^{1}H NMR spectrum of 2-Oxo-2-[(4-sulfamoylphenyl) amino] ethyl 3-(4-(methylsulfonyl) phenyl)-1-(4-sulfamoylphenyl)-*1H*-pyrazole-4-carboxylate **(4e)**

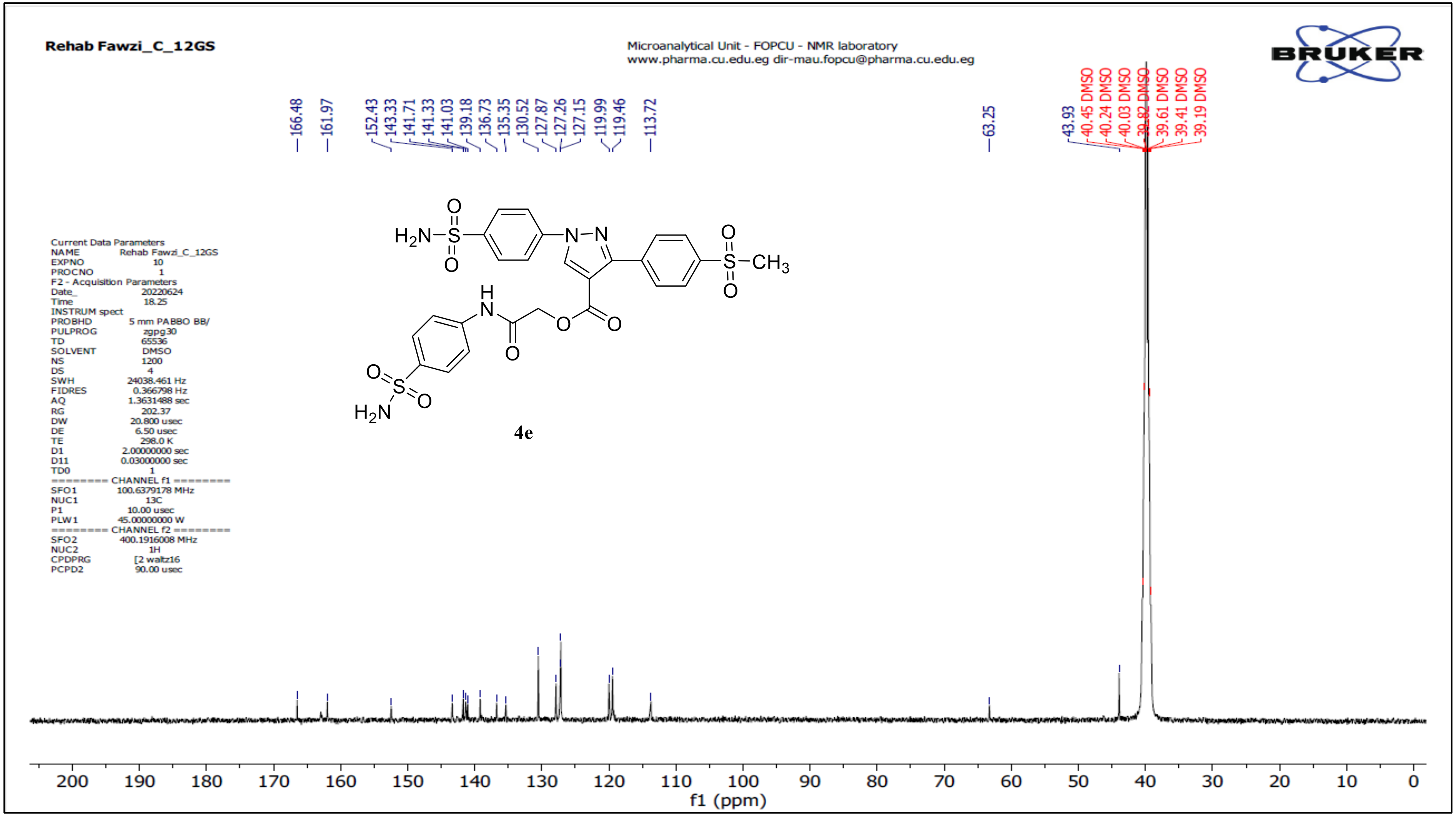

Figure S74: ^{13}C NMR spectrum of 2-Oxo-2-[(4-sulfamoylphenyl) amino] ethyl 3-(4-(methylsulfonyl) phenyl)-1-(4-sulfamoylphenyl)-*1H*-pyrazole-4-carboxylate **(4e)**

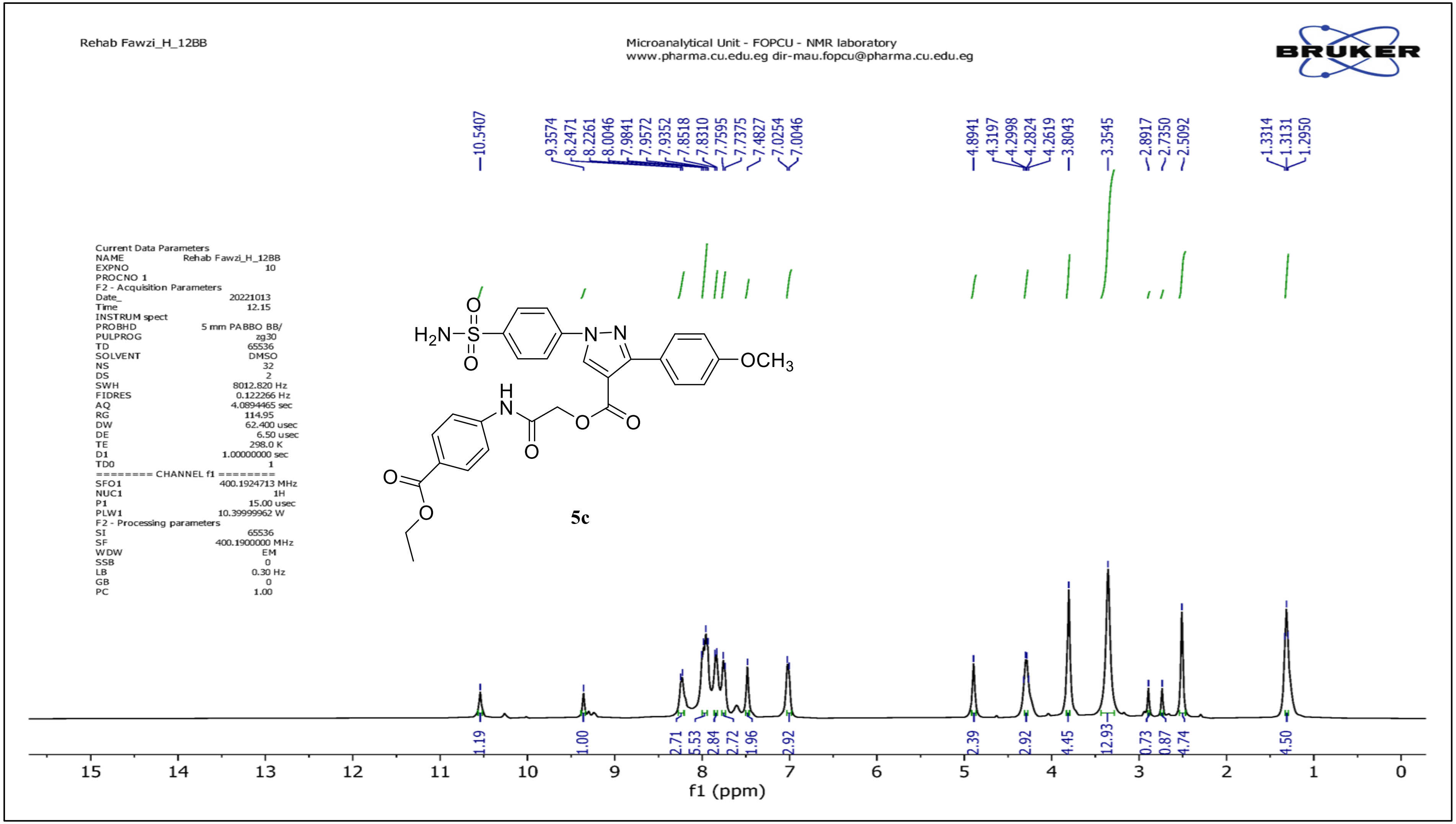

Figure S75: ^{1}H NMR spectrum of 2-[(4-(Ethoxycarbonyl) phenyl) amino]-2-oxoethyl 3-(4-methoxyphenyl)-1-(4-sulfamoylphenyl)-1*H*-pyrazole-4-carboxylate **(5c)**

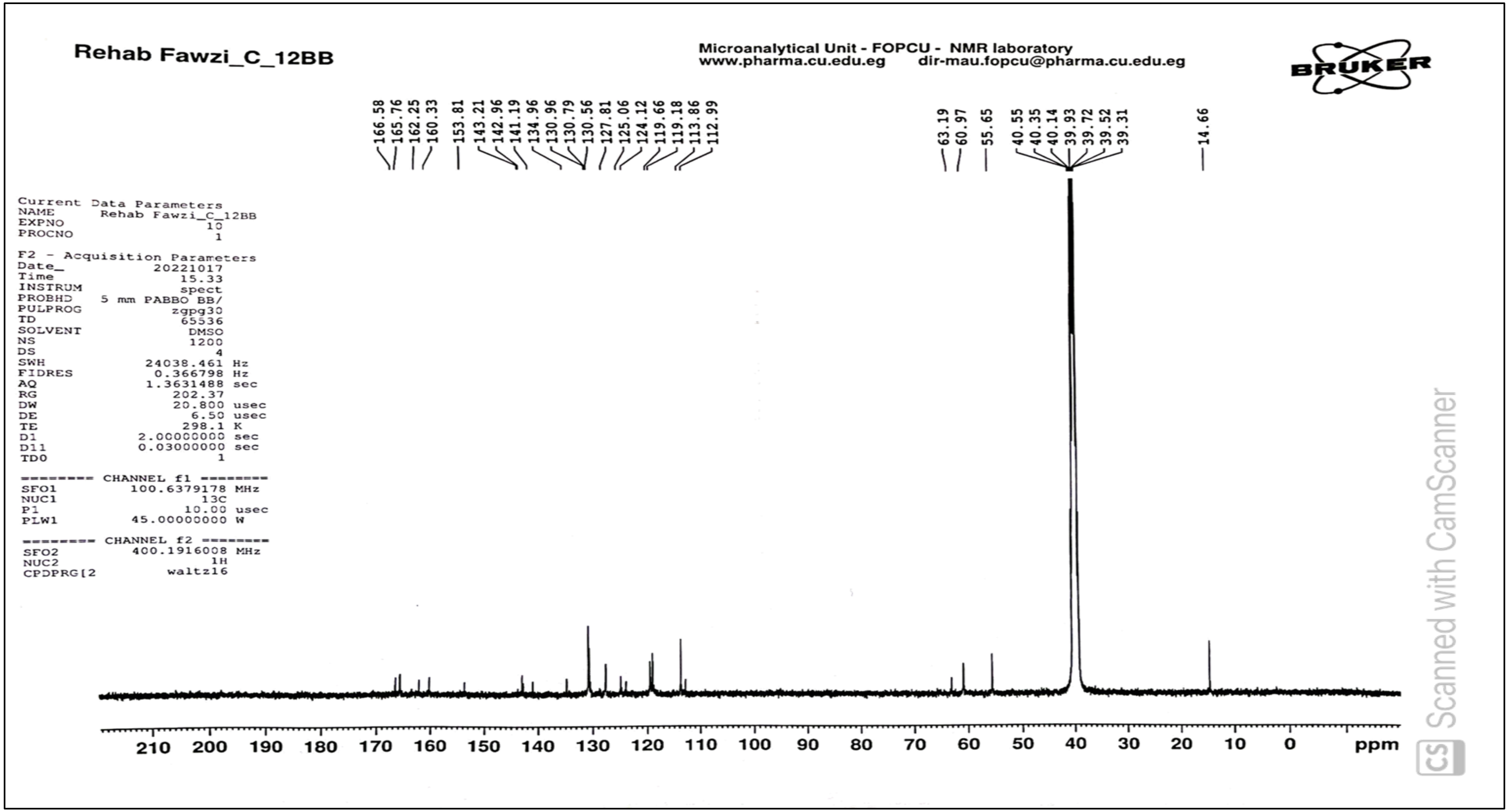

Scanned with CamScanner

Figure S76: ^{13}C NMR spectrum of 2-[(4-(Ethoxycarbonyl) phenyl) amino]-2-oxoethyl 3-(4-methoxyphenyl)-1-(4-sulfamoylphenyl)-1*H*-pyrazole-4-carboxylate **(5c)**

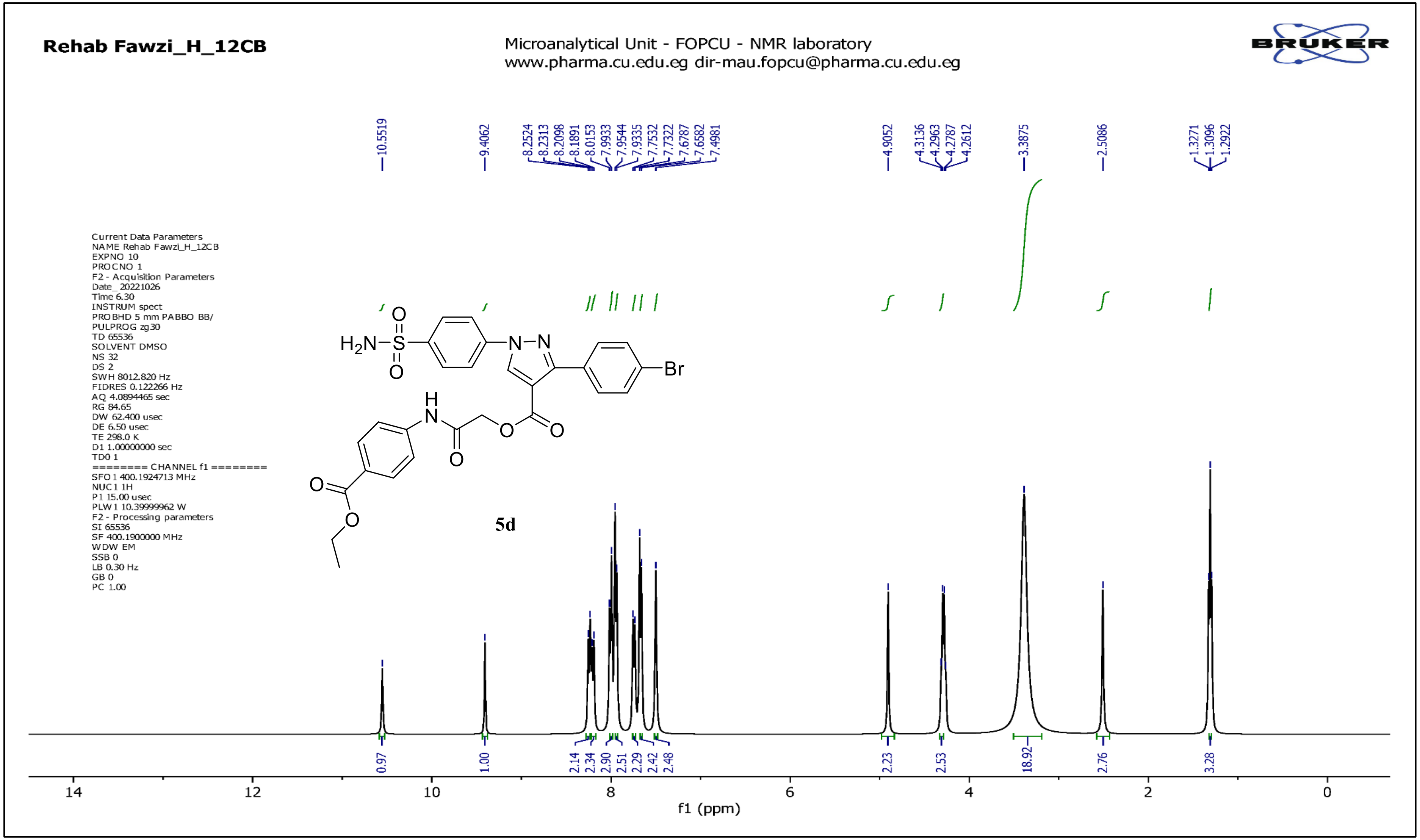

Figure S77: ^{1}H NMR spectrum of 2-[(4-(Ethoxycarbonyl) phenyl) amino]-2-oxoethyl 3-(4-bromophenyl)-1-(4-sulfamoylphenyl)-1*H*-pyrazole-4-carboxylate **(5d)**

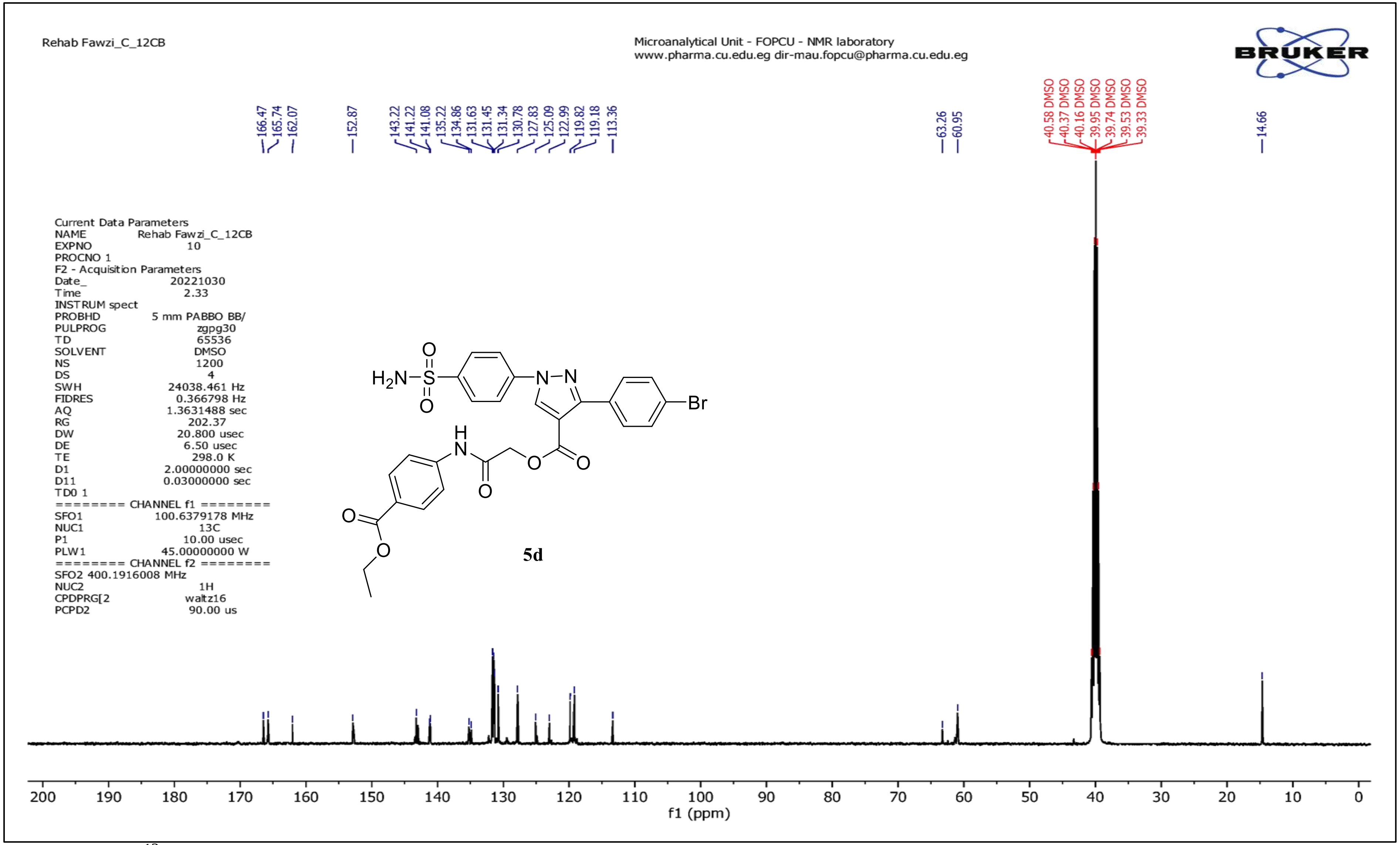

Figure S78: ^{13}C NMR spectrum of 2-[(4-(Ethoxycarbonyl) phenyl) amino]-2-oxoethyl 3-(4-bromophenyl)-1-(4-sulfamoylphenyl)-1*H*-pyrazole-4-carboxylate **(5d)**

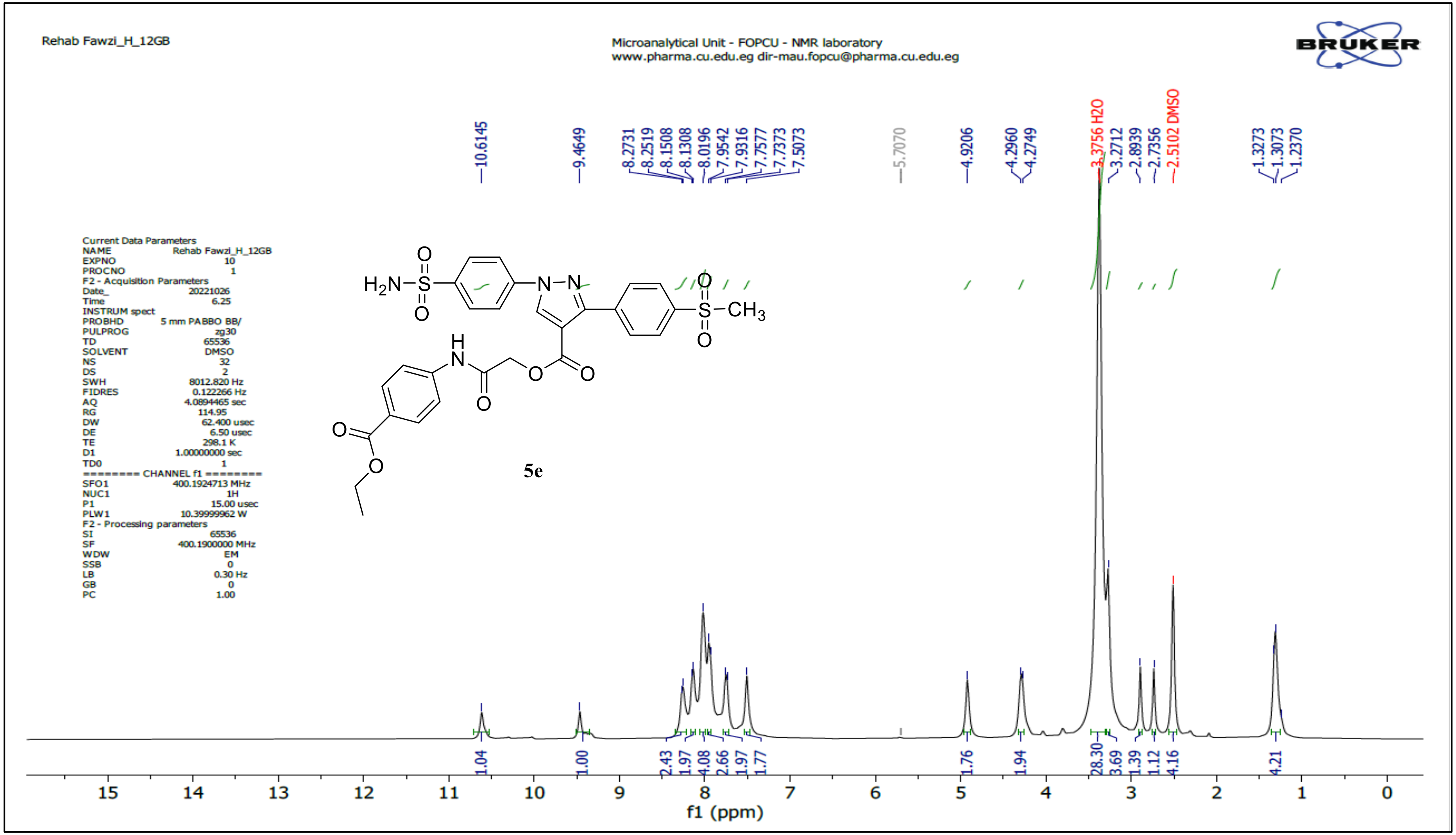

Figure S79: ^{1}H NMR spectrum of 2-[(4-(Ethoxycarbonyl) phenyl) amino]-2-oxoethyl 3-(4-(methylsulfonyl) phenyl)-1-(4-sulfamoylphenyl)-*1H*-pyrazole-4-carboxylate **(5e)**

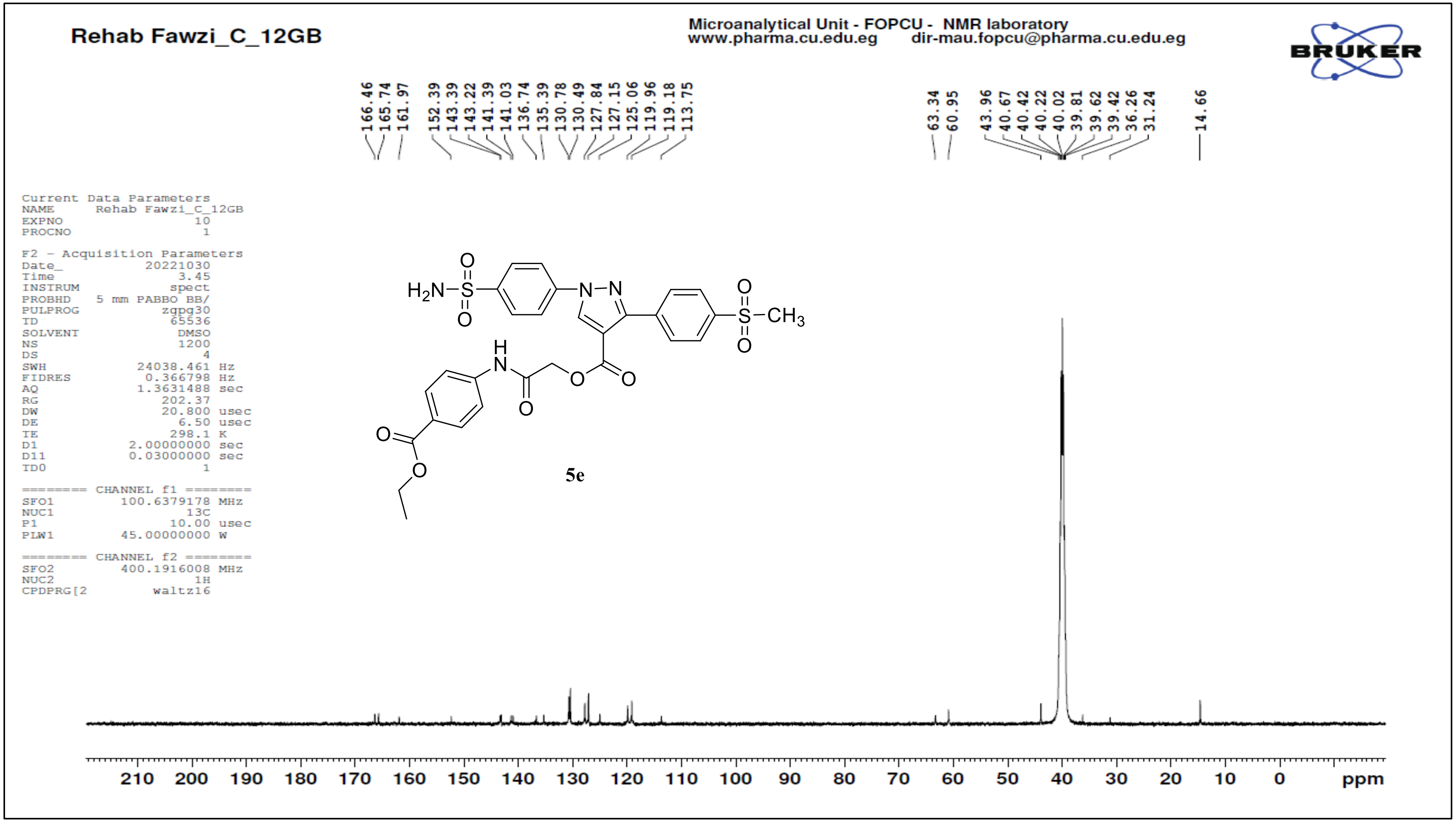

Figure S80: ^{13}C NMR spectrum of 2-[(4-(Ethoxycarbonyl) phenyl) amino]-2-oxoethyl 3-(4-(methylsulfonyl) phenyl)-1-(4-sulfamoylphenyl)-*1H*-pyrazole-4-carboxylate **(5e)**

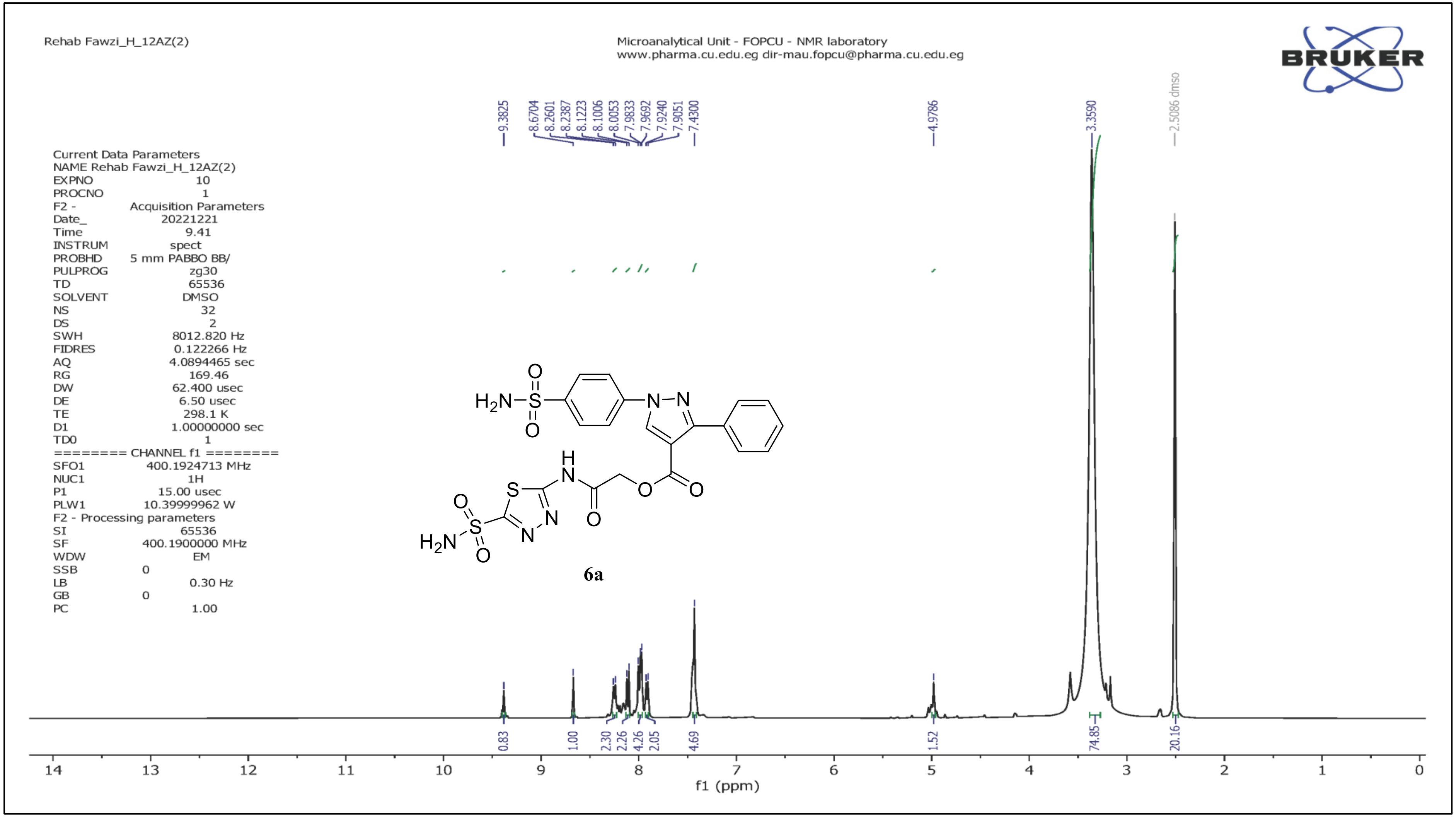

Figure S81: ^{1}H NMR spectrum of 2-Oxo-2-[(5-sulfamoyl-1,3,4-thiadiazol-2-yl) amino] ethyl 3-phenyl-1-(4-sulfamoylphenyl)-1*H*-pyrazole-4-carboxylate **(6a)**

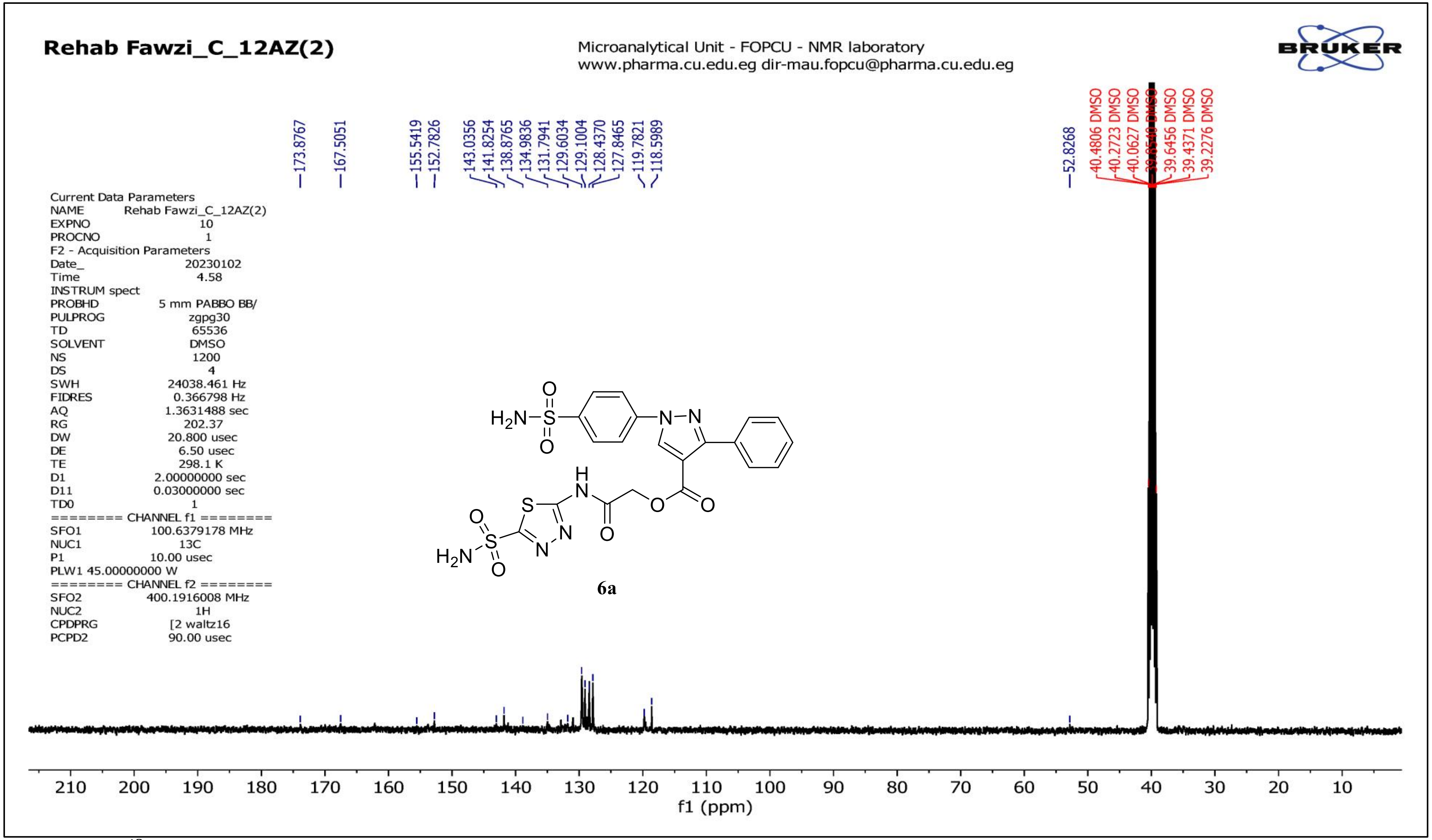

Figure S82: ^{13}C NMR spectrum of 2-Oxo-2-[(5-sulfamoyl-1,3,4-thiadiazol-2-yl) amino] ethyl 3-phenyl-1-(4-sulfamoylphenyl)-1*H*-pyrazole-4-carboxylate **(6a)**

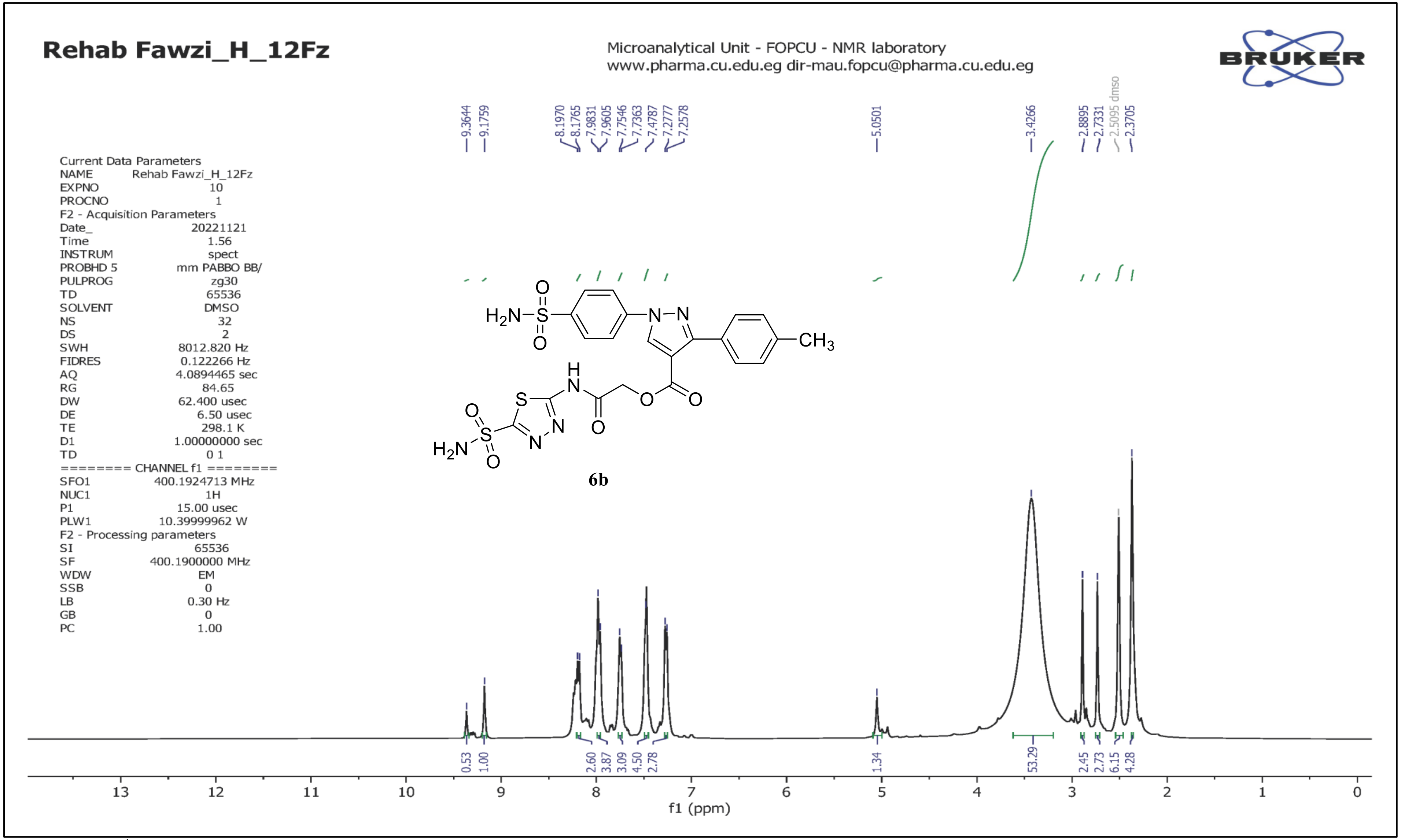

Figure S83: ^{1}H NMR spectrum of 2-Oxo-2-[(5-sulfamoyl-1,3,4-thiadiazol-2-yl) amino] ethyl 1-(4-sulfamoylphenyl)-3-(*p*-tolyl)-1*H*-pyrazole-4-carboxylate **(6b)**

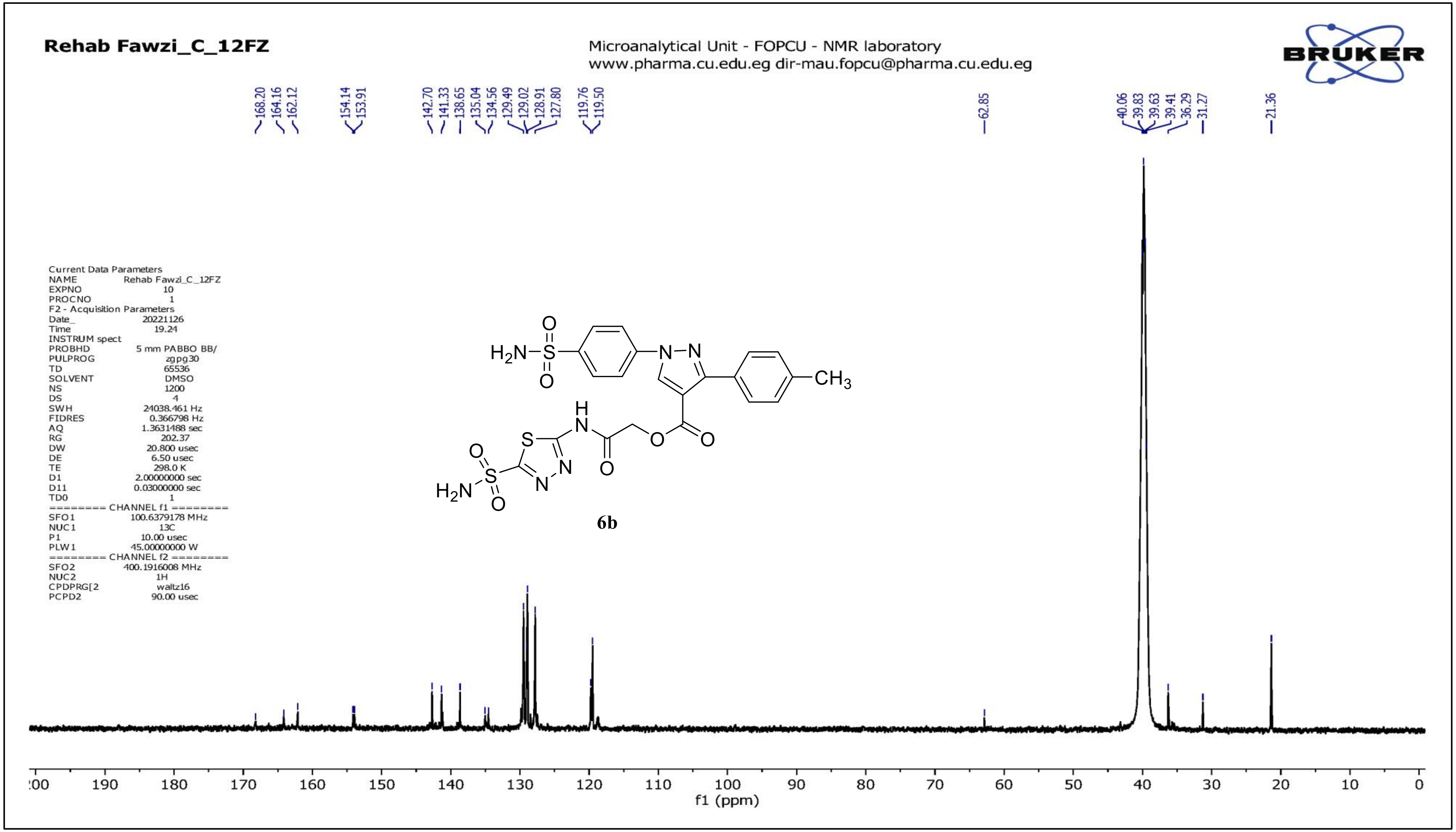

Figure S84: ^{13}C NMR spectrum of 2-Oxo-2-[(5-sulfamoyl-1,3,4-thiadiazol-2-yl) amino] ethyl 1-(4-sulfamoylphenyl)-3-(*p*-tolyl)-1*H*-pyrazole-4-carboxylate **(6b)**

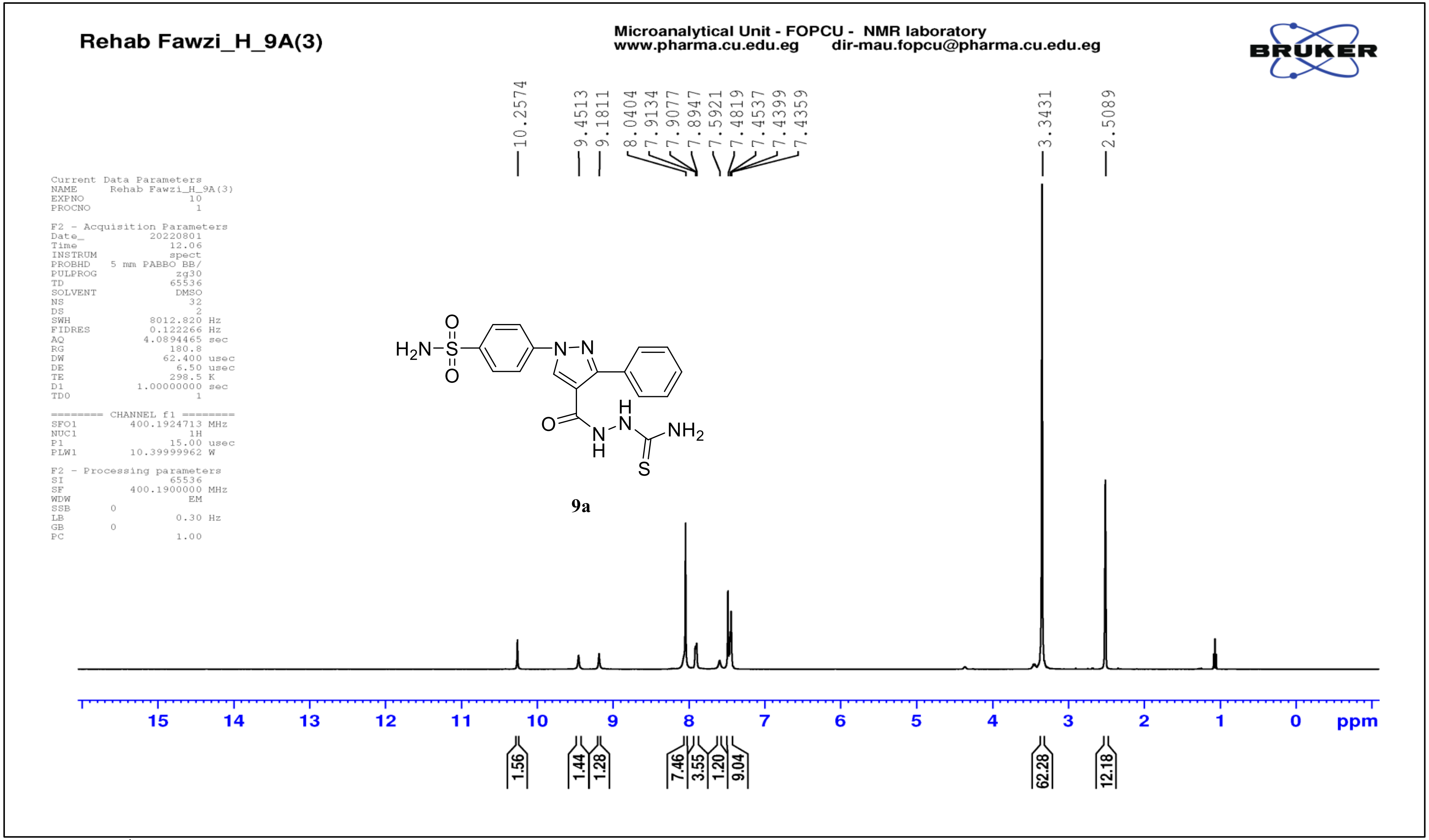

Figure S85: ^{1}H NMR spectrum of 2-[3-Phenyl-1-(4-sulfamoylphenyl)-1*H*-pyrazole-4-carbonyl] hydrazine-1-carbothioamide **(9a)**

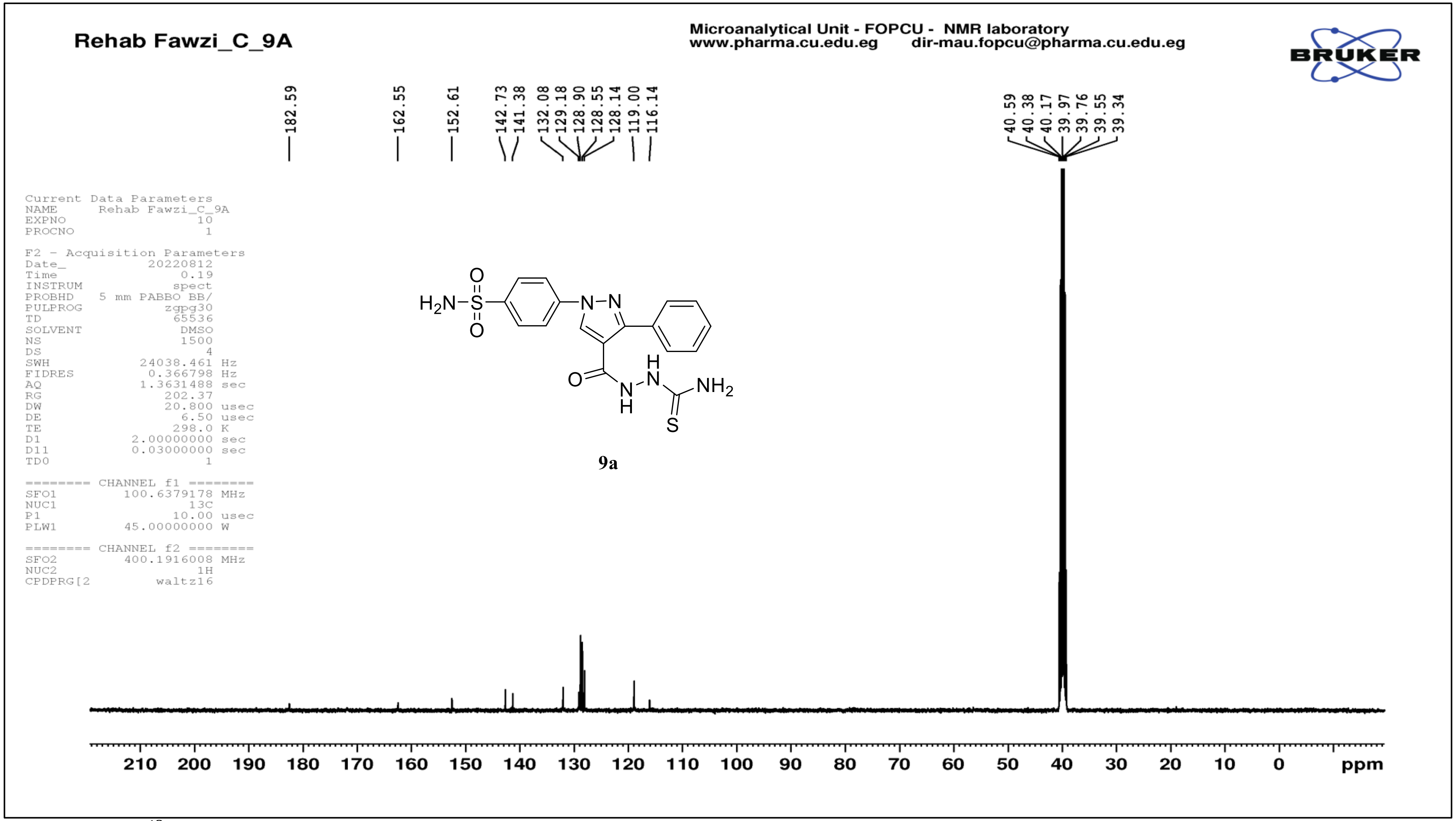

Figure S86: ^{13}C NMR spectrum of 2-[3-Phenyl-1-(4-sulfamoylphenyl)-1*H*-pyrazole-4-carbonyl] hydrazine-1-carbothioamide **(9a)**

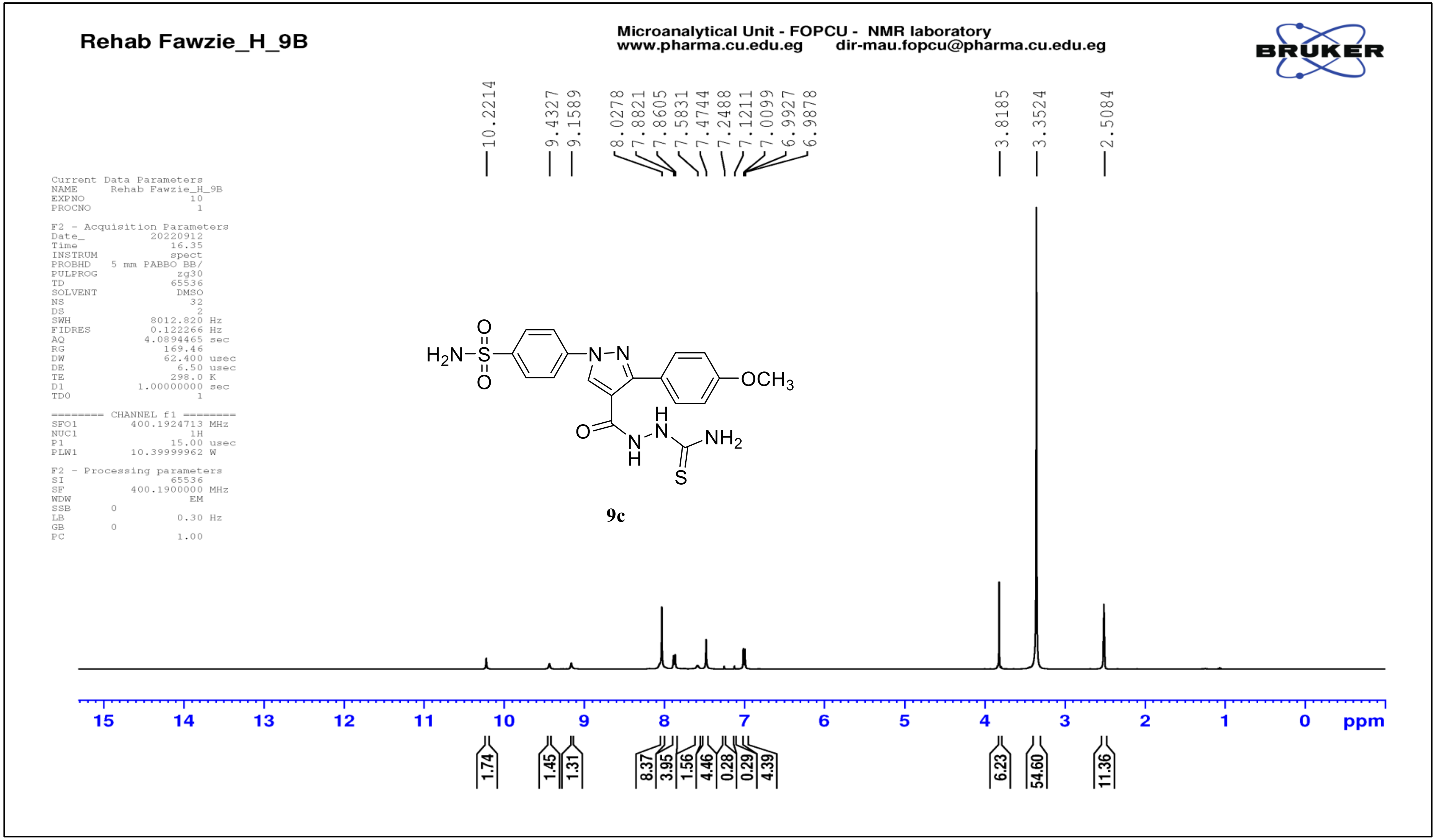

Figure S87: ^{1}H NMR spectrum of 2-[3-(4-Methoxyphenyl)-1-(4-sulfamoylphenyl)-1*H*-pyrazole-4-carbonyl] hydrazine-1-carbothioamide **(9c)**

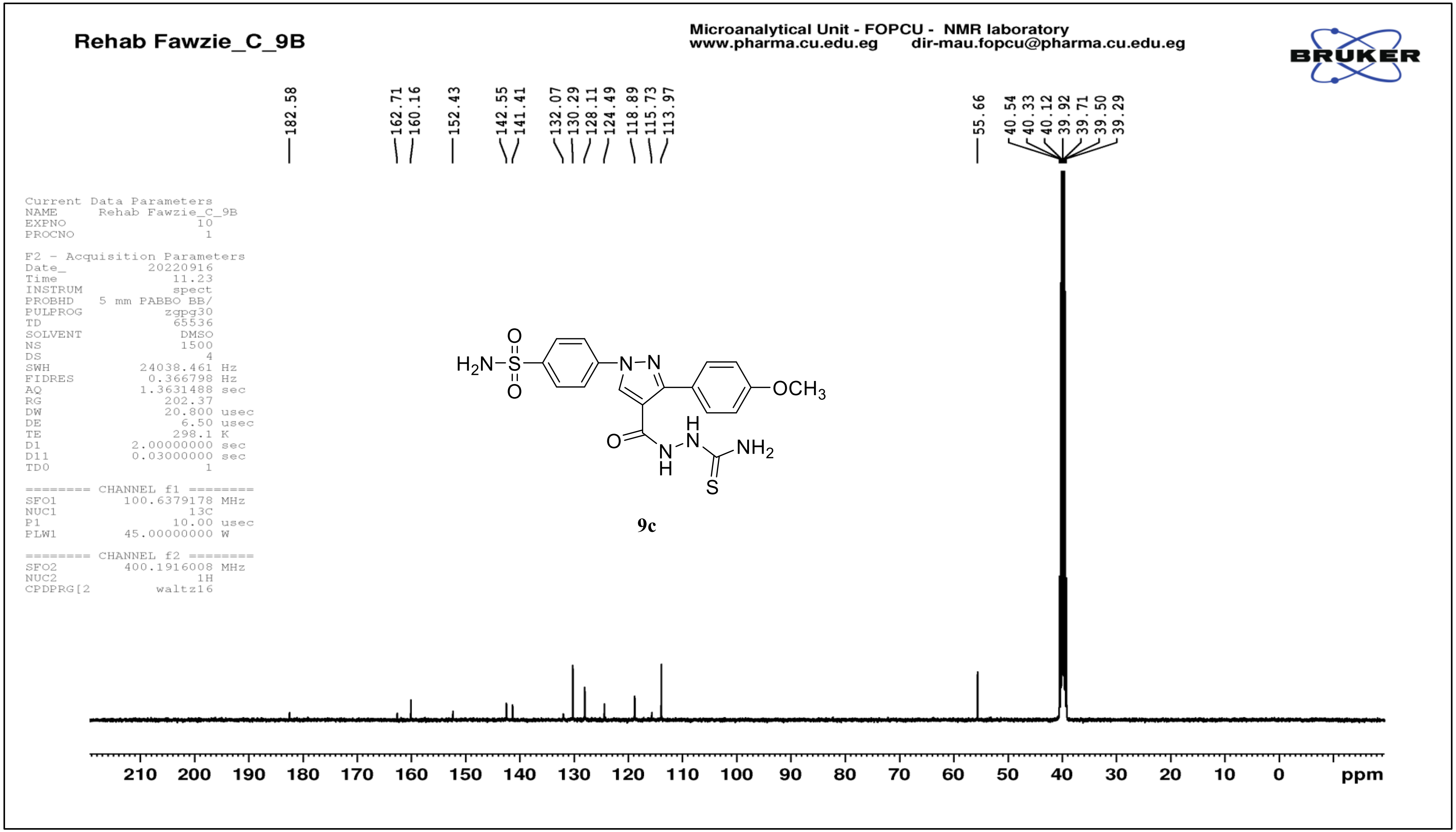

Figure S88: ^{13}C NMR spectrum of 2-[3-(4-Methoxyphenyl)-1-(4-sulfamoylphenyl)-1*H*-pyrazole-4-carbonyl] hydrazine-1-carbothioamide **(9c)**

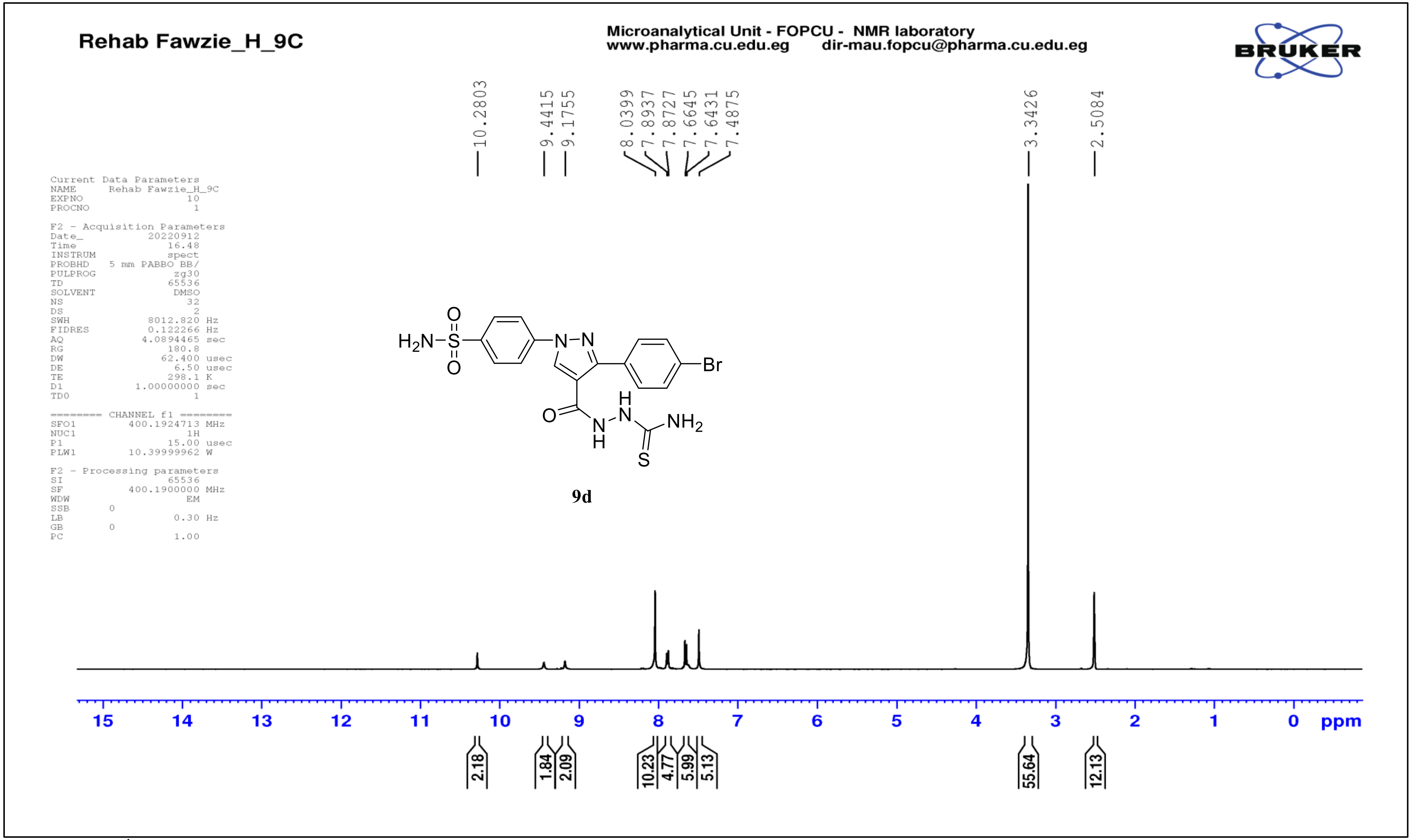

Figure S89: ^{1}H NMR spectrum of 2-[3-(4-Bromophenyl)-1-(4-sulfamoylphenyl)-1*H*-pyrazole-4-carbonyl] hydrazine-1-carbothioamide **(9d)**

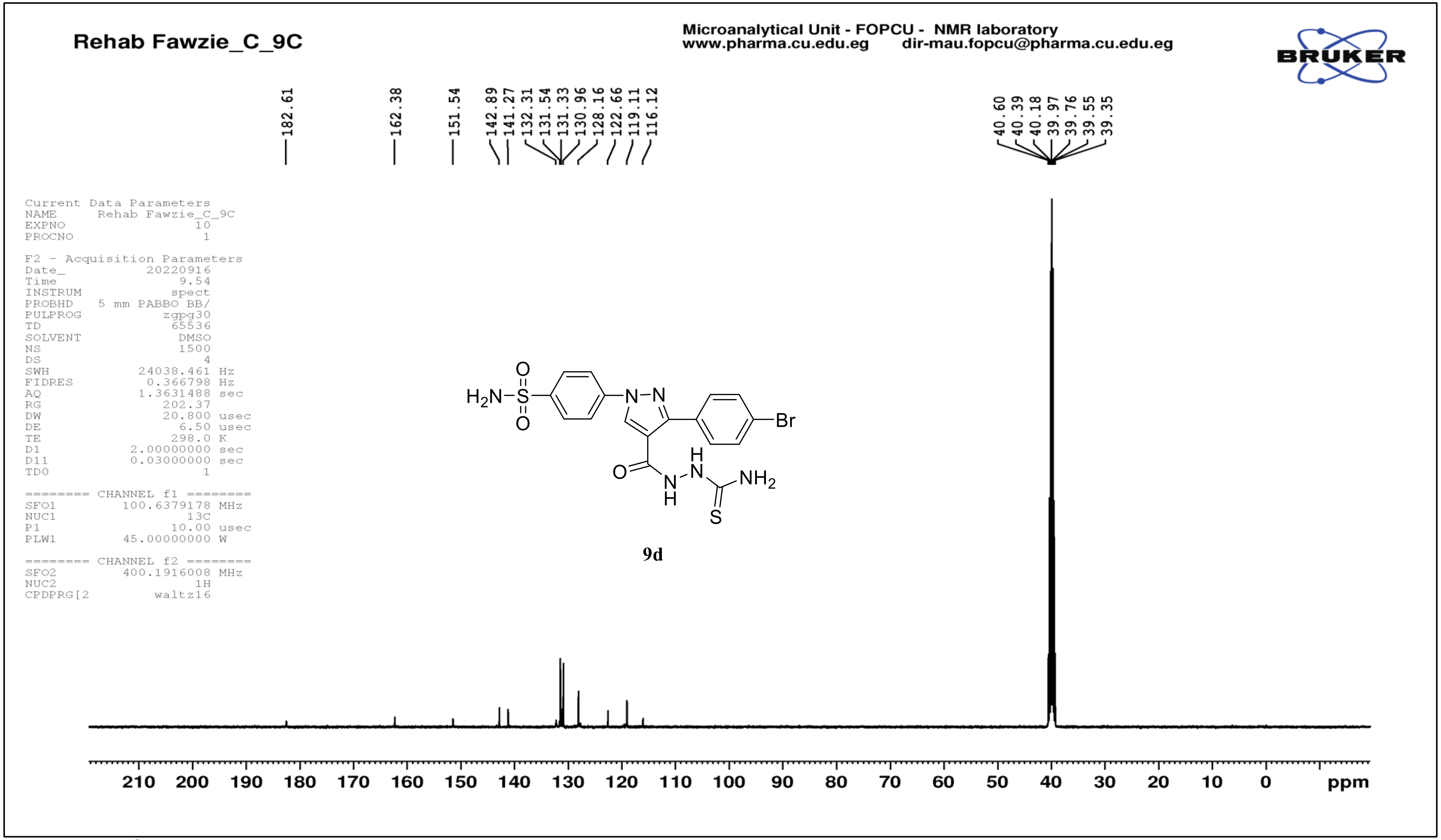

Figure S90: ^{13}C NMR spectrum of 2-[3-(4-Bromophenyl)-1-(4-sulfamoylphenyl)-1*H*-pyrazole-4-carbonyl] hydrazine-1-carbothioamide **(9d)**

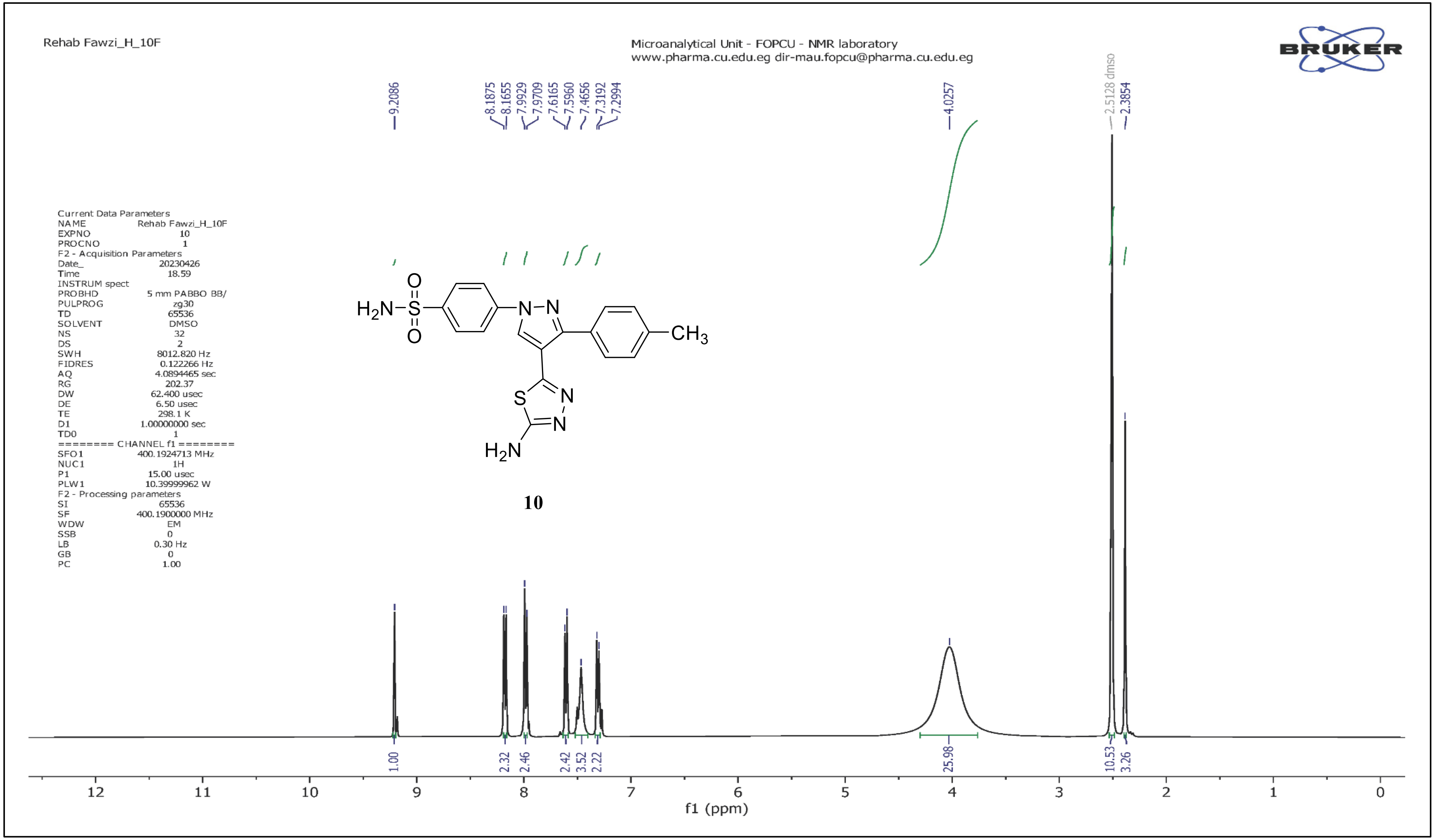

Figure S91: ^{1}H NMR spectrum of 4-[4-(5-Amino-1,3,4-thiadiazol-2-yl)-3-(*p*-tolyl)-1*H*-pyrazol-1-yl] benzenesulfonamide **(10)**

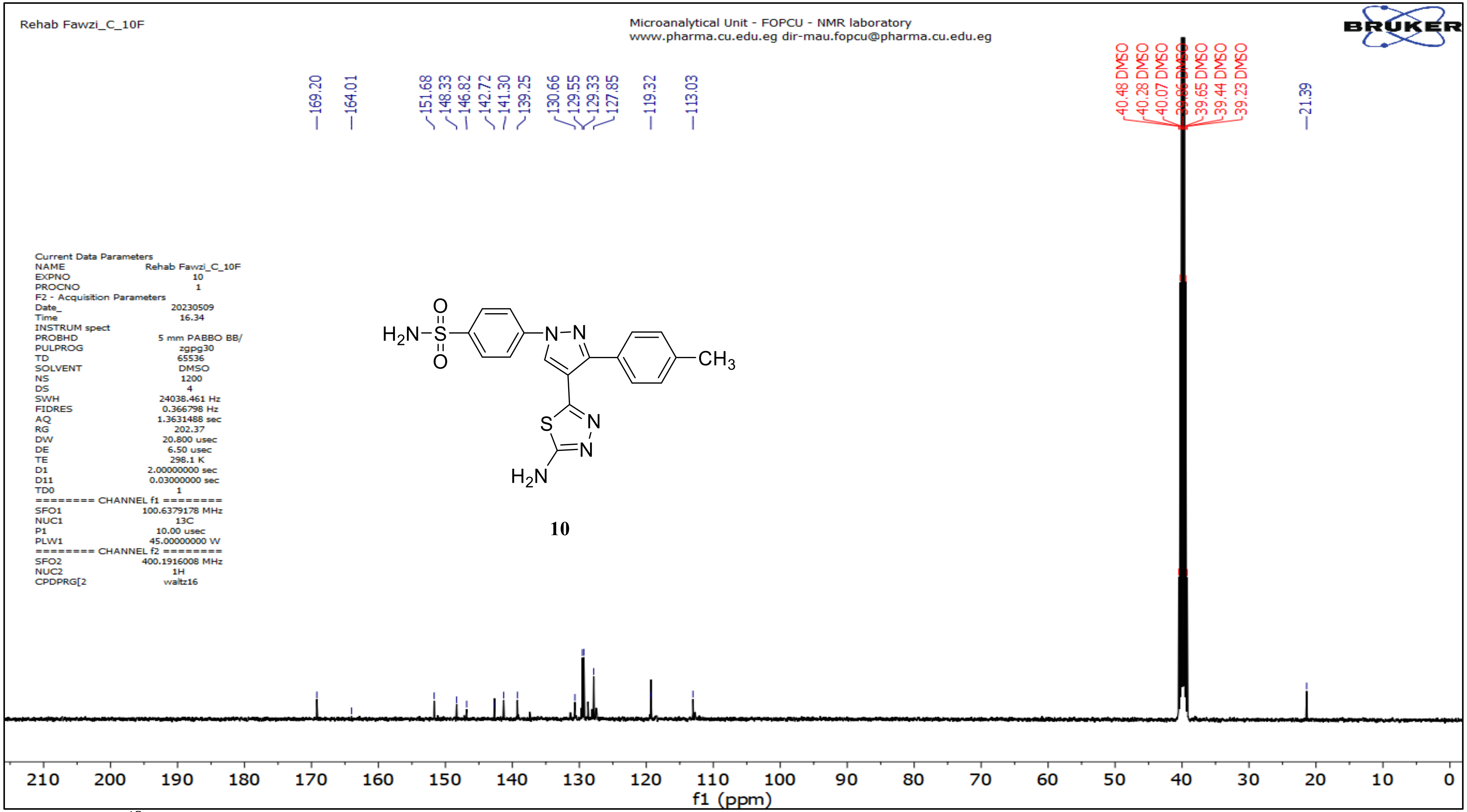

Figure S92: ^{13}C NMR spectrum of 4-[4-(5-Amino-1,3,4-thiadiazol-2-yl)-3-(*p*-tolyl)-1*H*-pyrazol-1-yl] benzenesulfonamide **(10)**

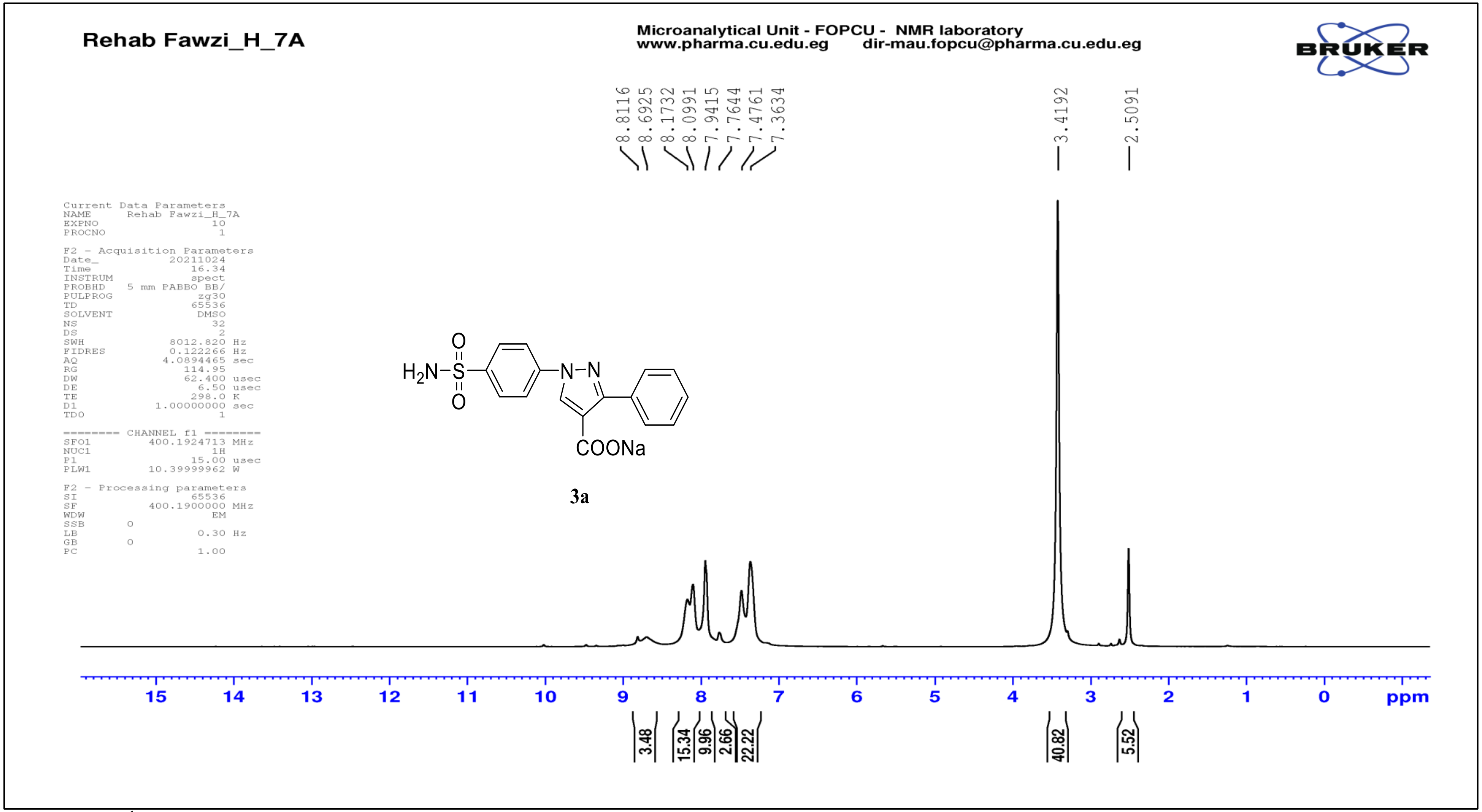

Figure S93: ^{1}H NMR spectrum Sodium 3-phenyl-1-(4-sulfamoylphenyl)-1*H*-pyrazole-4-carboxylate **(3a)**

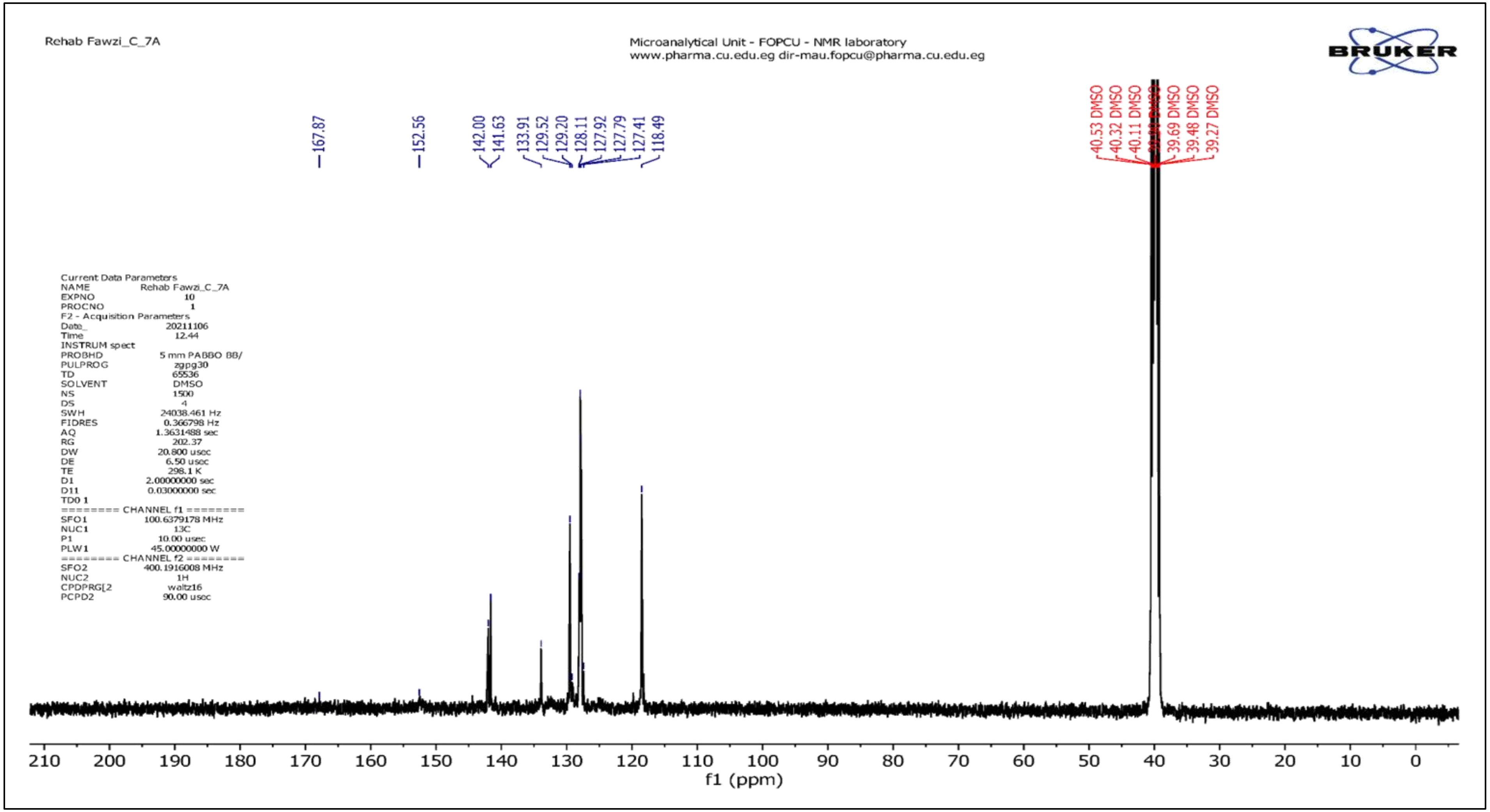

Figure S94: ^{13}C NMR spectrum Sodium 3-phenyl-1-(4-sulfamoylphenyl)-1*H*-pyrazole-4-carboxylate **(3a)**

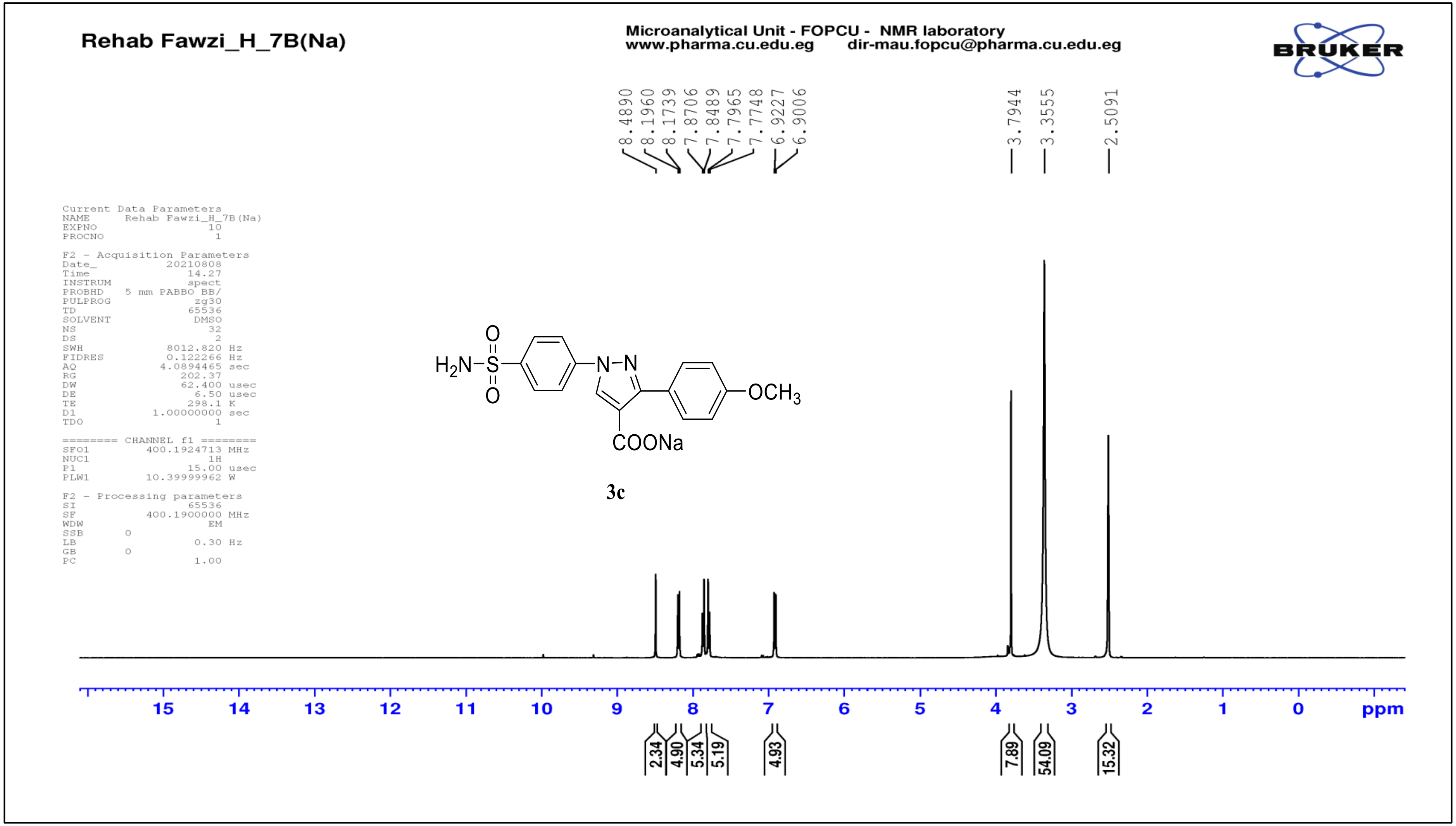

Figure S95: ^{1}H NMR spectrum Sodium 3-(4-methoxyphenyl)-1-(4-sulfamoylphenyl)-*1H*-pyrazole-4-carboxylate **(3c)**

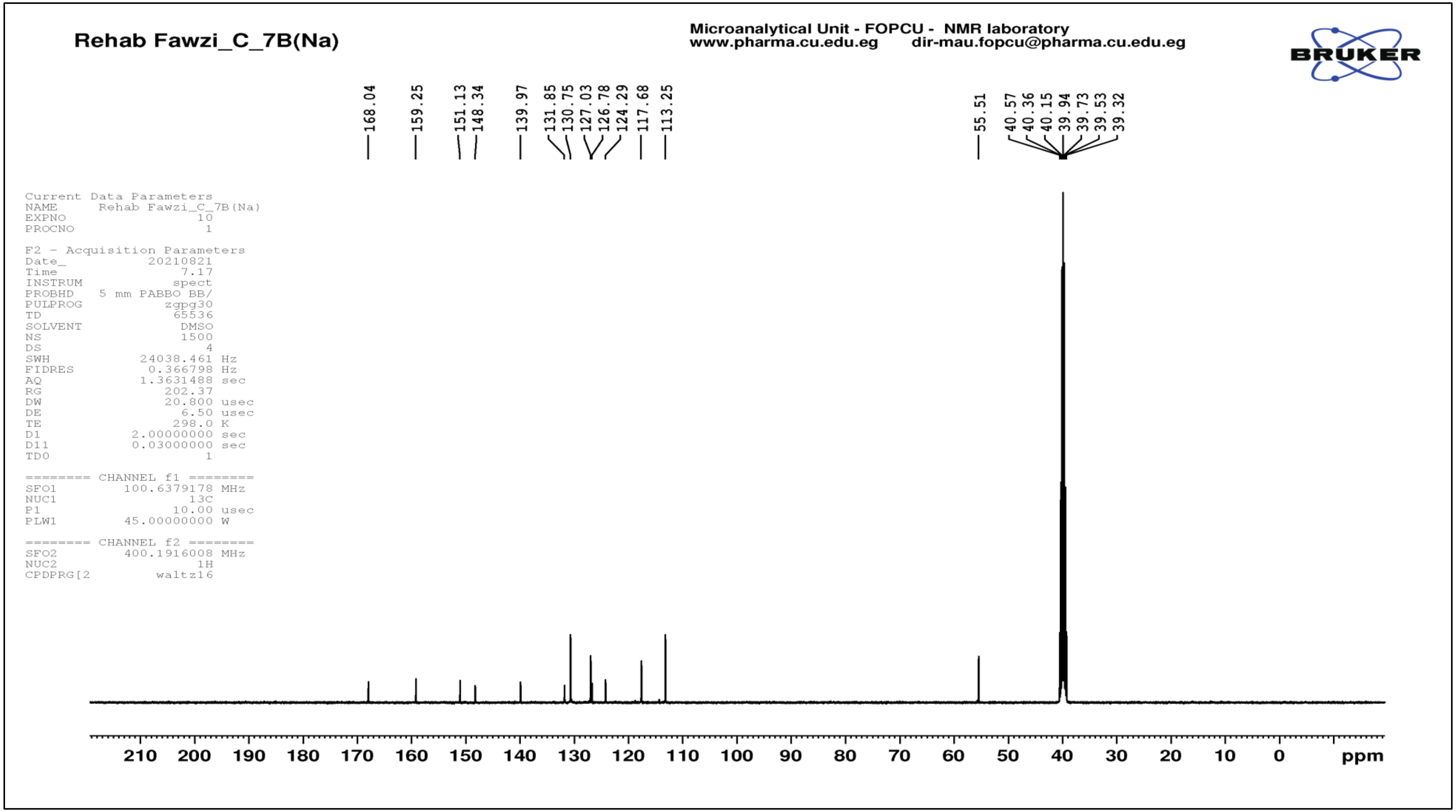

Figure S96: ^{13}C NMR spectrum Sodium 3-(4-methoxyphenyl)-1-(4-sulfamoylphenyl)-*1H*-pyrazole-4-carboxylate **(3c)**

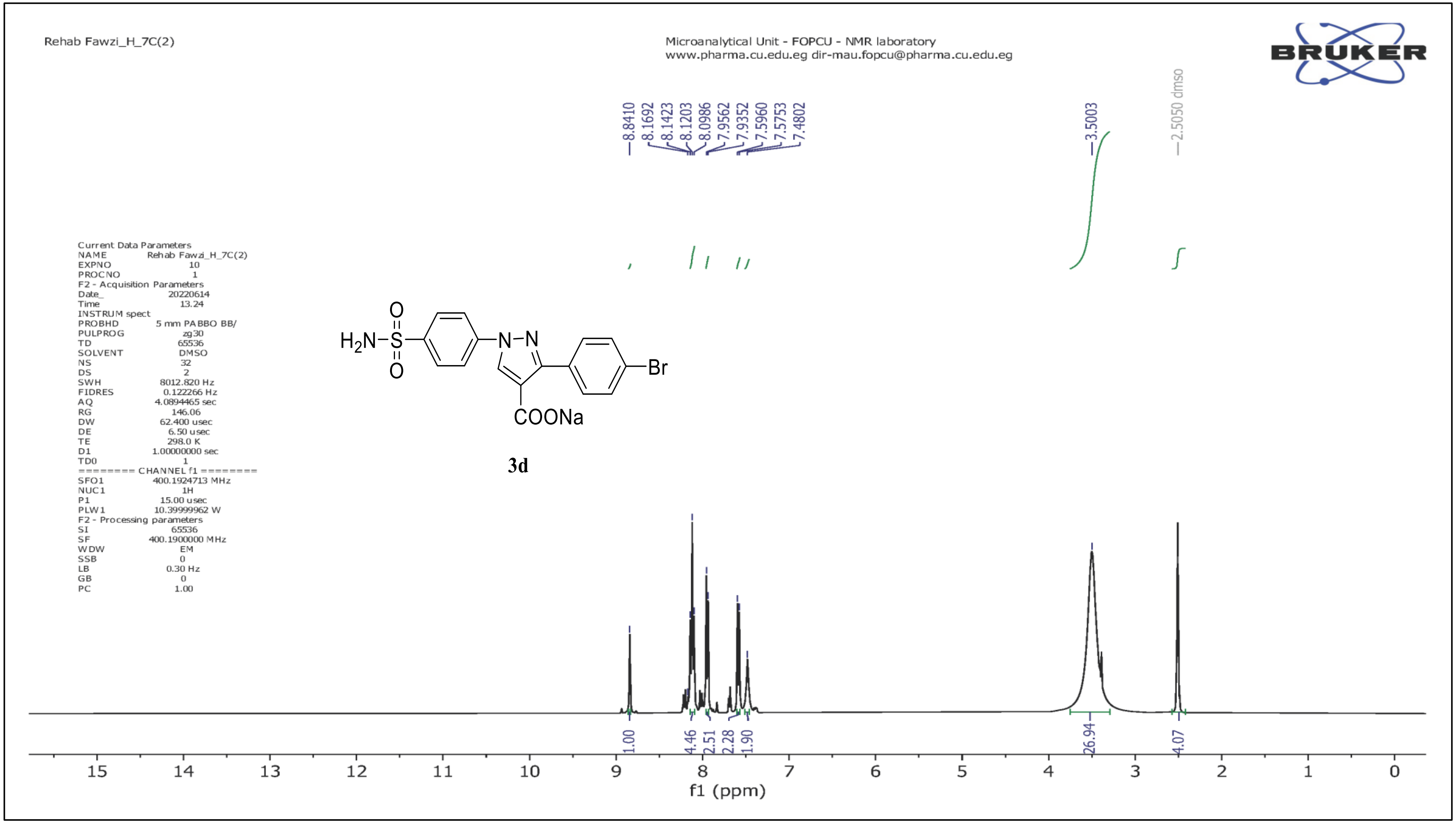

Figure S97: ^{1}H NMR spectrum Sodium 3-(4-bromophenyl)-1-(4-sulfamoylphenyl)-*1H*-pyrazole-4-carboxylate **(3d)**

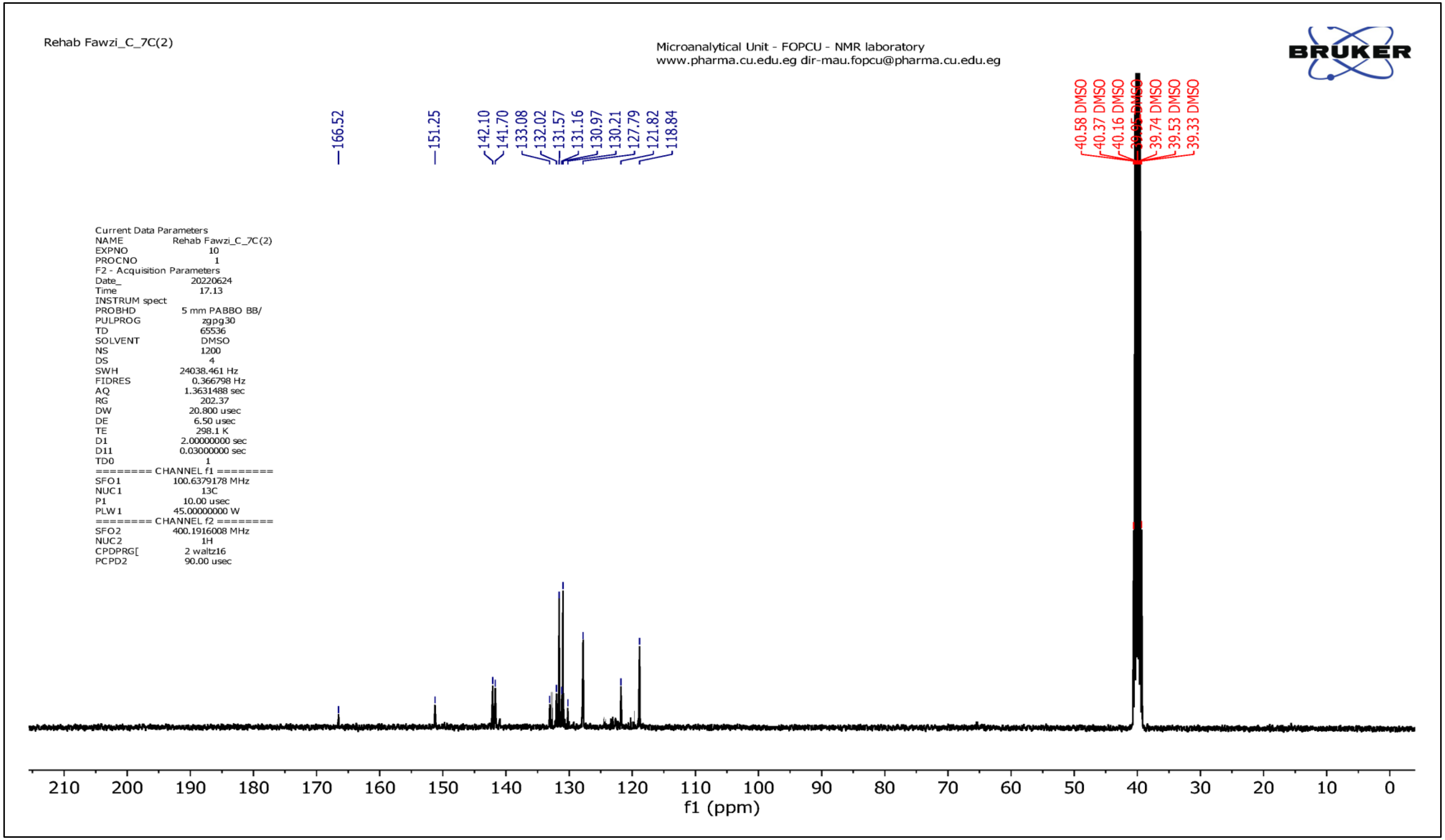

Figure S98: ^{13}C NMR spectrum Sodium 3-(4-bromophenyl)-1-(4-sulfamoylphenyl)-*1H*-pyrazole-4-carboxylate **(3d)**

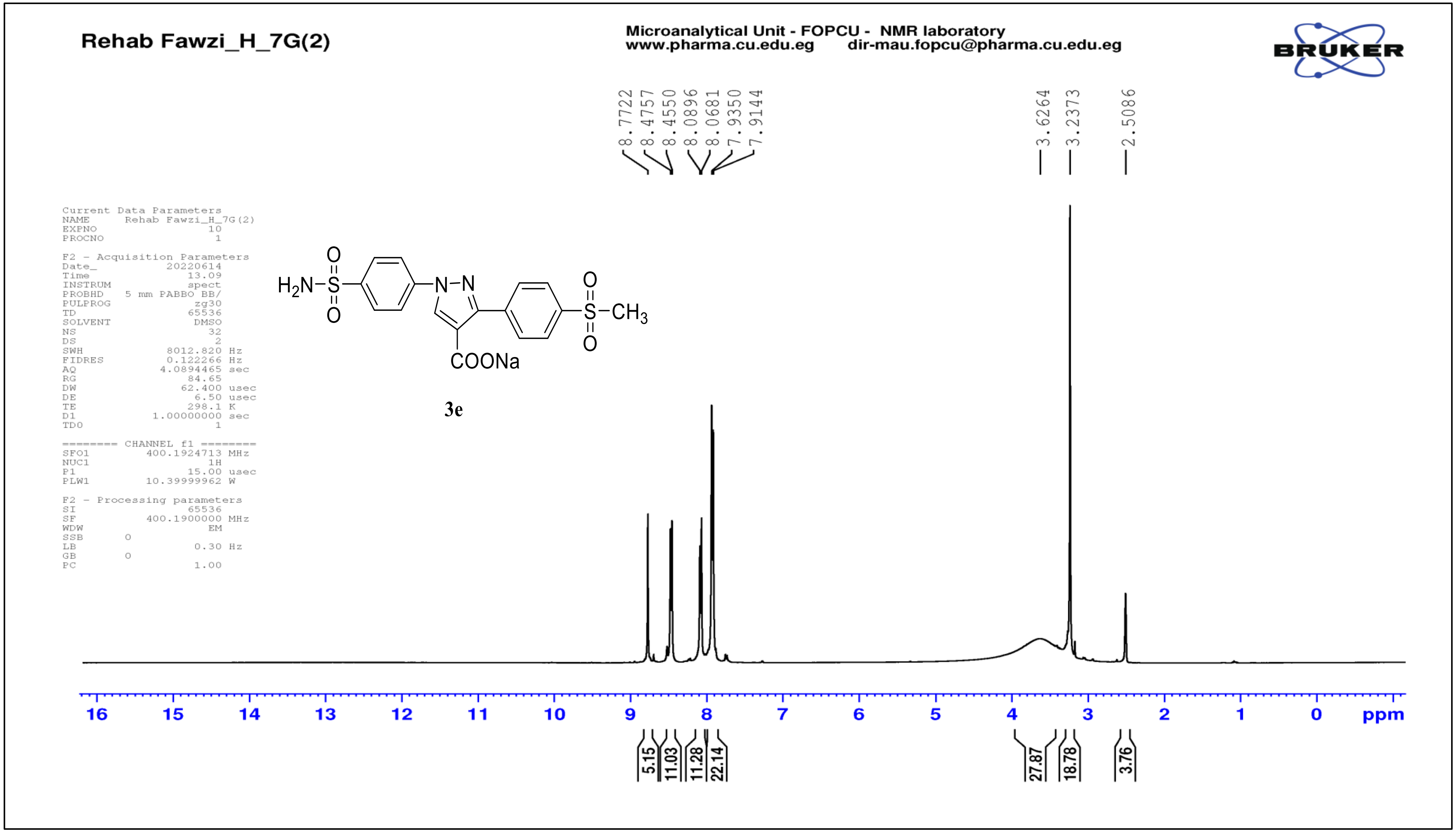

Figure S99: ^{1}H NMR spectrum Sodium 3-(4-(methylsulfonyl) phenyl)-1-(4-sulfamoylphenyl)-*1H*-pyrazole-4-carboxylate **(3e)**

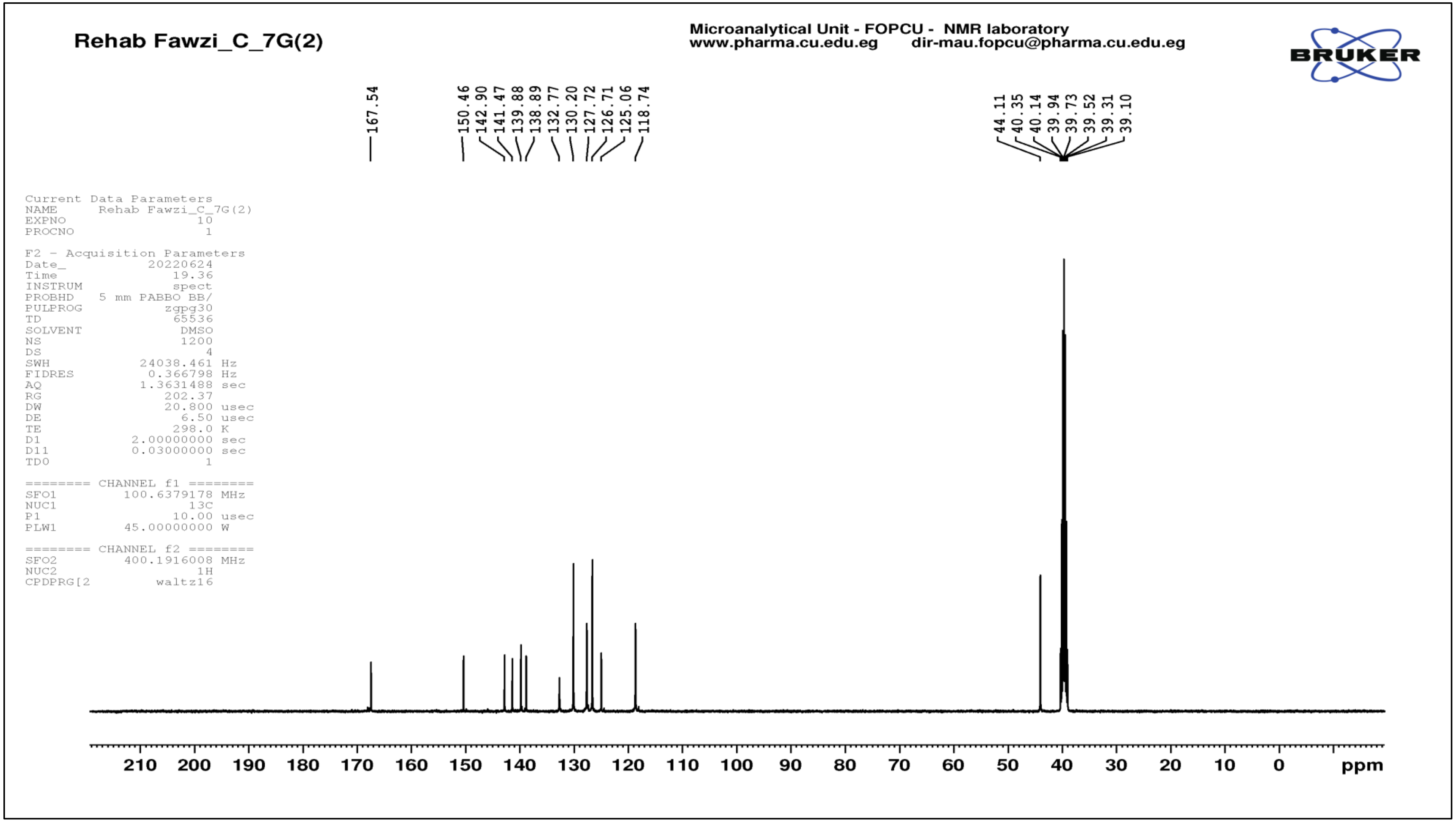

Figure S100: ^{13}C NMR spectrum Sodium 3-(4-(methylsulfonyl) phenyl)-1-(4-sulfamoylphenyl)-*1H*-pyrazole-4-carboxylate **(3e)**

<u>HPLC chromatograph for compound 5b</u>

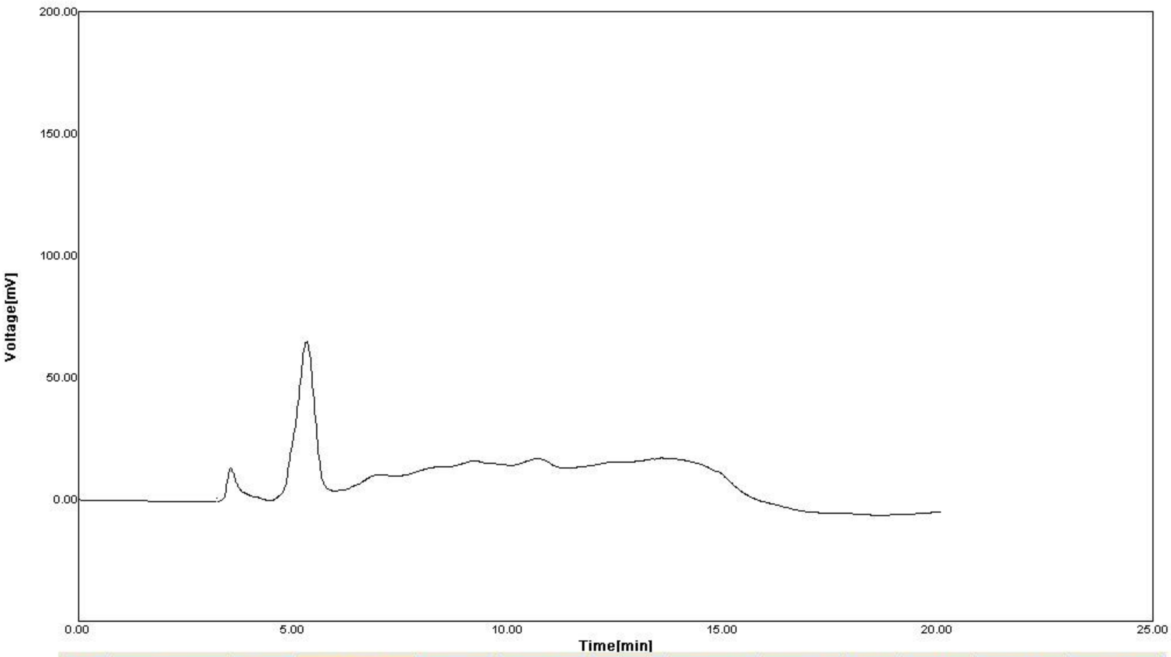

#	Name	RT[min]	Area[mV*sec]	Area%	Height[mV]	Height%	Amount	Amount%	Code	Width[s]	Half Width[s]	TP
1		3.8500	141.4878	7.22	10.7746	14.65	0.00	0.00	BB	32.00	13.00	1750.9
2		5.4000	1819.3132	92.78	62.7489	85.35	0.00	0.00	BB	87.00	27.00	798.5
Sum			1960.8010		73.5235		0.00000					

Figure S101: HPLC chromatogram for compound **5b.**

Column: C18

MP: 50 mM KH_2PO_4: ACN (60:40, v/v)

Detection: UV at 280 nm

Flow rate: 1 mL/min

Injection volume: 20 microliter

Drug concentration: 10 mcg/mL
